# Voluntary Activity Wheel Running Improves Hyperammonaemia‐Induced Skeletal Muscle Molecular and Metabolic Perturbations in Mice

**DOI:** 10.1002/jcsm.70031

**Published:** 2025-08-04

**Authors:** Annette Bellar, Avinash Kumar, Khaviyaa Chandramohan, Saurabh Mishra, Muaz Alsabbagh‐Alchirazi, Artem Astafev, Pugazhendhi Kannan, Amy Attaway, Roman Kondratov, Thomas Jaramillo, Lopa Mishra, Takhar Kasumov, Nicole Welch, Srinivasan Dasarathy

**Affiliations:** ^1^ Department of Inflammation and Immunity Lerner Research Institute Cleveland Clinic Cleveland Ohio USA; ^2^ Department of Gastroenterology and Human Nutrition All India Institute of Medical Sciences New Delhi India; ^3^ Biological, Geological, and Environmental Science Department, Center for Gene Regulation in Health and Disease Cleveland State University Cleveland Ohio USA; ^4^ Department of Pulmonary Medicine Cleveland Clinic Cleveland Ohio USA; ^5^ Lerner Research Core Services Lerner Research Institute Cleveland Clinic Cleveland Ohio USA; ^6^ Feinstein Institutes of Medical Research Northwell Health New York New York USA; ^7^ Department of Pharmaceutical Sciences Northeast Ohio Medical University Rootstown Ohio USA; ^8^ Department of Gastroenterology and Hepatology Cleveland Clinic Cleveland Ohio USA

**Keywords:** exercise, hyperammonaemia, mitochondrial function, sarcopenia, voluntary wheel running

## Abstract

**Aim:**

Voluntary exercise improves clinical outcomes in healthy subjects, but increased muscle ammoniagenesis may limit beneficial responses during hyperammonaemia in chronic diseases. Responses to 4‐weeks voluntary wheel running (VWR) were compared with usual activity (UA) to determine if hyperammonaemia alters VWR responses and if VWR alters muscle responses to hyperammonaemia.

**Methods:**

Eight‐ to 10‐week‐old male C57BL/6J mice were treated with 6 weeks of subcutaneous infusion of 2.5 mmol kg^−1^ day^−1^ ammonium acetate (AmAc) or vehicle (PBS) via an osmotic pump. Two weeks after the start of infusion, mice were assigned to the intervention (VWR or UA). Wheel runs were measured, and weekly average rotations, distance, and circadian patterns were analysed. Indirect calorimetry was performed pre‐ and post‐intervention. Mice were euthanized 4 weeks after the start of VWR/UA, and organs (including muscles) were harvested, weighed, and muscle histomorphometry performed for fibre diameter/type. Protein synthesis by ex vivo puromycin incorporation, autophagy markers, expression of signalling proteins (mTORC1 pathway, eukaryotic initiation factor‐2‐α phosphorylation), and ammonia disposal enzymes were quantified by immunoblots. Mitochondrial oxidative function was measured by high‐sensitivity respirofluorometry using substrate, uncoupler, inhibitor, and titration protocols. Fluorometric assays were done for ammonia measurements.

**Results:**

Gastrocnemius muscle mass (*p* < 0.01), muscle fibre area (*p* < 0.01), and grip strength were lower in AmAc‐UA than in PBS‐UA mice and higher with VWR than UA in AmAc mice (*p* < 0.001). Expression of electron transport chain proteins and some components of mitochondrial oxidative function were less (*p* < 0.05 or less) in AmAc‐UA than PBS‐UA, and these perturbations were reversed in the AmAc‐VWR mice (*p* < 0.05 or less). Global muscle protein synthesis (*p* < 0.05) and components of the mTORC1 pathway expression (*p* < 0.05) were higher, while myostatin expression was lower with VWR than UA in AmAc mice (*p* < 0.05). Expression of autophagy markers P62 and LC3‐II was not different with VWR or UA in AmAc mice, while Beclin1 was higher in VWR compared with UA, regardless of treatment group (*p* < 0.001). Expression of muscle ammonia disposal pathway enzymes, including glutamate dehydrogenase and pyrroline‐5‐carboxylate synthase, was higher (*p* ≤ 0.05) in AmAc‐UA versus PBS‐UA and increased in only PBS‐VWR mice (*p* < 0.05).

**Conclusion:**

VWR reverses hyperammonaemia‐induced sarcopenia, protein synthesis/autophagy signalling perturbations, and mitochondrial oxidative dysfunction. Muscle mass, grip strength, signalling, and mitochondrial responses to VWR were not affected by hyperammonaemia. Increased expression of enzymes involved in the ammonia disposal pathway in skeletal muscle may be an adaptive response to hyperammonaemia. These data provide the rationale for exercise programmes in chronic diseases, including cirrhosis, even with hyperammonaemia.

## 1 Introduction

Endurance exercise improves cardiopulmonary fitness, while the impact on muscle mass and strength is more modest [1]. Despite the recognized benefits of exercise, patients with chronic diseases have low adherence to exercise training programmes due primarily to fatigue [2]. Increased skeletal muscle ammoniagenesis during exercise is a mediator of fatigue [2, 3, 4]. Ammonia is an endogenous cytotoxic metabolite that also contributes to sarcopenia and contractile dysfunction with physical frailty [4, 5]. The principal mechanism of ammonia disposal is hepatocyte ureagenesis (Reference S1); however, during perturbations in ammonia metabolism that occur with chronic diseases, the skeletal muscle becomes a metabolic partner for ammonia disposal [6]. Skeletal muscle hyperammonaemia results in a sarcopenic phenotype, impaired mTORC1 signalling, dysregulated proteostasis, and mitochondrial oxidative dysfunction [7, 8, 9, 10]. Despite the potential interactions between exercise and ammonia, how hyperammonaemia alters exercise responses and if exercise modifies tissue responses to ammonia are not known and were evaluated in these studies.

Muscle contraction and protein synthesis are bioenergetically demanding functions that are impaired during hyperammonaemia due to mitochondrial oxidative dysfunction [7, 10]. Therefore, exercise‐induced ammoniagenesis can worsen hyperammonaemia‐induced fatigue and sarcopenia [4, 11] (Reference S2). Furthermore, untargeted phosphoproteomics analyses suggest that activity‐induced ammoniagenesis can limit beneficial responses to exercise (Reference S3). However, there are no direct experimental data that hyperammonaemia modulates exercise responses or if exercise modifies ammonia metabolism during hyperammonaemia. Cirrhosis of the liver is a chronic disease with consistent muscle hyperammonaemia, a high prevalence of sarcopenia, and mitochondrial oxidative dysfunction [5]. We used a validated mouse model of hyperammonaemia and sarcopenia in cirrhosis [6] to test if skeletal muscle exercise responses are altered during hyperammonaemia. This model allows for the evaluation of the consequences of perturbed ammonia metabolism without the confounding effects of cytokine and necroinflammatory consequences of tissue injury in cirrhosis and chronic diseases.

Exercise responses have been studied in a number of preclinical models that include forced treadmill run/swim to exhaustion, which result in stress responses and do not replicate human behaviour of voluntary physical activity [12]. A Voluntary Wheel Running (VWR) protocol avoids the limitations of treadmill/swim to exhaustion with reduced animal handling and avoids the stress of forced exercise regimens [13]. We used a mouse model of hyperammonaemia, with muscle concentrations of ammonia that replicate those in human cirrhosis [6], to determine phenotypic, functional, and skeletal muscle molecular responses. Our data show that hyperammonaemia causes reduced muscle mass and strength, lowers mitochondrial oxidative function, decreases muscle protein synthesis, impairs the mammalian target of rapamycin complex 1 (mTORC1) signalling, and increases myostatin (MSTN) expression, which were reversed by VWR. Finally, during VWR, hyperammonaemia did not impair signalling responses, muscle mass, or grip strength responses despite a further increase in muscle and blood ammonia concentrations.

## 2 Methods and Reagents

### 2.1 Chemicals

Details of the reagents and sources are shown in Table S1.

### 2.2 Animal Studies

All animal studies were approved by the Institutional Animal Care and Use Committee (IACUC) at the Cleveland Clinic (IACUC No: 2019‐2268). Studies were performed in male C57BL/6J mice, 8–9 weeks of age, which were purchased from Jackson Laboratories (Bar Harbor, Maine, USA).

Body composition, magnetic resonance imaging (EchoMRI), whole‐body metabolic measures, and food intake were quantified at baseline and the end of the study. Protocols for induction of hyperammonaemia, EchoMRI, indirect calorimetry, and biochemical assays are described in Supplementary Methods. At the end of the study, noninvasive physiological measures were obtained, mice were euthanized, followed by the harvesting of organs, including muscle tissue (Figure S1A).

### 2.3 Statistical Analysis

The four groups of mice were stratified by intervention (UA/VWR) and treatment (AmAc/PBS). Quantitative data were expressed as mean ± standard deviation (SD), and individual data are shown in the figures. Comparisons of body weight, grip strength, activity (total, X‐, and Z‐axis), and metabolic parameters, including VO_2_, VCO_2_, respiratory exchange ratio (RER), and energy expenditure (EE), were performed pre‐ and post‐intervention, and changes with intervention were evaluated in mice stratified by treatment. Additional details are shown in Methods S1. Statistical tests applied to the data are described within the legends for each figure. Exact *p* values for interaction and main effects (when applicable) are shown in Table S2.

## 3 Results

### 3.1 Body Composition and Biochemical Changes With Hyperammonaemia Respond to VWR

**FIGURE 1:**
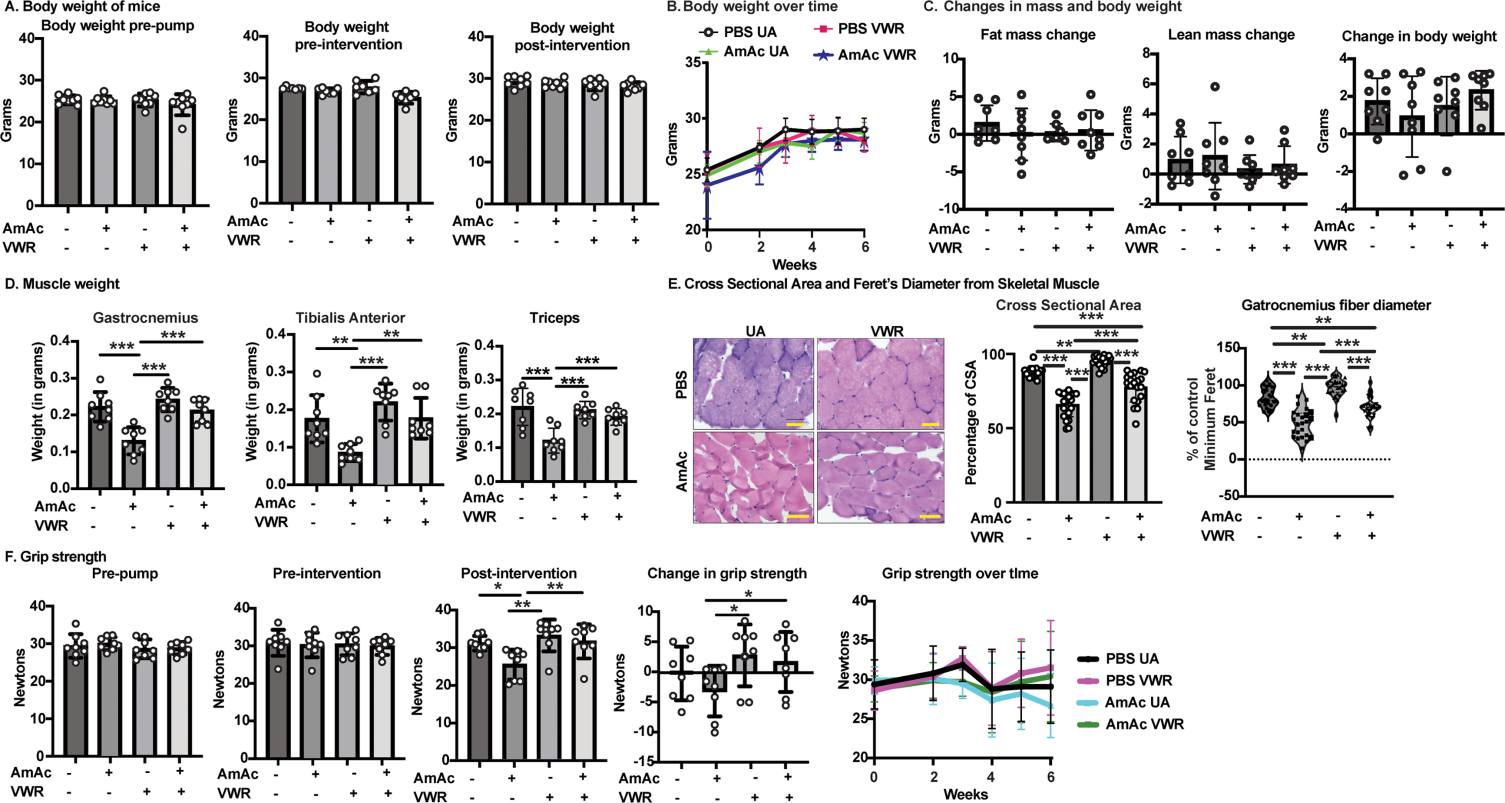
Body composition and functional responses to exercise altered by hyperammonaemia. Studies were performed in male C57BL6/J mice aged 8–10 weeks, treated with either sterile phosphate‐buffered saline (PBS, vehicle) or 2.5 mmol kg^−1^ day^−1^ ammonium acetate (AmAc) for 42 days. Two weeks postpump placement, mice were randomized to interventions (usual activity, UA, or voluntary wheel running, VWR) for 4 weeks. (A) Body weight before osmotic pump placement (pre‐pump), before intervention (2‐weeks post‐pump), and at 4 weeks post‐intervention. (B) Change in body weight over time. (C) Change in fat mass, lean mass, and overall body weight in all 4 groups of mice. (D) Gastrocnemius (Gastroc), tibialis anterior (TA), and triceps muscle weight post‐intervention. (E) Representative image and bar graph of cross‐sectional area and Feret's diameter for skeletal muscle frozen sections. (F) Grip strength before the installation of the osmotic pumps (pre‐pump), before intervention, post‐intervention, and change in grip strength between the pre‐ and post‐intervention. Data: mean ± SD. *Statistical analyses*: One‐sample Kolmogorov–Smirnov test for normality. (A) Data distribution was not normal; Kruskal–Wallis analysis and Dunn's multiple comparison post hoc analysis were used. (B) Data distribution was normal; two‐way ANOVA followed by Tukey's post hoc test was performed; (C–G) data distribution was normal; one‐way ANOVA followed by Tukey's post hoc test was performed. *p < 0.05; **p < 0.01; ***p < 0.001 *n* = 8 mice in each group.

Total body weight increased in all mice except the PBS‐VWR group post‐intervention versus pre‐intervention (Figure 1A,B; Figure S1B,C). However, there were no intergroup differences in the whole body, lean, and fat mass, and the proportion of whole body lean and fat mass to total body weight at any of the time points (Figure 1C; Figure S1B–D). Blood (*p* < 0.01) and skeletal muscle ammonia concentrations (*p* < 0.01) were higher in the AmAc compared with PBS mice with either UA or VWR, with the highest levels in AmAc‐VWR mice (Figure S1E). Plasma alanine aminotransferase (ALT), aspartate aminotransferase (AST), glucose, and insulin were not different between the four groups (Figure S1F; Table S3).

### 3.2 Voluntary Wheel Running Reverses ammonia‐Induced Skeletal Muscle Loss and Strength

Gastrocnemius, tibialis anterior, and triceps muscle weights were less with AmAc than PBS in the UA mice (*p* ≤ 0.01). Muscle weights were higher with VWR than UA in AmAc but not PBS mice (Figure 1D). Muscle weights in AmAc‐VWR mice were similar to those in the PBS groups (UA/VWR), suggesting that 4 weeks of VWR reversed hyperammonaemia‐induced muscle loss. Cross‐sectional area (CSA) and minimum Feret analyses of fibre diameter in muscle sections were consistent with our previous report [8] and showed AmAc‐UA mice had the lowest CSA (*p* ≤ 0.001) and fibre diameter (*p* ≤ 0.001) compared with other groups. Interestingly, VWR in AmAc‐treated mice partially rescued CSA and fibre diameter (*p* < 0.001 for both) (Figure 1E). The relative proportion of different fibre types showed fewer Type IIA fibres in the AmAc‐UA mice, which were reversed by VWR (Figure S1G). There were fewer Type IIB and more Type IIx fibres in PBS‐VWR compared with PBS‐UA mice. There was no difference in Type IIB or Type IIx fibres without or with exercise in the AmAc‐treated groups, or between the AmAc‐treated groups and the UA‐PBS group (Figure S1G).

Pre‐intervention (UA/VWR) grip strengths were similar in both treatment (AmAc and PBS) groups (*p* > 0.1). Post‐intervention grip strength in AmAc UA mice was lower than that in other groups (*p* < 0.05). Grip strength (pre‐ to post‐intervention) decreased in the AmAc‐UA group but increased in the VWR groups (*p* < 0.05 for both) (Figure 1F). There were no differences in grip strength between the AmAc‐VWR mice, PBS‐UA, or PBS‐VWR mice, which shows that VWR reversed AmAc‐induced decrease in grip strength. To mechanistically determine if VWR or AmAc altered the neuromuscular junction [14], we evaluated the expression of agrin protein, a muscle‐specific receptor tyrosine kinase activator that is essential for postnatal maintenance of neuromuscular synapses (Reference S4). No differences in agrin expression were noted in any of the groups (Figure S1H). Adipose tissue weight was lower with VWR than UA only in the AmAc mice (*p* < 0.05) with no differences in other organ weights (heart, liver, or kidney) between the four groups of mice (Figure S1I).

### 3.3 Hyperammonaemia Differentially Affects Activity in Mice

**FIGURE 2:**
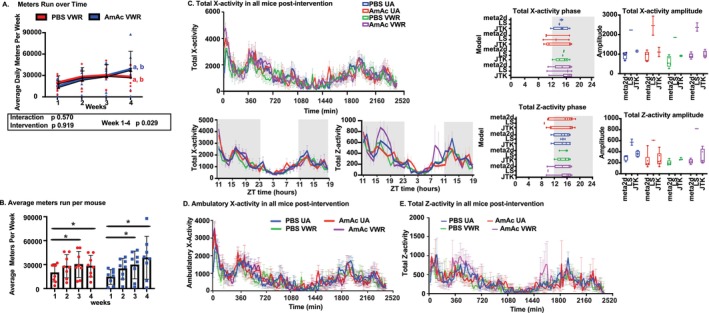
Locomotor activity during hyperammonaemia. Studies were performed in male C57BL6/J mice aged 8–10 weeks, treated with either sterile phosphate‐buffered saline (PBS, vehicle) or 2.5 mmol kg^−1^ day^−1^ ammonium acetate (AmAc) for 42 days. Two weeks postpump placement, mice were randomized to interventions: usual activity (UA) or voluntary wheel running (VWR) for 4 weeks with daily wheel rotation measurements. Post‐intervention activity and zeitgeber time (ZT) were measured in a Comprehensive Lab Animal Monitoring System (CLAMS) metabolic cage system for 48 h of data collection. Additionally, the amplitude and phase of the circadian peaks were recorded. (A) Weekly rotations over 4 weeks. (B) Average wheel rotations over 4 weeks. (C) Mean total X‐activity, amplitude, and phase of circadian patterns. (D) Mean ambulatory X‐activity. (E) Mean total Z‐activity, amplitude, and phase of circadian patterns. Data: mean ± SD. *Statistical analyses*: The Shapiro–Wilk test for normality was performed. (A–E) Because data were not normally distributed, the aligned rank transform (ART) for nonparametric two‐way ANOVA analysis was performed to determine the independent effect of treatment (PBS vs. AmAc) and time (panels A and B) and the independent effects of treatment and intervention (panels C–E) and the interaction effect between independent variables. Post hoc analysis was performed using Dunn's test with Benjamini–Hochberg post hoc correction. For circadian data in panels C and E, (C) line graphs and amplitude were not normally distributed. Hence, the Kruskal–Wallis test with Dunn's multiple comparison test for post hoc analysis was used. Phase data were normally distributed. One‐way ANOVA followed by Tukey's multiple comparisons was used. (E) Line graphs and phase were not normally distributed. Hence, the Kruskal–Wallis test with Dunn's multiple comparison test for post hoc analysis was used. Amplitude data were normally distributed. One‐way ANOVA followed by Tukey's multiple comparisons was used. *p < 0.05; **p < 0.01; ***p < 0.001 unless stated within the figure. Panel A “a” *p* < 0.05 between weeks 1 and 3, “b” *p* < 0.05 between weeks 1 and 4. *N* = 8 mice in each group. Comparisons of average meters run by week between treatments (PBS vs. AmAc) showed no differences (*p* > 0.05).

Consistent with published data [13], wheel rotations and distance run increased over the first 2 weeks and then plateaued in both PBS‐VWR and AmAc‐VWR mice (Figure 2A,B). There was a significant effect of time (*p* = 0.03), but no significant difference between treatment groups (PBS vs. AmAc, *p* = 0.92). Running distance increased from week 1 to week 3 (*p* = 0.05) and week 4 (*p* = 0.04) (Figure 2A,B; Figure S2A). Pre‐intervention or post‐intervention (UA or VWR) total X‐activity (any movement across the length of the cage) and ambulatory X‐activity (ambulatory movement across the length of the cage) were not different between the groups and did not change before and after the intervention period (Figure 2C,D; Figures S2B, S3, and S4). These data show that X‐activity does not change with either AmAc treatment or VWR intervention. Average Z‐activity (jumping movements) pre‐intervention was greater (*p* < 0.01) in AmAc‐UA mice compared with all other groups, but post‐intervention average Z‐activity was not different between the 4 groups of mice (Figure S5A–F). Even though post‐intervention average Z‐activity was not different with treatment or intervention (Figure 2E), temporal differences were noted during specific times when AmAc‐VWR mice had higher (*p* < 0.01 or less) total Z‐activity than PBS‐VWR. From 324 to 720 min, the average Z‐activity between AmAc‐VWR and PBS‐VWR was not different (*p* = 0.07), while total Z‐activity (during the dark cycle) was higher (*p* = 0.01) in AmAc‐VWR than in PBS‐VWR (Figure S5G). Interestingly, both total Z‐activity and the average Z‐activity of all mice were greater in AmAc‐VWR mice (*p* < 0.05) from 1440 to 1800 min (dark cycle) only. In summary, after the 4 weeks of intervention (UA/VWR), AmAc‐VWR mice had more wheel rotations (primarily during the dark cycle, with some extension to the light cycle also) and greater total and average Z‐activity than PBS‐VWR mice, while X‐activity (total or ambulatory) was similar in these two groups of mice. Because X‐ and Z‐activities reflect different types of movements, we determined if these were related. Total X‐activity correlated with ambulatory X‐activity (*p* < 0.0001) and total Z‐activity (*p* < 0.0001), while ambulatory X‐activity was correlated with total Z‐activity (*p* < 0.0001) in all groups of mice (Figure S6A–D). We then determined if ammonia altered VWR‐induced activity patterns and found that the number of wheel rotations correlated with total X‐activity (*p* = 0.01) and ambulatory X‐activity (*p* = 0.01) in the PBS‐VWR but not AmAc‐VWR mice (Figure S6E,F).

### 3.4 Circadian Patterns Were Not Different Among the Experimental Groups

**FIGURE 3:**
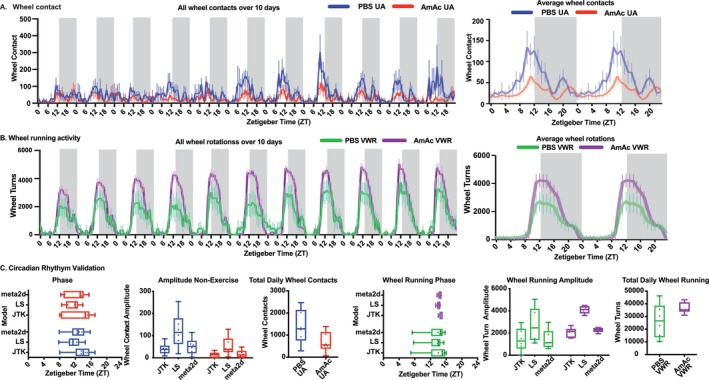
Wheel contact activity during hyperammonaemia. Studies were performed in male C57BL6/J mice aged 8–10 weeks, treated with either sterile phosphate‐buffered saline (PBS, vehicle) or 2.5 mmol kg^−1^ day^−1^ ammonium acetate (AmAc) for 42 days. Two weeks postpump placement, mice were randomized to interventions: usual activity (UA) or voluntary wheel running (VWR) for 4 weeks. The number of wheel rotations and the zeitgeber time (ZT) of rotations were measured over the 28‐day intervention period. (A,B) Hourly wheel contacts and wheel rotations (*n* = 6 in each group) for 10 of the 28 days of the intervention period (left) with double‐plotted averages for six mice of 40 h of contact measurement. (C) Phase and amplitude of the peaks of activity (Blue PBS‐UA, Red AmAc‐UA, Green PBS‐VWR, Purple AmAc‐VWR). For panels A and B, the clear region is light, and the grey region is a dark cycle. Data: mean ± SD. *Statistical analyses*: The one‐sample Kolmogorov–Smirnov test was used to determine if data distribution was normal. Panels A, B: Data were not normally distributed; hence the two‐sample Kolmogorov–Smirnov test was used for comparisons. For panel C, UA phase and amplitude, and for VWR mice, phase data were normally distributed, and the unpaired Student's *t* test was used. Data for the line graph for UA and VWR mice and amplitude for VWR mice were not normally distributed, and the two‐sample Kolmogorov–Smirnov test was used for comparisons. *p < 0.05; **p < 0.01; ***p < 0.001 unless stated within the figure. *N* = 6 mice in each group (in two mice, continuous data acquisition was interrupted due to technical issues and were not used).

Circadian patterns for both wheel rotation and activity data (from the metabolic cages) showed that in the UA‐mice, wheel contacts peaked at zeitgeber (ZT)12, the start of the dark cycle, with more wheel contacts (*p* < 0.05) in PBS‐UA than in AmAc‐UA mice (Figure 3A; Table 1). Peak wheel running (and distance run) in PBS‐VWR mice was at ZT13 and at ZT14 in AmAc‐VWR mice (Figure 3B; Figure S7; Table 1). The amplitude of the peaks of wheel contact/rotation was not affected by treatment (PBS/AmAc) within the intervention (UA/VWR) groups (Figure 3C). During the dark cycle (10–18 h), on certain days (3–5, 10, 11), AmAc‐VWR mice had higher wheel rotations and distance run (*p* < 0.05) than the PBS‐VWR mice (Figures S8A,B and S9A,B). In contrast, the AmAc‐VWR mice had significantly (*p* < 0.05) fewer average wheel rotations (vs. PBS VWR) during the light cycle (0–9 h; on days 4–7 and 10) (Figures S8C and S9C). Even though the average number of wheel rotations (and distance run) was not different in the AmAc‐VWR and PBS‐VWR mice across both the dark and light cycles, wheel rotations (and distance run) were significantly greater during the dark cycle (*p* < 0.001) but not the light cycle (*p* = 0.44) in the AmAc‐VWR than PBS‐VWR mice (Figure S8D and S9D).

**TABLE 1:** Circadian parameters of wheel contacts and wheel running activity.

	JTK_adjphase	JTK STDEV	JTK SEM	LS_adjphase	LS STDEV	LS SEM	meta2d phase	meta2d STDEV	meta2D SEM
M PBS E1	13.8	3.4	1.4	13.2	2.7	1.1	13.5	3.0	1.2
M AmAc E1	14.6	0.4	0.2	14.0	0.4	0.2	14.3	0.4	0.2
M PBS 1	13.6	3.0	1.2	11.4	2.2	0.9	12.5	2.6	1.1
M AmAc 1	12.9	3.7	1.5	11.4	1.9	0.8	12.2	2.8	1.1

	JTK amplitude	JTK STDEV	JTK SEM	LS adjphase	LS STDEV	LS SEM	meta2d AMP	meta2d STDEV	meta2d SEM
M PBS E1	1420.4	1031.7	421.2	2738.9	1479.2	603.9	1416.6	873.5	356.6
M AmAc E1	2053.7	421.0	171.9	4088.5	341.2	139.3	2266.1	201.0	82.1
M PBS 1	23.1	17.2	7.0	122.2	79.0	32.2	52.1	37.7	15.4
M AmAc 1	13.7	12.1	4.9	52.8	42.9	17.5	17.3	17.4	7.1

Post‐intervention circadian patterns were not different between groups (Figure S10A–C). The phase and amplitude of X‐ or Z‐activity were also not different between any of the groups (Figure 2C–E). The total/ambulatory‐X and Z‐activity were higher (*p* ≤ 0.01 for all comparisons) during the dark cycle as compared with the light cycle in all four groups of mice (Figure S10A–C).

### 3.5 VWR Reversed Hyperammonaemia‐Induced Perturbations in Whole‐Body Metabolic Parameters

**FIGURE 4:**
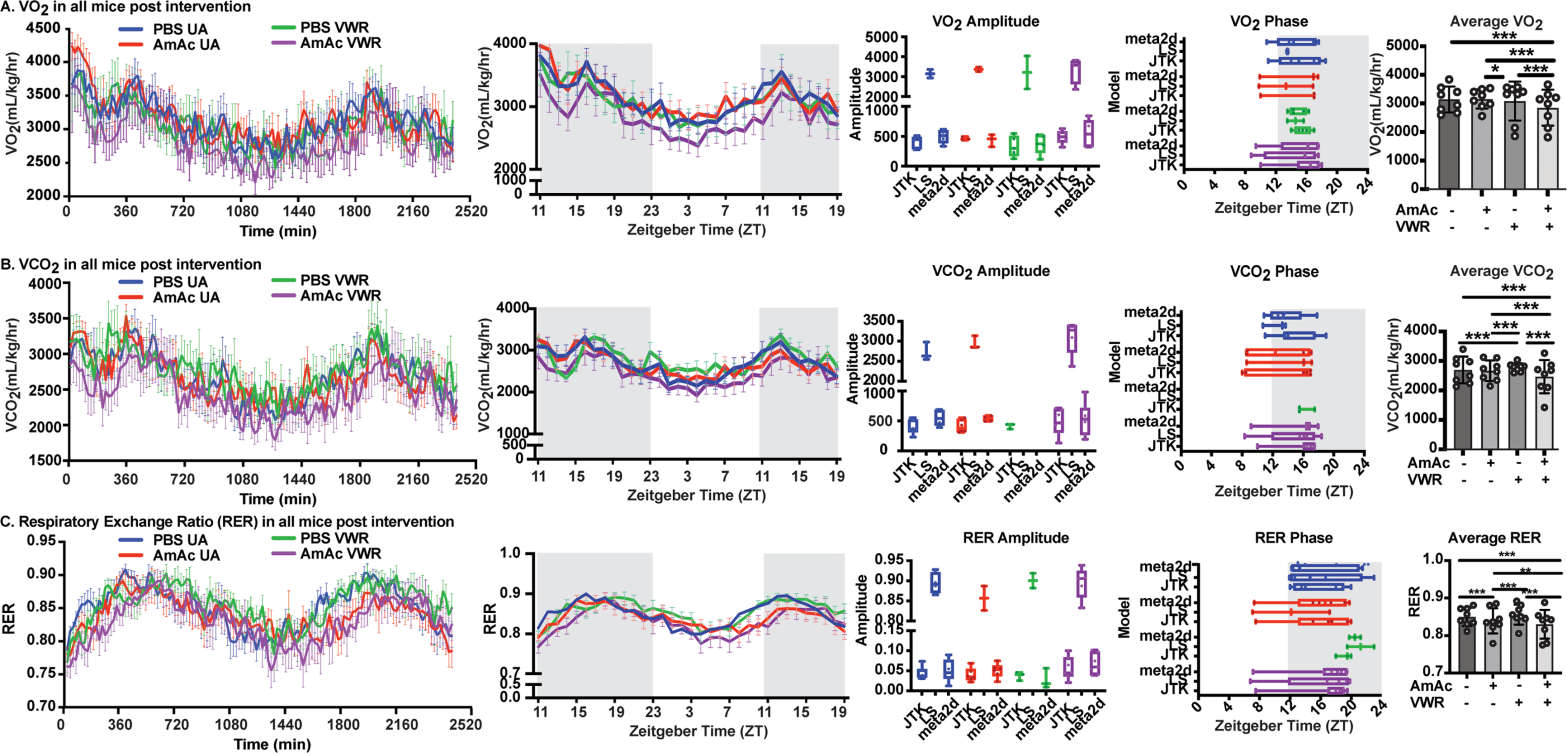
Whole body metabolic responses to exercise during hyperammonaemia. Studies were performed in male C57BL6/J mice aged 8–10 weeks, treated with either sterile phosphate‐buffered saline (PBS, vehicle) or 2.5 mmol kg^−1^ day^−1^ ammonium acetate (AmAc) for 42 days. Two weeks postpump placement, mice were randomized to interventions: usual activity (UA) or voluntary wheel running (VWR) for 4 weeks. Post‐intervention activity was measured in a Comprehensive Lab Animal Monitoring System (CLAMS) metabolic cage system for 48 h. Prior to the start of the exercise intervention and after 4 weeks of intervention, indirect calorimetry was performed for 48 h. The zeitgeber time (ZT) was also recorded over the 48 h of data collection. Additionally, the amplitude and phase of the peaks of the metabolic data were recorded. (A,B) Volume of oxygen inhaled (VO_2_) and volume of carbon dioxide exhaled (VCO_2_) over time and average values with line/bar graphs showing the Amplitude and Phase circadian patterns of VO_2_ (A) and VCO_2_ (B). (C) Respiratory exchange ratio (RER) (VO_2_/VCO_2_) over time and average values with line/bar graphs showing the Amplitude and Phase circadian patterns of RER. Data: mean ± SD. *Statistical analyses*: (A–C) Because data distribution was not normal (Shapiro–Wilk test) for metabolic measures (VO_2_, VCO_2_, and RER), the aligned rank transform (ART) for nonparametric two‐way ANOVA analysis was used to determine the independent effect of treatment and intervention effects, and if an interaction effect existed between treatment and time. Post hoc analysis was performed using Dunn's test with Benjamini–Hochberg post hoc correction. For circadian data in panels A–C, data were analysed with one‐way ANOVA, followed by Tukey's multiple comparison test, except for panels A and B line and amplitude data, which were not normally distributed, the Kruskal–Wallis test followed by Dunn's multiple comparisons post hoc analysis was used. *p < 0.05; **p < 0.01; ***p < 0.001 unless stated within the figure. *N* = 8 mice in each group.

Whole‐body metabolic measures, including substrate utilization patterns (VO_2_, VCO_2_, RER, EE), are altered in patients with cirrhosis in whom hyperammonaemia is a consistent abnormality [15, 16]. Pre‐intervention VO_2_ was lower in AmAc‐treated versus PBS‐treated mice, irrespective of intervention (UA and VWR), while post‐intervention VO_2_ was lower in the AmAc‐VWR mice as compared with the other groups (*p* < 0.001) (Figure 4A; Figure 11A–C). There was no difference between pre‐intervention and post‐intervention VO_2_ in any of the groups (Figure 11C–F). Pre‐intervention and post‐intervention VCO_2_ was lower in the AmAc‐VWR mice as compared with all other groups (*p* < 0.001), while in the PBS‐treated mice, VWR intervention resulted in greater VCO_2_ (Figure 4B; Figure S12A,B). There was no difference in VCO_2_ pre‐ to post‐intervention in any of the groups (Figure S12C–F). Pre‐intervention and post‐intervention RER was lower in both AmAc‐treated groups than PBS‐treated mice with the same intervention (*p* < 0.01) but not different between PBS‐UA and PBS‐VWR mice (Figure 4C; Figure S13A,B). Pre‐intervention to post‐intervention RER was not different in any of the groups (Figure S13C–F). Circadian patterns were observed in VO_2_, VCO_2_, and RER in all groups of mice, but there were no differences in circadian patterns in the phase and amplitude of the peaks between any of the groups (Figure 4). VO_2_ and RER were higher in the dark cycle (*p* ≤ 0.05) than the light cycle in all four groups, while VCO_2_ was greater in the dark cycle in only PBS‐UA and AmAc‐UA/VWR mice, but not different in the PBS‐VWR mice (*p* > 0.05) (Figure S14A–C). VO_2_ and RER were not different between the four groups of mice in the light or dark cycle, but VCO_2_ in AmAc‐VWR mice was less (*p* = 0.03) than in PBS‐VWR during the light cycle only (Figure S14A–C). Pre‐intervention and post‐intervention energy expenditure (EE) was lower (*p* < 0.02) in both groups of AmAc‐treated (UA/VWR) compared with PBS‐treated (UA/VWR) mice (Figures S15 and S16A,B). EE was greater in the PBS‐VWR mice post‐intervention compared with pre‐intervention (*p* = 0.04), but not different between the other groups (Figure S17A–D). Because muscle mass, a major contributor to EE, differed between groups, we did not normalize EE data to lean or whole‐body weight as has also been suggested by others (Reference S5). Circadian patterns of EE were consistent with other metabolic measures, with no differences between the four groups of mice (Figure S18A). There was also no difference in the amplitude of the phase of the circadian peaks of EE. We compared EE during the light and dark cycles. All four groups of mice had greater EE in the dark cycle than the light cycle (*p* ≤ 0.05), and there was no difference in EE in either the light or dark cycle between any of the groups (Figure S18B). There were also no differences between groups in total food intake, lowest/highest/average amount of food taken, or the number of food withdrawal events (Figure S19A–G).

### 3.6 VWR Restores Ammonia‐Induced Mitochondrial Oxidative Dysfunction

**FIGURE 5:**
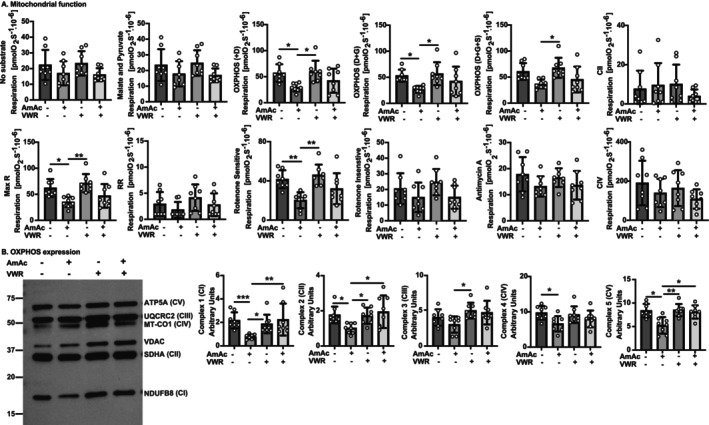
Mitochondrial functional responses to exercise during hyperammonaemia. Studies were performed in male C57BL6/J mice aged 8–10 weeks, treated with either sterile phosphate buffered saline (PBS, vehicle) or 2.5 mmol kg^−1^ day^−1^ ammonium acetate (AmAc) for 42 days. Two weeks postpump placement, mice were randomized to interventions: usual activity (UA) or voluntary wheel running (VWR) for 4 weeks. (A) Mitochondrial oxygen consumption was measured by high‐resolution respirometry in the gastrocnemius muscle. Substrate uncoupler inhibitor titration protocol with sequential addition of electron transport chain (ETC) complex substrates, malate (M), pyruvate (P), ADP (D), glutamate (G) and succinate (S), protonophore carbonyl cyanide‐4‐(trifluoromethoxy) phenylhydrazone (FCCP), complex I inhibitor rotenone (R), antimycin‐A, and sodium azide. Proton leak, oxidative phosphorylation (OXPHOS) maximum respiration (Max. R), reserve respiratory capacity (RR), rotenone (Rot.)‐sensitive and ‐insensitive respiration, and complex IV function were measured. (B) Representative immunoblots and densitometry of critical components of oxidative phosphorylation (OXPHOS): complex I, NADH: ubiquinone oxidoreductase subunit B8 (NDUFB8), complex II, succinate dehydrogenase subunit A (SDHA), complex III, cytochrome b‐c1 complex subunit 2 (UQCRC2) complex IV, mitochondrially encoded cytochrome C oxidase 1 (MT‐CO1), complex V, ATP synthase lipid binding protein (ATP5A). Data: mean ± SD. *Statistical analyses*: The one‐sample Kolmogorov–Smirnov test was performed to evaluate whether the data distribution was normal. One‐way ANOVA followed by Tukey's post hoc analysis for normally distributed data (panel A: ADP, rotenone‐sensitive, antimycin A, complex IV respiration; panel B: Mitochondrial mass; panel C: complexes III, IV, V). Other data were not normally distributed. Hence, a Kruskal–Wallis test followed by Dunn's multiple comparison post hoc test was used. *N* = 8 mice for each group. *p < 0.05; **p < 0.01; ***p < 0.001.

Mitochondrial oxidative function and metabolic responses in skeletal muscle are impaired by hyperammonaemia [7, 9, 10]. Others have reported improved muscle mitochondrial oxidative function with exercise [17]. We, therefore, evaluated muscle mitochondrial function in response to hyperammonaemia and VWR. Responses to complex I substrates (ADP; *p* = 0.02; glutamate *p* = 0.05), maximum respiration (*p* = 0.02), and the rotenone‐sensitive response were less (*p* = 0.04) in the AmAc‐UA than in the PBS‐UA mice (Figure 5A). These data are consistent with our previous reports that hyperammonaemia causes skeletal muscle mitochondrial oxidative dysfunction [7, 9]. There were no differences in reserve respiratory capacity, rotenone‐insensitive, CII, or CIV respiration between groups. There was no change in mitochondrial mass in any of the groups as measured by the expression of voltage‐dependent anion channel (VDAC) or citrate synthase (CS) proteins (Figure S20). These data suggest that improved mitochondrial efficiency rather than increased mitochondrial mass contributes to higher muscle mitochondrial responses with VWR. We then determined the expression of proteins of critical electron transport chain (ETC) complex components (C) without/with AmAc post‐intervention. The expression of key components of CI (NADH: ubiquinone oxidoreductase subunit B8; NDUFB8), CIV (mitochondrially encoded cytochrome C oxidase 1; MT‐CO1), and CV (ATP synthase lipid‐binding protein; ATP5A) was lower in the AmAc‐UA mice compared with the PBS‐UA mice (*p* < 0.05). VWR reversed these changes in AmAc mice in CI and CV complexes (*p* < 0.05) but not CIV. The expression of a key component of CII (succinate dehydrogenase subunit A; SDHA) was lower (*p* < 0.02) in the AmAc‐UA compared with PBS‐UA and in the AmAc‐VWR group (Figure 5C). These data show that VWR reverses ammonia‐induced impaired mitochondrial oxidative function and expression of critical ETC complex components.

### 3.7 VWR Reverses Dysregulated Protein Homeostasis of Hyperammonaemia

**FIGURE 6:**
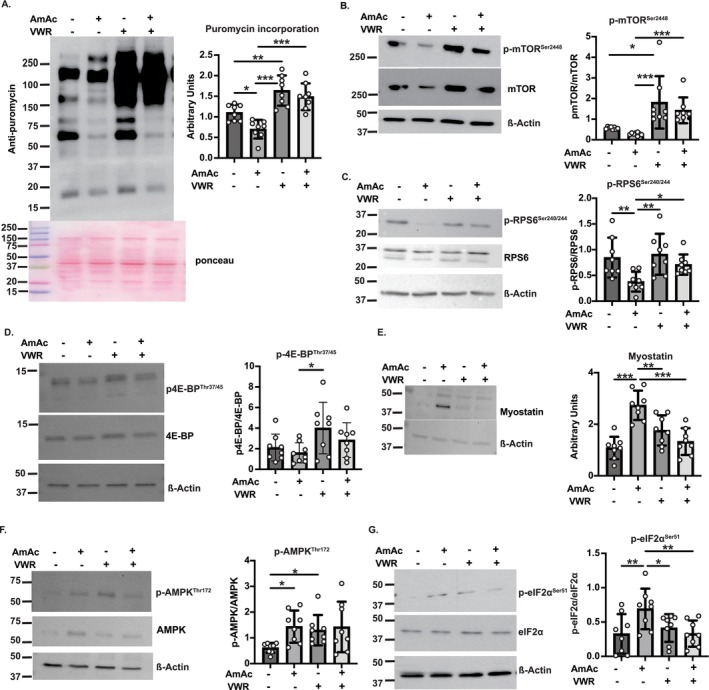
Muscle protein synthesis and regulatory signalling molecules with exercise during hyperammonaemia. Studies were performed in male C57BL6/J mice aged 8–10 weeks, treated with either sterile phosphate‐buffered saline (PBS, vehicle) or 2.5 mmol kg^−1^ day^−1^ ammonium acetate (AmAc) for 42 days. Two weeks postpump placement, mice were randomized to interventions: usual activity (UA) or voluntary wheel running (VWR) for 4 weeks. Representative immunoblots and densitometry for the following: (A) Puromycin incorporation as a measure of protein synthesis. (B) Phosphorylated mammalian target of rapamycin (mTOR^Ser2448^), total mTOR. (C) Phosphorylated ribosomal S6 protein (RPS6^Ser240/244^), total ribosomal S6 protein. (D) Phosphorylated eukaryotic initiation 4‐binding protein (4E‐BP^Thr37/45^), total 4E‐BP. (E) Myostatin. (F) Phosphorylated adenosine monophosphate‐activated protein kinase (AMPK^Thr172^), total AMPK. (G) Phosphorylated eukaryotic initiation factor‐2 alpha (eif2α^Ser52^), total eif2α. Loading controls included β‐actin from the same lysate in the same membrane as the proteins of interest is shown. Data: mean ± SD. *Statistical analyses*: A one‐sample Kolmogorov–Smirnov test was performed to evaluate if data distribution was normal. (A,C,D,E,G) One‐way ANOVA followed by Fisher's least significant difference for post hoc analysis. (B,F) Data were not normally distributed. Hence, a Kruskal–Wallis test was performed, followed by Dunn's multiple comparison post hoc test. *N* = 8 in each group. *p < 0.05; **p < 0.01; ***p < 0.001.

Hyperammonaemia results in decreased skeletal muscle protein synthesis and mTORC1 signalling, while autophagy and phosphorylation of eIF2α are increased [5, 6, 8, 9, 18, 19]. Because exercise increases muscle ammoniagenesis [11] (Reference S2), we evaluated the effects of VWR on hyperammonaemia‐induced dysregulated protein homeostasis. Consistent with our prior reports [20], global skeletal muscle protein synthesis in the AmAc‐UA mice was less than that in the PBS‐UA mice (*p* = 0.01) (Figure 6A). Protein synthesis was higher in PBS‐VWR than in PBS‐UA (*p* = 0.001) and in AmAc‐VWR than AmAc‐UA mice (*p* < 0.001), with no difference between PBS‐VWR and AmAc‐VWR mice (Figure 6A). These data show that VWR reverses lower muscle protein synthesis during hyperammonaemia.

Skeletal muscle protein homeostasis is regulated by a number of molecular mediators that include mTORC1 signalling, which increases protein synthesis, and MSTN, which inhibits muscle protein synthesis and muscle mass [5, 21]. Hyperammonaemia and exercise alter muscle mTORC1 signalling and MSTN expression [5, 22], but the interaction of ammonia and exercise on signalling molecules is not known. We noted more phosphorylated (p‐mTOR)^Ser248^ with VWR than UA in both treatment groups (PBS‐VWR vs. PBS‐UA, *p* = 0.05; AmAc‐VWR vs. AmAc‐UA, *p* = 0.001), with no difference between PBS‐VWR and AmAc‐VWR, suggesting an increase in protein synthesis with VWR (Figure 6B). Even though muscle p‐mTOR^Ser248^ in PBS‐UA and PBS‐AmAc was similar, expression of phosphorylated ribosomal S6 (p‐RPS6)^Ser240/244^, an mTORC1 signalling response, was less (*p* < 0.01) in AmAc‐UA than PBS‐UA but was reversed (*p* = 0.04) in AmAc‐VWR mice (Figure 6C). These data are consistent with previous reports that hyperammonaemia impairs mTORC1 signalling [8], which was reversed by VWR. Another mTORC1 target, phosphorylated 4E‐binding protein 1 (p‐4EBP1)^Thr37/45^ was not different between the two groups of UA mice or in response to VWR within each group (Figure 6D). Such a discord between different downstream targets of mTORC1 has been suggested to be differential sensitivity of these targets in a context‐specific manner (Reference S6). Expression of MSTN protein, a negative regulator of muscle mass that is upregulated during hyperammonaemia [6, 8], was higher in gastrocnemius muscle from AmAc‐UA mice than PBS‐UA mice (*p* < 0.001). AmAc‐VWR had lower expression of MSTN than AmAc UA mice, providing a mechanistic explanation for the beneficial effects of VWR on muscle mass during hyperammonaemia (Figure 6E). Expression of phosphorylated AMP‐activated protein kinase (p‐AMPK)^Thr172^, which inhibits mTOR phosphorylation and signalling, impairs protein synthesis and increases autophagy, was higher (*p* < 0.05) in AmAc‐UA versus PBS‐UA mice (Figure 6F), which is consistent with prior reports [8]. Higher p‐AMPK was noted in PBS‐VWR versus PBS‐UA mice (*p* = 0.03). Consistent with previous reports, phosphorylation of eukaryotic Initiation Factor 2 (p‐eif2α^Ser51^), which inhibits mRNA translation during hyperammonaemia [18], was higher in muscle from AmAc‐UA mice than PBS‐UA (*p* = 0.001) (Figure 6G); however, VWR did not alter phosphorylation of eif2α compared with UA in PBS mice.

**FIGURE 7:**
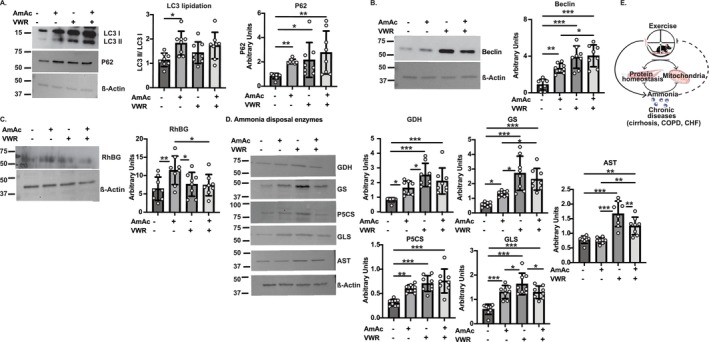
Skeletal muscle autophagy markers and ammonia responses to exercise during hyperammonaemia. Studies were performed in male C57BL6/J mice aged 8–10 weeks treated with either sterile phosphate‐buffered saline (PBS, vehicle) or 2.5 mmol kg^−1^ day^−1^ ammonium acetate (AmAc) for 42 days. Two weeks postpump placement, mice were randomized to interventions: usual activity (UA) or voluntary wheel running (VWR) for 4 weeks. Representative immunoblots and densitometry for the following: (A) Microtubule‐associated light chain I and II (LC3 I and II), P62; (B) Beclin1; (C) Rhesus glycoprotein B (RhBG). (D) Ammonia disposal enzymes: glutamate dehydrogenase (GDH), glutamine synthase (GS), pyrroline‐5‐carboxylate synthase (P5CS), glutaminase‐2 (GLS), and aspartate aminotransferase (AST). (E) Graphical abstract. Interaction between VWR and hyperammonaemia. CHF: congestive heart failure; COPD: chronic obstructive pulmonary disease. β‐Actin was used as a loading control for all blots. (A,C) Blots for P62 and myostatin were run using the same lysate and on the same membrane; loading control β‐actin is for p62 and myostatin. (B) Loading control β‐actin for Beclin‐1. (F) β‐actin from RhBG. (G) Loading control representative β‐actin is for ammonia disposal enzymes (GS and P5CS), were run on the same membrane. Data: mean ± SD. *Statistical analyses*: A one‐way Kolmogorov–Smirnov test for normality of distribution was performed. One‐way ANOVA followed by Fisher's least significant difference post hoc analysis for normally distributed data, except for panels A (P62) and D (GS), which did not satisfy normality tests. Hence, the nonparametric Kruskal–Wallis test was performed, followed by Dunn's multiple comparison post hoc test. *n* = 8 mice in each group. All experiments in mice were done on n = 8 mice for each group. *p < 0.05; **p < 0.01; ***p < 0.001.

Both hyperammonaemia and exercise increase muscle autophagy [19, 23], but it is not known whether hyperammonaemia alters exercise‐induced autophagy or vice versa. Consistent with prior reports, the LC3‐II to LC3‐I ratio was higher in AmAc‐UA mice than in PBS‐UA mice (*p* < 0.01), but the ratio in VWR mice was not different from either UA group, suggesting VWR may increase autophagy to a lesser extent than AmAc alone (Figure 7A). The expression of P62 and Beclin was higher in the gastrocnemius muscle from AmAc‐UA, PBS‐VWR, and AmAc‐VWR mice compared with the PBS‐UA mice (*p* < 0.05) but was not different between the two VWR mouse groups (Figure 7A,B). These data show that AmAc increases autophagy, as does VWR, but exercise may not increase AmAc‐induced autophagy further.

### 3.8 Hyperammonaemia and VWR Increased Ammonia Uptake and Disposal Enzymes

Because AmAc and VWR increased blood and muscle ammonia concentrations, we evaluated the expression of the regulated ammonia transporter, Rhesus family B Glycoprotein (RHBG) [20]. Expression of RhBG was higher in AmAc‐UA mice than in PBS‐UA mice but did not change with VWR irrespective of treatment (Figure 7C).

Despite increased circulating and muscle ammonia concentrations, beneficial responses to VWR were noted. We therefore evaluated ammonia disposal pathway enzymes that may help restore metabolic homeostasis [7, 21]. Expression of key components of skeletal muscle ammonia metabolism, including glutamate dehydrogenase (GDH), glutamine synthase (GS), pyrrolidine‐5‐carboxylate synthase (P5CS), and glutaminase (GLS), was higher in AmAc‐UA compared with PBS‐UA (*p* < 0.05). Expression of the ammonia disposal enzymes was higher in the PBS‐VWR mice than in the PBS‐UA mice (*p* < 0.001). However, only GS expression was increased in AmAc‐VWR mice compared with AmAc‐UA, with no differences between the two groups in GDH, P5CS, or GLS expression. There was no difference in the expression of any of the ammonia disposal enzymes, other than GLS, between PBS‐VWR and AmAc‐VWR animals (Figure 7D). Expression of AST in PBS‐UA and AmAc‐UA mice was not different; however, VWR resulted in higher muscle AST levels in both PBS and AmAc mice as compared with either UA group (*p* < 0.01). These data show that hyperammonaemia increases the expression of ammonia transporter RhBG and ammonia disposal enzymes in skeletal muscle from UA mice. In contrast, VWR increases the expression of ammonia disposal enzymes in PBS‐treated mice; however, no further increase was observed in AmAc‐VWR mice, suggesting that higher RhBG expression is an adaptive response to hyperammonaemia, with a reduction to baseline (PBS‐UA) with VWR.

### 3.9 Correlation With Running Distance

We then correlated running distance with other outcomes of interest (physiological, phenotype, molecular) that improved in AmAc‐VWR. Because the distance run is believed to contribute to the muscle responses to exercise [24], we generated a correlation matrix for running distance as well as other outcome measures. In the PBS‐VWR mice, there was no significant correlation of running distance with other outcome measures (Figure S21A). Expression of phosphorylated ribosomal S6 protein positively correlated with AST (*p* = 0.03) in the PBS‐VWR mice, but there were no other significant correlations. In contrast, in AmAc‐VWR mice, running distance positively correlated with gastrocnemius muscle weight (*p* = 0.03), grip strength (*p* = 0.05), ETC complex I expression (*p* < 0.001), muscle protein synthesis (*p* < 0.01), and phospho‐mTOR expression (*p* < 0.01) (Figure S21B).

A comprehensive statistical summary table is included (Table S4), which provides a detailed description of the following: experimental questions, findings from each experiment performed, experimental variables, mean values and standard deviations, number of biological replicates, exact *p* values, and the figure or table where the data is shown/cited, units, groups compared, statistical test applied, experimental factors, and comments for each data shown in this manuscript.

## 4 Discussion

Endurance exercise improves skeletal muscle functional responses, which have been mechanistically studied in healthy humans and preclinical models [22]. Increased muscle ammoniagenesis, a physiological response to endurance exercise, contributes to fatigue and impairs exercise capacity [25]. Exercise responses during hyperammonaemia, a consistent finding in cirrhosis and other diseases, are not well studied. We show that hyperammonaemia causes skeletal muscle loss, impaired mitochondrial oxidative function, and mTORC1 signalling, with less protein synthesis. Interestingly, VWR partially reversed these perturbations, even though muscle ammoniagenesis was increased. Plasma and muscle ammonia increased with voluntary exercise despite greater expression of nonureagenic ammonia disposal enzymes in skeletal muscle. We also show that hyperammonaemia does not decrease voluntary activity in mice.

Endurance exercise decreases body weight and fat mass, with increased whole‐body lean mass [26]. However, the responses to VWR, a translationally relevant model for endurance exercise in humans, are not standardized [13]. Our studies in 8‐ to 10‐week‐old mice showed no differences in whole body weight, lean body mass, or fat mass between the groups before or after VWR, which is consistent with published data in mice of similar age and duration of intervention [27]. However, responses are different in older mice or those with long‐term (22‐week, 52‐week) intervention [28] (Reference S7), suggesting that age, sex, and duration of VWR impact body composition. Reduced muscle fibre cross‐sectional area/diameter in AmAc‐UA compared with other groups of mice is consistent with our findings of lower muscle mass and grip strength during hyperammonaemia, and is similar to our previous reports that ammonia causes sarcopenia [6, 8]. Even though Type I fibres did not change with either treatment or intervention, Type IIA fibres, with higher mitochondrial content, decreased with hyperammonaemia during UA but were reversed by VWR. In contrast, Type IIB fibres were less abundant during VWR in PBS but not in AmAc mice. These fibre type transitions may potentially be adaptive responses, as suggested by others [29], and may be a response to ammonia‐induced mitochondrial oxidative dysfunction reported earlier [9, 10], but need to be evaluated in the future. We also noted that VWR reversed AmAc‐induced sarcopenia and grip strength. Together, these data show that VWR improves muscle mass and grip strength during hyperammonaemia.

In the present study, measures of physical activity (i.e., average number of wheel rotations) during VWR were similar to those reported by others [13]. Interestingly, during the dark cycle, AmAc‐VWR mice had more wheel rotations than PBS‐VWR. However, during the light cycle, there were significantly fewer wheel rotations, particularly on specific days, suggesting periods of little to no activity in the AmAc‐VWR mice, which may be due to ammonia‐induced altered circadian patterns as reported by others [30]. In contrast, PBS‐VWR mice had fewer periods of little/no activity. Both exercise and muscle mass positively impact whole‐body metabolic measures, including VO_2_, VCO_2_, and RER [31]. Conversely, sarcopenia due to hyperammonaemia in cirrhosis is associated with less VO_2_ and RER [15]. Consistent with these reports, pre‐intervention AmAc mice had lower VO_2_ and RER than PBS mice. Even though VO_2_ max was not measured because it is challenging to precisely measure these in small animals due to the need for forced exercise [32], which causes significant stress and can affect molecular/metabolic responses (Reference S8). Our data suggest that endurance capacity is lower during hyperammonaemia, and VWR does not reverse the AmAc‐induced reduction in VO_2_.

Despite decreased muscle strength with hyperammonaemia [4], AmAc‐VWR mice had greater wheel running activity, and the onset of activity was delayed by 1ZT hour compared with PBS‐VWR mice. These data differ from reports by others, which indicate that mice fed a hyperammonemic diet for 2 months exhibited lower daily activity compared with their control counterparts, and that the time of start of activity advanced by 1 h during a normal light–dark cycle [30]. Potential explanations for the differences between our studies and those reported earlier include the impact of rapid first‐pass hepatic metabolism of ammonia in the oral model and fluctuations in blood ammonia levels that occur with oral administration. The previous study did not aim to evaluate the impact of VWR on skeletal muscle, and the evaluation occurred after 15 days of constant darkness [30], in contrast to our studies in mice, which were exposed to 12 h of alternate light and dark cycles. In the oral hyperammonaemia model, blood ammonia increased by ~50%, and tissue concentrations were not reported. In contrast, we developed a model to replicate the tissue concentrations of ammonia in human cirrhosis, demonstrating the translational relevance of our data. Combined, these data indicate that hyperammonaemia affects the activity and time of onset in mice in a context‐dependent manner. Others have reported that even a 1‐h shift in circadian patterns is clinically significant [33], demonstrating the high relevance of our studies.

Mitochondrial oxidative function is a critical contributor to skeletal muscle protein homeostasis, essential for maintaining muscle mass and the energy demands of contractile function. Hyperammonaemia impairs mitochondrial oxidative function in myotubes and skeletal muscle in preclinical models [7, 9]. Consistently, mitochondrial oxidative function was impaired in skeletal muscle from AmAc‐UA compared with PBS‐UA mice, and VWR partially reversed these responses. However, mitochondrial oxidative function did not change with VWR in the PBS mice. These data differ from reports by others that VWR improves some measures of mitochondrial oxidative function [34], which may be related to longer durations of VWR [34]. Lower skeletal muscle expression of components of the ETC in AmAc‐UA than PBS‐UA mice in the present studies is consistent with previous reports in hyperammonemic myotubes [10], and the present studies show that VWR partially restored expression and functional consequences in AmAc‐treated mice, suggesting that VWR increases mitochondrial efficiency during hyperammonaemia.

Impaired mitochondrial oxidative function contributes to decreased protein synthesis and increased autophagy in hyperammonaemia [9]. Less global protein synthesis, increased autophagy markers, and altered expression of molecules regulating protein homeostasis in skeletal muscle from AmAc‐UA compared with PBS‐UA mice are recognized effects of hyperammonaemia [5, 9, 10, 19]. Higher global protein synthesis with VWR compared with UA (in both PBS‐ and AmAc‐treated mice) was different from reports by others that endurance exercise results in less muscle protein synthesis but similar to those by others who report increased protein synthesis [1, 35]. These differences may be related to the duration/intensity of exercise and the age of the mice in other studies. Our findings of increased skeletal muscle autophagy markers in AmAc‐UA than PBS‐UA mice are similar to those reported earlier [19]. Similarly, increased expression of autophagy markers in VWR than UA mice, irrespective of treatment, is also consistent with prior reports that exercise increases muscle autophagy [36]. Even though we did not measure autophagy flux, our prior studies showed concordance between flux and static measures of skeletal muscle autophagy [19]. Also, we and others have used colchicine to measure autophagy flux in vivo [19], but concerns about colchicine‐induced mitochondrial dysfunction (Reference S9) and its impact on the interpretation of mitochondrial responses led us to employ static measures of autophagy in the present studies. Phosphorylation status and crosstalk between mTORC1, AMPK, and eIF2α signalling pathways [5, 18] (Reference S3) regulate muscle protein synthesis and autophagy. Responses to AmAc compared with PBS in UA mice were consistent with prior reports of decreased mTOR phosphorylation and increased phosphorylation of AMPK and eIF2α, which contribute to less muscle protein synthesis and increased autophagy markers during hyperammonaemia [18]. Also, VWR increased phosphorylation of mTOR and mTORC1 signalling. Expectedly, phosphorylation of AMPK was increased with VWR, which is consistent with prior reports (Reference S10). Simultaneous increases in phosphorylation of AMPK and mTOR with VWR, as noted in our study, have been reported in other models also and may be due to altered phosphatase activity [37] and need to be evaluated in future studies. Impaired protein synthesis in endurance exercise is believed to be due to increased phosphorylation of AMPK [1, 22], although higher muscle protein synthesis and hypertrophy have also been reported (Reference S11).

Consistent with prior reports [4, 6, 11], circulating and muscle ammonia concentrations were higher in the AmAc than in PBS‐treated mice, and with VWR. Expression of the regulated ammonia transporter, RhBG, increased only in AmAc‐UA mice and was reversed with VWR, which is similar to reports by others during hyperammonaemia and response to exercise in mice [38]. Increased GDH, GS, GLS, and P5CS expression during hyperammonaemia suggests an adaptive response for nonureagenic ammonia disposal. Expression of GDH, GS, and P5CS was unaltered, while expression of GLS and AST was lower in AmAc‐VWR than in PBS‐VWR. These differences may be due to differences in ammonia metabolism or context‐specific adaptive exhaustion of ammonia disposal pathways. Our data also suggest that during hyperammonaemia, adaptive exhaustion of muscle ammonia disposal responses may result in altered molecular responses and potentially limit the benefits of exercise, as reported earlier (Reference S3). Correlation of contributing variables and outcomes of interest showed that, consistent with our observations, VWR in AmAc mice is positively related to a number of adaptive responses, including mitochondrial oxidative function and ammonia disposal. The mechanistic basis of these novel observations needs to be evaluated in the future.

Potential limitations include studies being performed only in male and wild‐type mice. Male mice were evaluated to avoid the variability induced by the oestrous cycle. Additionally, the present studies focused on the impact of hyperammonaemia, a consistent metabolic abnormality in a number of chronic diseases [5]. Other contributors to muscle loss and contractile dysfunction in chronic diseases, including hormonal changes, endotoxaemia, and perturbations in blood flow, have not been evaluated. Our data on the adaptive responses to hyperammonaemia also suggest the need for studies in mice with genetic/pharmacological modifications of ammonia disposal, mitochondrial oxidative responses (e.g., PGC1α), and protein homeostasis (mTORC1 components, nutrient supplementation, e.g., β‐hydroxymethyl butyrate [39]). How exactly VWR and hyperammonaemia interact to regulate/modify autophagy flux will need to be evaluated in the future, including the use of transgenic (LC3‐GFP) mice. A potential control could be the use of completely sedentary mice, either through a restraint system or genetic modifications, both of which can have effects on several variables that would impact the ability to compare the results with those of the VWR mice. Our use of a locked wheel also provides a control system to evaluate the effects of hyperammonaemia during UA or VWR. The present studies only evaluated the effect of VWR, but other forms of exercise (resistance, concurrent) or duration of exercise may have different responses, especially during hyperammonaemia (Reference S3) [30, 40]. Our studies lay the foundation for evaluating the contribution of these variables in the future.

In summary, VWR did not alter body composition in vehicle‐treated mice but reversed hyperammonaemia‐induced impaired skeletal muscle protein synthesis, lower grip strength, and mitochondrial oxidative dysfunction. Consistently, hyperammonaemia did not impair beneficial responses or running distance in skeletal muscle with VWR. The expression of nonureagenic ammonia disposal enzymes in skeletal muscle was higher during hyperammonaemia, with context‐specific changes with VWR. Thus, our data show beneficial responses to VWR during hyperammonaemia, with a limited impact of hyperammonaemia on VWR. Our studies lay the foundation for exercise programmes, despite perturbed ammonia metabolism, noted in chronic diseases (graphical abstract: Figure 7E).

## Conflicts of Interest

The authors declare no conflicts of interest.

## Supporting information


**Data S1:** Supplementary Information.

**Figure S1:** **Study design and organ weights**. Studies were performed in male C57BL6/J mice aged 8–10 weeks treated with either sterile phosphate‐buffered saline (PBS, vehicle) or 2.5 mmol.kg^−1^.d^−1^ ammonia acetate (AmAc) for 42 days. 2 weeks post‐pump placement, mice were randomized to interventions (usual activity, UA, or voluntary wheel running, VWR) for 4 weeks. Grip strength and body weight were measured weekly. Metabolic and activity measurements **were** measured by **the** CLAMS metabolic cage system for 48 h**, and** body composition by EchoMRI prior to and post‐intervention. Mice were sacrificed at the end of the intervention, and tissues were harvested and weighed. Clinical lab parameters were measured. **A**. Schematic of study design. **B**. **Lean body mass (top) and fat mass pre‐ and post‐intervention, and change in fat and lean mass and overall body weight**. **C**. Average total, lean, and fat mass pre‐ to post‐intervention in different groups of mice. **D**. Percent lean and fat mass (measured by EchoMRI) as a proportion of total body weight. **E**. Plasma and skeletal muscle ammonia concentrations. **F**. Blood concentrations of alanine aminotransferase (ALT), aspartate aminotransferase (AST), blood urea nitrogen (BUN), glucose, and insulin. **G**. **Representative photomicrographs and quantification of fibre typing by immunohistochemistry for Type I, IIA, IIB, and IIX fibres**. **Scale bar = 100um**. **H**. **Representative immunoblots and densitometry for Agrin protein**. **I**. Organ weights. **Average weights of organs in different groups**. All data expressed as mean ± SD ***Statistical analysis***: **The one‐sample Kolmogorov–Smirnov test was performed to test if the data distribution was normal**. **Panels B and C**. **A paired t‐test was used for two‐group comparisons, while for multiple‐group comparisons, one‐way ANOVA followed by Tukey’s post hoc analysis was performed. Panel D. Blood ammonia concentrations, one‐way ANOVA followed by Tukey’s post hoc analysis was performed. For skeletal muscle ammonia concentrations, Kruskal‐Wallis, followed by Dunn’s Multiple Comparison test, was performed. Panel E. Kruskal‐Wallis followed by Dunn’s Multiple Comparison was performed for ALT, AST, and insulin; For BUN and glucose, one‐way ANOVA followed by Tukey post hoc test was used. Panels F‐I, One‐way ANOVA followed by Fisher uncorrected LSD was used for post hoc analysis.** * *p* < 0.05; **p < 0.01; *** *p* < 0.001. *n* = 8 in each group.
**Figure S2: Average run distance and total activity in X‐direction.** Studies were performed in male C57BL6/J mice aged 8–10 weeks treated with either sterile phosphate‐buffered saline (PBS, vehicle) or 2.5 mmol.kg^−1^.d^−1^ ammonia acetate (AmAc) for 42 days. 2 weeks post‐pump placement, mice were randomized to interventions (usual activity, UA, or voluntary wheel running, VWR) for 4 weeks. Total activity in the X**‐** (length of the cage) **and Z‐ (height of the cage) directions** w**ere** measured in a CLAMS metabolic cage by light beam breaks post‐intervention (VWR or UA) **and total running distance in meters was measured during the 28 days of intervention**: **A. Violin plot of average run distance (meters) in the intervention groups of mice at week 1, week 2, week 3, and week 4. B. Average pre‐intervention of total X‐activity of all mice.** All data expressed as mean ± SD**. *Statistical analysis***: **The Shapiro–Wilk test was performed to evaluate if the data distribution was normal. Since criteria for normality were not satisfied for these data, the Aligned Rank Transform (ART) for nonparametric two‐way ANOVA analysis to determine the independent effect of: A. Treatment (PBS versus AmAc), time (weeks 1–4), and whether an interaction effect exists between treatment and time; B. Treatment and intervention (UA vs VWR), and whether an interaction effect exists between treatment and intervention. Post hoc analysis was performed with Dunn’s test with Benjamini‐Hochberg post hoc correction.** * *p* < 0.05; **p < 0.01; *** *p* < 0.001 unless otherwise stated. *N* = 8 in each group.
**Figure S3: Total activity in X‐direction.** Studies were performed in male C57BL6/J mice aged 8–10 weeks treated with either sterile phosphate‐buffered saline (PBS, vehicle) or 2.5 mmol.kg^−1^.d^−1^ ammonia acetate (AmAc) for 42 days. 2 weeks post‐pump placement, mice were randomized to interventions (usual activity, UA, or voluntary wheel running, VWR) for 4 weeks. Total activity in the X**‐**direction (length of the cage) was measured in a CLAMS metabolic cage by light beam breaks pre‐ and post‐intervention (VWR or UA). **A. Total X‐activity post‐intervention; B‐E:** Average total X‐activity pre‐ to post‐intervention in: **B.** PBS‐UA mice. **C.** AmAc‐UA mice. **D.** PBS‐VWR. **E**. AmAc‐VWR mice. All data expressed as mean ± SD**. *Statistical analysis*. For Panel A, the Shapiro–Wilk test was performed to evaluate if the data distribution was normal. Since criteria for normality were not satisfied for these data, the Aligned Rank Transform (ART) for nonparametric two‐way ANOVA analysis to determine the independent effect of treatment (PBS versus AmAc), and intervention (UA vs VWR) and whether an interaction effect exists between treatment and intervention was used. Post hoc analysis was performed using Dunn’s test with Benjamini‐Hochberg post hoc correction. Panels B‐E. The one‐sample Kolmogorov–Smirnov test was used to test if the data distribution was normal, followed by a paired t‐test for pre‐intervention to post‐intervention data.** * *p* < 0.05; **p < 0.01; *** *p* < 0.001 unless stated **otherwise**. *N* = 8 in each group.
**Figure S4: Ambulatory activity in the X‐direction.** Studies were performed in male C57BL6/J mice aged 8–10 weeks treated with either sterile phosphate‐buffered saline (PBS, vehicle) or 2.5 mmol.kg^−1^.d^−1^ ammonia acetate (AmAc) for 42 days. 2 weeks post‐pump placement, mice were randomized to interventions (usual activity, UA, or voluntary wheel running, VWR) for 4 weeks. Ambulatory activity in the X‐direction (length of the cage) was measured in a CLAMS metabolic cage by light beam breaks pre‐ and post‐intervention (VWR or UA). Average ambulatory X‐activity pre‐intervention of: **A.** All mice. **B‐E:** Average ambulatory X‐activity pre‐ to post‐intervention in **B.** PBS‐UA mice. **C.** AmAc‐UA mice. **D.** PBS‐VWR. **E**. AmAc‐VWR mice. **F.** Bar graph showing ambulatory and non‐ambulatory X‐activity as a percent of total X‐activity. All data expressed as mean±SD ***Statistical analysis*. For Panel A, the Shapiro–Wilk test was used to evaluate whether the data were normally distributed. Since the activity data were not normally distributed, the Aligned Rank Transform (ART) for nonparametric two‐way ANOVA analysis to determine the independent effect of treatment (PBS versus AmAc), and intervention (UA vs VWR), and whether an interaction effect exists between treatment and intervention was used. Post hoc analysis was performed using Dunn’s test with Benjamini‐Hochberg post hoc correction. Panels B‐F. The one‐sample Kolmogorov–Smirnov test was used to test if the data distribution was normal. Panels B‐E, the paired t‐test was performed for pre‐intervention to post‐intervention data. Panel F. One‐way ANOVA followed by Tukey post hoc test was used.** * *p* < 0.05; **p < 0.01; *** *p* < 0.001 unless otherwise stated. *N* = 8 in each group.
**Figure S5: Total activity in the Z‐direction.** Studies were performed in male C57BL6/J mice aged 8–10 weeks treated with either sterile phosphate‐buffered saline (PBS, vehicle) or 2.5 mmol.kg^−1^.d^−1^ ammonia acetate (AmAc) for 42 days. 2 weeks post‐pump placement, mice were randomized to interventions (usual activity, UA, or voluntary wheel running, VWR) for 4 weeks. Activity in the Z**‐**direction (height of the cage) was measured in a CLAMS metabolic cage by light beam breaks pre‐ and post‐intervention (VWR or UA). Average **total** Z‐activity of **A. P**re‐intervention**; B. Post‐intervention comparing PBS‐UA/AmAc‐UA; C‐F:** Average Z‐activity pre‐ to post‐intervention in **C.** PBS‐UA mice. **D.** AmAc‐UA mice. **E.** PBS‐VWR. **F**. AmAc‐VWR mice**; G.** Line graph showing **zoomed views activity at** time 334 to 720 and 1440 to 1800 min to highlight observed differences in Z‐activity, with bar graphs representing each of the 18 min time points between the two groups or the average of all time points between 334 to 720 and 2800 **to** 1440 min. All data expressed as mean±SD. *Statistical analysis*. For Panels A and B, the Shapiro–Wilk test was used to evaluate if the data were normally distributed. Since the activity data were not normally distributed, the Aligned Rank Transform (ART) for nonparametric two‐way ANOVA analysis to determine the independent effect of treatment (PBS versus AmAc), and intervention (UA vs VWR), and whether an interaction effect exists between treatment and intervention was used. Post hoc analysis was performed using Dunn’s test with Benjamini‐Hochberg post hoc correction. Panels C‐G. The one‐sample Kolmogorov–Smirnov test was used to determine if the data distribution was normal. For Panels C‐F, pre‐intervention to post‐intervention data, the paired t‐test was performed. Panel G. Unpaired t‐test for independent groups. * *p* < 0.05; **p < 0.01; *** *p* < 0.001 unless otherwise stated. *N* = 8 in each group.
**Figure S6: Correlation of Activity Data.** Studies were performed in male C57BL6/J mice aged 8–10 weeks treated with either sterile phosphate‐buffered saline (PBS, vehicle) or 2.5 mmol.kg^−1^.d^−1^ ammonia acetate (AmAc) for 42 days. 2 weeks post‐pump placement, mice were randomized to interventions (usual activity, UA, or voluntary wheel running, VWR) for 4 weeks. Total activity in the X‐direction (length of the cage), ambulatory activity in the X‐direction (length of the cage), and activity in the Z‐direction (height of the cage) were measured in a CLAMS metabolic cage by light beam breaks post‐intervention (VWR or UA). The number of wheel rotations measured total wheel rotations, and the zeitgeber time (ZT) of rotations was measured over the 28‐day intervention period. **A‐D** Correlation plot of total X‐activity to ambulatory **X**‐activity, total **X**‐activity to **Z**‐activity and ambulatory **X**‐activity to **Z**‐activity in: **A.** PBS‐UA mice. **B.** AmAc‐UA mice. **C.** PBS‐VWR mice. **D**. AmAc‐VWR mice. **E,F** Correlation plot of wheel running activity (in VWR mice only) to total x‐activity, ambulatory **X**‐activity and **Z**‐activity in**. E**. PBS‐VWR mice. **F.** AmAc‐VWR mice. ***Statistical analyses*.** All data expressed as **a** correlation plot of XY data: Pearson’s correlation analysis with r value calculation.*p < 0.05; **p < 0.01; ***p < 0.001 unless otherwise stated. *N* = 8 in each group.
**Figure S7: Wheel contact activity during hyperammonemia. Studies were performed in male C57BL6/J mice aged 8–10 weeks treated with either sterile phosphate‐buffered saline (PBS, vehicle) or 2.5 mmol.kg** ^**−1**^ **.d** ^**−1**^ ammonia acetate (AmAc) for 42 days. 2 weeks post‐pump placement, mice were randomized to interventions (usual activity, UA, or voluntary wheel running, VWR) for 4 weeks. The distance in meters and the zeitgeber time (ZT) of rotations were measured over the 28‐day intervention period. Hourly meters run for VWR mice (*n* = 6 in each group) for 10 of the 28 days of the intervention period (left) with double‐plotted averages for 6 mice over 40 h of running measurements (right). *Statistical analyses*. The one‐sample Kolmogorov–Smirnov test was used to test for normality. Since the data were not normally distributed, the 2‐sample Kolmogorov–Smirnov test was used for comparisons. * *p* < 0.05; **p < 0.01; *** *p* < 0.001 unless stated within the figure. *N* = 8 mice in each group.
**Figure S8: Circadian patterns of wheel rotations. Studies were performed in male C57BL6/J mice aged 8–10 weeks, treated with either sterile phosphate‐buffered saline (PBS, vehicle) or 2.5 mmol.kg** ^**−1**^ **.d** ^**−1**^ ammonia acetate (AmAc) for 42 days. 2 weeks post‐pump placement, mice were randomized to interventions (usual activity, UA, or voluntary wheel running, VWR) for 4 weeks. A. Hourly wheel rotations for VWR mice (*n* = 6 in each group) for 10 of the 28 days of the intervention period B. Average running distance per hours 10–18 during the dark cycle of days 3–5 and 10–11 with peak running time noted on each day showing the difference of in running activity between the two groups. C. Average wheel rotations per hour 0–9 during the light cycle on days 5–7, showing the difference in running activity between the two groups. C. Average wheel rotations per hour 0–9 during the light cycle on days 5–7, showing the difference in running activity between the two groups. D. Average rotations during the dark and light cycle between the two groups of mice. Grey bars on the line graphs represent the dark cycle, and the white background represents the light cycle. All data expressed as mean±SD. *Statistical analysis*: The one‐sample Kolmogorov–Smirnov test for normality was used. Panels B‐D. Unpaired t‐test with Welch’s correction was used. * *p* < 0.05; **p< 0.01; *** *p* < 0.001 unless stated within the figure. *N* = 8 mice in each group.
**Figure S9:** Circadian patterns of distance run. Studies were performed in male C57BL6/J mice aged 8–10 weeks treated with either sterile phosphate‐buffered saline (PBS, vehicle) or 2.5 mmol.kg−^1^.d−^1^ ammonia acetate (AmAc) for 42 days. 2 weeks post‐pump placement, mice were randomized to interventions (usual activity, UA, or voluntary wheel running, VWR) for 4 weeks. A. Hourly meters run for VWR mice (n = 6 in each group) for 10 of the 28 days of the intervention period B. Average meters run per hours 10–18 during the dark cycle of days 3–5 and 10–11 with peak running time noted on each day showing the difference of in running activity between the two groups. C. Average meters run per hour 0–9 during the light cycle on days 5–7, showing the difference in running activity between the two groups. D. Average meters run during the dark and light cycle between the two groups of mice. Grey bars on the line graphs represent the dark cycle and the white background represents light cycle. All data expressed as mean±SD. *Statistical analysis*. The one‐sample Kolmogorov–Smirnov test for normality was used. Panels B‐D. An unpaired t‐test with Welch’s correction was used. * *p* < 0.05; **p < 0.01; *** *p* < 0.001 unless stated within the figure. *N* = 8 mice in each group.
**Figure S10:** **Circadian patterns of post‐intervention X‐ and Z‐activity**. Studies were performed in male C57BL6/J mice aged 8–10 weeks treated with either sterile phosphate‐buffered saline (PBS, vehicle) or 2.5 mmol.kg−1.d−1 ammonia acetate (AmAc) for 42 days. 2 weeks post‐pump placement, mice were randomized to interventions (usual activity, UA, voluntary wheel running, VWR) for 4 weeks. Total and ambulatory activity in the X direction (length of the cage) and Z‐activity (height of the cage) w measured in a CLAMS metabolic cage by light beam breaks post‐intervention (VWR or UA): **A**. Total X‐activity post‐intervention as a line graph over time and bar graphs showing total **X**‐activity in the light and dark cycle between each group and the average of light/dark cycle in all 4 groups of mice. **B.** Ambulatory X‐activity post‐intervention as a line graph over time and bar graphs showing ambulatory **X**‐activity in the light and dark cycle between each group and the average of light/dark cycle in all 4 groups of mice. **C.** Z‐activity post‐intervention as a line graph over time and bar graphs showing **Z**‐activity in the light and dark cycle between each group and the average of light/dark cycle in all 4 groups of mice. Grey bars on the line graphs represent dark cycle and white background represents light cycle. All data expressed as mean±SD. **Statistical analysis**. **The one‐sample Kolmogorov–Smirnov test for normality was used. For Total and ambulatory X‐activity, the** Student’s **t**‐test for two groups, and **for four groups**, one‐way ANOVA followed by **Fisher’s least significant difference was used. For total Z‐activity for two groups, an unpaired t‐test was performed, for four groups comparison, Kruskal‐Wallis analysis followed by Dunn’s multiple comparison test was performed**. *p < 0.05; **p < 0.01; ***p < 0.001 unless otherwise stated. *N* = 8 in each group.
**Figure S11:** **Effect of treatment/intervention on VO_2_**. Studies were performed in male C57BL6/J mice aged 8–10 weeks treated with either sterile phosphate buffered saline (PBS, vehicle) or 2.5 mmol.kg^−1^.d^−1^ ammonia acetate (AmAc) for 42 days. 2 weeks post‐pump placement, mice were randomized to interventions (usual activity, UA, or voluntary wheel running, VWR) for 4 weeks. Volume of oxygen inhaled (VO2) was measured in a CLAMS metabolic cage pre‐ and post‐intervention (VWR or UA). **A**. Average VO2 pre‐intervention in all mice. **B. VO2 postintervention in all mice**. **C‐F** Pre‐ to post‐ intervention VO2 in **C**. PBS‐UA mice. **D**. AmAc‐UA mice. **E**. PBS‐VWR. **F**. AmAc‐VWR mice. All data expressed as mean±SD. **Statistical analysis**. **Panels A,B: Based on the Shapiro–Wilk test, the metabolic data distribution was not normal. The Aligned Rank Transform (ART) for nonparametric two‐way ANOVA analysis was used to determine the independent effect of treatment (PBS versus AmAc), and intervention (UA vs VWR), and whether an interaction effect exists between treatment and intervention. Post hoc analysis was performed using Dunn’s test with BenjaminiHochberg post hoc correction. For pre‐ to post‐intervention data, a one sample Kolmogorov–Smirnov test for normality was followed by a paired t‐test (panels C,D, and F). For panel E, the Wilcoxon signed‐rank test was used.** *p < 0.05; **p < 0.01; ***p < 0.001 unless otherwise stated. *N* = 8 in each group.
**Figure S12:** **Effect of treatment/intervention on VCO_2_.** Studies were performed in male C57BL6/J mice aged 8–10 weeks treated with either sterile phosphate buffered saline (PBS, vehicle) or 2.5 mmol.kg−1.d−1 ammonia acetate (AmAc) for 42 days. 2 weeks post‐pump placement, mice were randomized to interventions (usual activity, UA, or voluntary wheel running, VWR) for 4 weeks. Volume of carbon dioxide exhaled (VCO2) was measured in a CLAMS metabolic cage pre‐ and postintervention (VWR or UA). **A**. Pre‐intervention VCO2 of all mice. **B. VCO_2_ postintervention in all mice. C‐F:** Pre‐ to post‐intervention VCO2 in **C**. PBS‐UA mice. **D**. AmAc‐UA mice. **E**. PBS‐VWR. F. AmAc‐VWR mice. All data expressed as mean±SD. **Statistical analysis. Panels A,B: Based on the Shapiro–Wilk test, the metabolic data distribution was not normal. The Aligned Rank Transform (ART) for nonparametric two‐way ANOVA analysis was used to determine the independent effect of treatment (PBS versus AmAc), and intervention (UA vs VWR), and whether an interaction effect exists between treatment and intervention. Post hoc analysis was performed with Dunn’s test with BenjaminiHochberg post hoc correction. For pre‐ to post‐intervention data, a Kolmogorov–Smirnov test for normality distribution was followed by a paired ttest (panels C‐F).** *p < 0.05; **p < 0.01; ***p < 0.001 unless otherwise stated. *N* = 8 in each group.
**Figure S13: Effect of treatment/intervention on respiratory exchange ratio (RER)**. Studies were performed in male C57BL6/J mice aged 8–10 weeks treated with either sterile phosphate buffered saline (PBS, vehicle) or 2.5 mmol.kg^−1^.d^−1^ ammonia acetate (AmAc) for 42 days. 2 weeks post‐pump placement, mice were randomized to interventions (usual activity, UA, or voluntary wheel running, VWR) for 4 weeks. Respiratory exchange ratio (RER) was measured in a CLAMS metabolic cage pre‐ and post‐intervention (VWR or UA). **A**. Average RER of all mice preintervention. **B**. Average RER of all mice post‐intervention. C‐F Pre‐ to postintervention RER in **C**. PBS‐UA mice. **D**. AmAc‐UA mice. **E**. PBS‐VWR. F. AmAcVWR mice. All data expressed as mean±SD **Statistical analysis. Panels A,B: Based on the Shapiro–Wilk test, the metabolic data distribution was not normal. The Aligned Rank Transform (ART) for nonparametric two‐way ANOVA analysis was used to determine the independent effect of treatment (PBS versus AmAc), and intervention (UA vs VWR), and whether an interaction effect exists between treatment and intervention. Post hoc analysis was performed with Dunn’s test with Benjamini‐Hochberg post hoc correction. For pre‐ to post‐intervention data, a Kolmogorov–Smirnov test for normality distribution was followed by a paired t‐test (panels C‐F).** *p < 0.05; **p < 0.01; ***p < 0.001 unless otherwise stated. *N* = 8 in each group.
**Figure S14:** **Circadian patterns of post‐intervention metabolic measures**. Studies were performed in male C57BL6/J mice aged 8–10 weeks treated with either sterile phosphate‐buffered saline (PBS, vehicle) or 2.5 mmol.kg^−1^.d^−1^ ammonia acetate (AmAc) for 42 days. 2 weeks post‐pump placement, mice were randomized to interventions (usual activity, UA, or voluntary wheel running, VWR) for 4 weeks. Volume of oxygen inhaled (VO_2_), volume of carbon dioxide exhaled (VCO_2_), and respiratory exchange ratio (RER) was measured in a CLAMS metabolic cage postintervention (VWR or UA): **A**. VO_2_, post‐intervention as a line graph over time and bar graphs showing total VO_2_ in the light and dark cycle between each group and the average of light/dark cycle in all 4 groups of mice. **B**. VCO_2_ post‐intervention as a line graph over time and bar graphs showing VCO_2_ in the light and dark cycle between each group and the average of light/dark cycle in all 4 groups of mice. **C**. RER post‐intervention as a line graph over time and bar graphs showing RER in the light and dark cycle between each group and the average of light/dark cycle in all 4 groups of mice. Grey bars on the line graphs represent the dark cycle, and the white background represents the light cycle. All data expressed as mean±SD. **Statistical analysis. The one‐sample Kolmogorov–Smirnov test for normality was used. For two‐group comparisons, the Student’s t‐test, and for four‐group comparisons, one‐way ANOVA followed by Fisher’s least significant difference was used. For comparisons of light–dark cycle in the AmAc‐UA group in Panel A and PBSVWR group (Panel C), a Mann–Whitney test was used.** *p < 0.05; **p < 0.01; ***p < 0.001 unless otherwise stated. *N* = 8 in each group.
Figure S15: Pre‐intervention energy expenditure (EE). Studies were performed in male C57BL6/J mice aged 8–10 weeks treated with either sterile phosphatebuffered saline (PBS, vehicle) or 2.5 mmol.kg^−1^.d^−1^ ammonia acetate (AmAc) for 42 days. 2 weeks post‐pump placement, mice were later randomized to interventions (usual activity, UA, or voluntary wheel running, VWR) for 4 weeks. Energy Expenditure (EE) was measured in in a CLAMS metabolic cage). Average EE of all mice pre‐intervention is shown. All data expressed as mean±SD. Statistical Analyses. Based on the Shapiro–Wilk normality test, EE data were not normally distributed. Therefore, we used the Aligned Rank Transform (ART) for nonparametric two‐way ANOVA analysis to determine the independent effect of treatment (PBS versus AmAc), and intervention (UA vs VWR), and whether an interaction effect exists between treatment and intervention. Post hoc analysis was performed with Dunn’s test with Benjamini Hochberg post hoc correction.* *p* < 0.05; **p < 0.01; *** *p* < 0.001 unless otherwise stated. *N* = 8 in each group.
**Figure S16: Post‐intervention energy expenditure (EE)**. Studies were performed in male C57BL6/J mice aged **8–10 weeks** treated with either sterile phosphate buffered saline (PBS, vehicle) or 2.5 mmol.kg^−1^.d^−1^ammonia acetate (AmAc) for 42 days. 2 weeks post‐pump placement, mice were randomized to interventions (usual activity, UA, or voluntary wheel running, VWR) for 4 weeks. Energy Expenditure (EE) was measured in a CLAMS metabolic cage **post**‐intervention (VWR or UA). **A**. Average EE . **B**. **Total** EE . All data expressed as mean±SD. **Statistical analysis. For Panels A,B, Shapiro–Wilk normality test showed that the data were not normally distributed. The Aligned Rank Transform (ART) for nonparametric two‐way ANOVA analysis to determine the independent effect of treatment (PBS versus AmAc), and intervention (UA vs VWR), and whether an interaction effect exists between treatment and intervention. Post hoc analysis was performed using Dunn’s test with Benjamini‐Hochberg post hoc correction**. *p < 0.05; **p < 0.01; *** *p* < 0.001 unless otherwise stated. *N* = 8 in each group.
**Figure S17: Effect of treatment/intervention on energy expenditure**. Studies were performed in male C57BL6/J mice aged 8–10 weeks treated with either sterile phosphate‐buffered saline (PBS, vehicle) or 2.5 mmol.kg^−1^.d^−1^ ammonia acetate (AmAc) for 42 days. 2 weeks post‐pump placement, mice were randomized to interventions (usual activity, UA, or voluntary wheel running, VWR) for 4 weeks. Energy Expenditure (EE) was measured in a CLAMS metabolic cage pre‐ and postintervention (VWR or UA). **A‐D** Pre‐ to post‐intervention EE in **A**. PBS‐UA mice. **B**. AmAc‐UA mice. **C**. PBS‐VWR. **D**. AmAc‐VWR mice. All data expressed as mean±SD. **Statistical analysis. The Kolmogorov–Smirnov test for normality distribution was followed by a paired t‐test For pre‐ to post‐intervention data,.** *p < 0.05; **p < 0.01; *** *p*< 0.001 unless otherwise stated. *N* = 8 in each group.
Figure S18: Circadian patterns of post‐intervention energy expenditure. All studies were conducted in **8–10‐week‐old** male C57BL6/J mice infused either with vehicle (phosphate‐buffered saline; PBS) or 2.5 mmol/kg.d^−1^ ammonium acetate (AmAc) for 6 weeks. After the initial 2 weeks, mice were randomized to either usual activity (UA) or voluntary wheel running (VWR) for 28 days. EE and the zeitgeber time (ZT) were recorded over the 48 h of data collection. Additionally, the amplitude and phase of the peaks were recorded **A**. EE of mice post‐intervention and bar graphs showing the amplitude and Phase. **B**. EE post‐intervention as a line graph over time and bar graphs showing EE in the light and dark cycle between each group and the average light/dark cycle in all 4 groups of mice. All data expressed as mean±SD **Statistical analysis. The one‐sample Kolmogorov–Smirnov test for normality was used to evaluate whether the data distribution was normal. For circadian data in Panel A, the phase of peaks of data was analysed with one‐way ANOVA, followed by Tukey’s multiple comparison test, and the line graph and amplitude data were analysed using the Kruskal‐Wallis test, followed by Dunn’s multiple comparison test. For panel B, an unpaired Student’s t‐test was used for 2 groups, and for 4 groups, a one‐way ANOVA followed by Fisher’s least significant difference post hoc test was used**. * *p* < 0.05; **p < 0.01; *** *p* < 0.001 unless otherwise stated within the figure. *n* = 8 in each group.
**Figure S19: Effect of treatment/intervention on food intake**. Studies were performed in male C57BL6/J mice aged 8–10 weeks treated with either sterile phosphate buffered saline (PBS, vehicle) or 2.5 mmol.kg^−1^.d^−1^ ammoni**um** acetate (AmAc) for 42 days. 2 weeks post‐pump placement, mice were randomized to interventions (usual activity, UA, or voluntary wheel running, VWR) for 4 weeks. Food intake was measured in a CLAMS metabolic cage pre‐ and post‐intervention (VWR or UA). **A. Pre‐intervention food measurements**. **B. Post**‐intervention food measurements. **C**. Pre‐ vs post‐intervention total food intake. **D**. Pre‐ vs postintervention lowest food intake. **E**. Pre‐ vs post‐intervention highest food intake. **F**. Pre‐ vs post‐intervention average food intake. **G. pre‐ vs.** post‐intervention number of food grabs. All Data expressed as mean±SD. **Statistical analyses. The one‐sample Kolmogorov–Smirnov test was performed to evaluate whether the data istribution was normal**. For Panels A and B: **One**‐way ANOVA followed by Tukey’s post hoc analysis **for normally distributed data (pre‐intervention total and highest food intake, average food obtained, number of food removal activities). All other data were not normally distributed. Hence, a KruskalWallis test followed by Dunn’s multiple comparisons was used**. For Panel C‐G: Student’s paired t‐test for pre‐ to post‐analysis (**except for the data in Panel D, and in Panel F, PBS‐UA where data were not normally distributed and a Wilcoxon’s signed‐rank test was used**). * *p* < 0.05; **p < 0.01; *** *p* < 0.001. *n* = 8 in each group.
**Figure S20: Mitochondrial mass did not change with hyperammonemia or voluntary wheel running. Representative immunoblots and densitometry of citrate synthase (CS) and voltage‐dependent anion channel (VDAC). β‐Actin, used as a loading control, was run on the same membrane as CS and VDAC. Statistical analyses. The one‐sample Kolmogorov–Smirnov test was performed to evaluate whether the data distribution was normal. One‐way ANOVA followed by Tukey’s post hoc analysis.** *N* = 8 mice for each group. * *p* < 0.05; **p < 0.01; *** *p* < 0.001.
**Figure S21:** **Correlation of Wheel Running Data with other outcomes. Studies were performed in male C57BL6/J mice aged 8–10 weeks treated with either sterile phosphate‐buffered saline (PBS, vehicle) or 2.5 mmol.kg^−1^.d^−1^ ammonium acetate (AmAc) for 42 days. 2 weeks post‐pump placement, mice were randomized to interventions (usual activity, UA, or voluntary wheel running, VWR) for 4 weeks. The number of wheel rotations, distance run in meters, and the zeitgeber time (ZT) of rotations were measured over the 28‐day intervention period. In addition, gastrocnemius muscle weight, grip strength, protein expression of electron transport chain complex I and II, Puromycin incorporation, phosphorylated mTOR (pmTOR), phosphorylated S6 Kinase (pS6), phosphorylated eif2α (peif2α), plasma ammonia concentration, skeletal muscle ammonia concentration, expression of glutamine synthase (GS) and aspartate aminotransferase (AST) and mitochondrial function responses to adenosine diphosphate (ADP), glutamate, and succinate were measured at the end of the study. A,B Correlation plot of running distance, gastrocnemius muscle weight, grip strength, protein expression of electron transport chain complex I and II, Puromycin incorporation, pmTOR, pS6, peif2α, plasma ammonia concentration, skeletal muscle ammonia concentration, expression of GS and AST as well as mitochondrial functional responses to ADP, glutamate, and succinate A. PBS‐VWR mice. B. AmAc‐VWR mice. All data expressed as a correlation plot of XY data: Statistical analyses. Pearson’s correlation analysis with r‐values provided.** *p < 0.05; **p < 0.01; ***p < 0.001 unless otherwise stated. *N* = 8 in each group.

**Data S2:** Supplementary Figures.

**Table S1:** Key Reagents.
**Table S2:** ART ANOVA *p* values.
**Table S3:** Serum biochemical data.

**Table S4:** Statistical Table Data Summary.

**Data S3:** Supplementary References.

## References

[1] D. C. Hughes , S. Ellefsen , and K. Baar , “Adaptations to Endurance and Strength Training,” Cold Spring Harbor Perspectives in Medicine 8 (2018): a029769. 28490537 10.1101/cshperspect.a029769 PMC5983157

[2] M. Ney , L. Gramlich , V. Mathiesen , et al., “Patient‐Perceived Barriers to Lifestyle Interventions in Cirrhosis,” Saudi Journal of Gastroenterology 23 (2017): 97–104. 28361840 10.4103/1319-3767.203357 PMC5385724

[3] E. W. Banister , W. Rajendra , and B. J. Mutch , “Ammonia as an Indicator of Exercise Stress Implications of Recent Findings to Sports Medicine,” Sports Medicine 2 (1985): 34–46. 3883458 10.2165/00007256-198502010-00004

[4] J. McDaniel , G. Davuluri , E. A. Hill , et al., “Hyperammonemia Results in Reduced Muscle Function Independent of Muscle Mass,” American Journal of Physiology. Gastrointestinal and Liver Physiology 310 (2016): G163–G170. 26635319 10.1152/ajpgi.00322.2015 PMC4971815

[5] S. Dasarathy and M. Hatzoglou , “Hyperammonemia and Proteostasis in Cirrhosis,” Current Opinion in Clinical Nutrition & Metabolic Care 21 (2018): 30–36. 29035972 10.1097/MCO.0000000000000426 PMC5806198

[6] J. Qiu , S. Thapaliya , A. Runkana , et al., “Hyperammonemia in Cirrhosis Induces Transcriptional Regulation of Myostatin by an NF‐κB‐Mediated Mechanism,” Proceedings of the National Academy of Sciences of the United States of America 110 (2013): 18162–18167. 24145431 10.1073/pnas.1317049110 PMC3831479

[7] G. Davuluri , A. Allawy , S. Thapaliya , et al., “Hyperammonaemia‐Induced Skeletal Muscle Mitochondrial Dysfunction Results in Cataplerosis and Oxidative Stress,” Journal of Physiology 594 (2016): 7341–7360. 27558544 10.1113/JP272796 PMC5157075

[8] A. Kumar , G. Davuluri , R. N. E. Silva , et al., “Ammonia Lowering Reverses Sarcopenia of Cirrhosis by Restoring Skeletal Muscle Proteostasis,” Hepatology 65 (2017): 2045–2058. 28195332 10.1002/hep.29107 PMC5444955

[9] A. Kumar , N. Welch , S. Mishra , et al., “Metabolic Reprogramming During Hyperammonemia Targets Mitochondrial Function and Postmitotic Senescence,” JCI Insight 6 (2021): e154089. 34935641 10.1172/jci.insight.154089 PMC8783680

[10] S. Mishra , N. Welch , M. Karthikeyan , et al., “Dysregulated Cellular Redox Status During Hyperammonemia Causes Mitochondrial Dysfunction and Senescence by Inhibiting Sirtuin‐Mediated Deacetylation,” Aging Cell 22 (2023): e13852. 37101412 10.1111/acel.13852 PMC10352558

[11] E. W. Banister and B. J. Cameron , “Exercise‐Induced Hyperammonemia: Peripheral and Central Effects,” International Journal of Sports Medicine 11, no. Suppl 2 (1990): S129–S142. 2193891 10.1055/s-2007-1024864

[12] J. P. De Bono , D. Adlam , D. J. Paterson , and K. M. Channon , “Novel Quantitative Phenotypes of Exercise Training in Mouse Models,” American Journal of Physiology—Regulatory, Integrative and Comparative Physiology 290 (2006): R926–R934. 16339385 10.1152/ajpregu.00694.2005

[13] G. Manzanares , G. Brito‐da‐Silva , and P. G. Gandra , “Voluntary Wheel Running: Patterns and Physiological Effects in Mice,” Brazilian Journal of Medical and Biological Research 52 (2018): e7830. 30539969 10.1590/1414-431X20187830 PMC6301263

[14] H. Nishimune , J. A. Stanford , and Y. Mori , “Role of Exercise in Maintaining the Integrity of the Neuromuscular Junction,” Muscle & Nerve 49 (2014): 315–324. 24122772 10.1002/mus.24095 PMC4086464

[15] C. Glass , P. Hipskind , C. Tsien , et al., “Sarcopenia and a Physiologically Low Respiratory Quotient in Patients With Cirrhosis: A Prospective Controlled Study,” Journal of Applied Physiology 114, no. 5 (2013): 559–565. 23288550 10.1152/japplphysiol.01042.2012 PMC3615594

[16] H. W. Chen and M. A. Dunn , “Muscle at Risk: The Multiple Impacts of Ammonia on Sarcopenia and Frailty in Cirrhosis,” Clinical and Translational Gastroenterology 7 (2016): e170. 27228401 10.1038/ctg.2016.33 PMC4893684

[17] J. M. Memme , A. T. Erlich , G. Phukan , and D. A. Hood , “Exercise and Mitochondrial Health,” Journal of Physiology 599 (2021): 803–817. 31674658 10.1113/JP278853

[18] G. Davuluri , D. Krokowski , B. J. Guan , et al., “Metabolic Adaptation of Skeletal Muscle to Hyperammonemia Drives the Beneficial Effects of l‐Leucine in Cirrhosis,” Journal of Hepatology 65 (2016): 929–937. 27318325 10.1016/j.jhep.2016.06.004 PMC5069194

[19] J. Qiu , C. Tsien , S. Thapalaya , et al., “Hyperammonemia‐Mediated Autophagy in Skeletal Muscle Contributes to Sarcopenia of Cirrhosis,” American Journal of Physiology. Endocrinology and Metabolism 303 (2012): E983–E993. 22895779 10.1152/ajpendo.00183.2012 PMC3469607

[20] S. Mishra , N. Welch , S. S. Singh , et al., “Ammonia Transporter RhBG Initiates Downstream Signaling and Functional Responses by Activating NFκB,” Proceedings of the National Academy of Sciences of the United States of America 121 (2024): e2314760121. 39052834 10.1073/pnas.2314760121 PMC11294993

[21] S. Dasarathy , “Myostatin and Beyond in Cirrhosis: All Roads Lead to Sarcopenia,” Journal of Cachexia, Sarcopenia and Muscle 8 (2017): 864–869. 29168629 10.1002/jcsm.12262 PMC5700432

[22] J. A. Hawley , M. Hargreaves , M. J. Joyner , and J. R. Zierath , “Integrative Biology of Exercise,” Cell 159 (2014): 738–749. 25417152 10.1016/j.cell.2014.10.029

[23] V. A. Lira , M. Okutsu , M. Zhang , et al., “Autophagy Is Required for Exercise Training‐Induced Skeletal Muscle Adaptation and Improvement of Physical Performance,” FASEB Journal 27 (2013): 4184–4193. 23825228 10.1096/fj.13-228486 PMC4046188

[24] C. E. Morris , J. C. Garner , S. G. Owens , M. W. Valliant , H. Debusk , and M. Loftin , “A Prospective Study Comparing Distance‐Based vs. Time‐Based Exercise Prescriptions of Walking and Running in Previously Sedentary Overweight Adults,” International Journal of Exercise Science 10 (2017): 782–797. 28966715 10.70252/YJIX2289 PMC5609661

[25] B. J. Mutch and E. W. Banister , “Ammonia Metabolism in Exercise and Fatigue: A Review,” Medicine and Science in Sports and Exercise 15 (1983): 41–50. 6341752

[26] S. M. Marandi , N. G. Abadi , F. Esfarjani , H. Mojtahedi , and G. Ghasemi , “Effects of Intensity of Aerobics on Body Composition and Blood Lipid Profile in Obese/Overweight Females,” International Journal of Preventive Medicine 4 (2013): S118–S125. 23717761 PMC3665017

[27] D. L. Allen , B. C. Harrison , A. Maass , M. L. Bell , W. C. Byrnes , and L. A. Leinwand , “Cardiac and Skeletal Muscle Adaptations to Voluntary Wheel Running in the Mouse,” Journal of Applied Physiology 90, no. 5 (2001): 1900–1908. 11299284 10.1152/jappl.2001.90.5.1900

[28] R. C. McMullan , S. A. Kelly , K. Hua , et al., “Long‐Term Exercise in Mice Has Sex‐Dependent Benefits on Body Composition and Metabolism During Aging,” Physiological Reports 4 (2016): e13011. 27905293 10.14814/phy2.13011 PMC5112492

[29] Z. Yan , M. Okutsu , Y. N. Akhtar , and V. A. Lira , “Regulation of Exercise‐Induced Fiber Type Transformation, Mitochondrial Biogenesis, and Angiogenesis in Skeletal Muscle,” Journal of Applied Physiology 110, no. 1 (2011): 264–274. 21030673 10.1152/japplphysiol.00993.2010 PMC3253006

[30] D. Granados‐Fuentes , K. Cho , G. J. Patti , R. Costa , E. D. Herzog , and S. Montagnese , “Hyperammonaemia Disrupts Daily Rhythms Reversibly by Elevating Glutamate in the Central Circadian Pacemaker,” Liver International 43 (2023): 673–683. 36367321 10.1111/liv.15476 PMC9974605

[31] P. Farinatti , A. G. Castinheiras Neto , and P. R. Amorim , “Oxygen Consumption and Substrate Utilization During and After Resistance Exercises Performed With Different Muscle Mass,” International Journal of Exercise Science 9 (2016): 77–88. 27293507 10.70252/AKBM3973 PMC4882463

[32] V. Schefer and M. I. Talan , “Oxygen Consumption in Adult and AGED C57BL/6J Mice During Acute Treadmill Exercise of Different Intensity,” Experimental Gerontology 31 (1996): 387–392. 9415121 10.1016/0531-5565(95)02032-2

[33] H. J. Burgess , C. S. Legasto , L. F. Fogg , and M. R. Smith , “Can Small Shifts in Circadian Phase Affect Performance?,” Applied Ergonomics 44 (2013): 109–111. 22695081 10.1016/j.apergo.2012.05.007 PMC3475646

[34] C. P. Hedges , D. J. Bishop , and A. J. R. Hickey , “Voluntary Wheel Running Prevents the Acidosis‐Induced Decrease in Skeletal Muscle Mitochondrial Reactive Oxygen Species Emission,” FASEB Journal 33 (2019): 4996–5004. 30596520 10.1096/fj.201801870R

[35] A. R. Konopka and M. P. Harber , “Skeletal Muscle Hypertrophy After Aerobic Exercise Training,” Exercise and Sport Sciences Reviews 42 (2014): 53–61. 24508740 10.1249/JES.0000000000000007 PMC4523889

[36] J. F. Halling and H. Pilegaard , Autophagy‐Dependent Beneficial Effects of Exercise, vol. 7, (Cold Spring Harbor Perspectives in Medicine, 2017): a029777. 10.1101/cshperspect.a029777 PMC5538402 28270532

[37] G. Davuluri , N. Welch , J. Sekar , et al., “Activated Protein Phosphatase 2A Disrupts Nutrient Sensing Balance Between Mechanistic Target of Rapamycin Complex 1 and Adenosine Monophosphate‐Activated Protein Kinase, Causing Sarcopenia in Alcohol‐Associated Liver Disease,” Hepatology 73 (2021): 1892–1908. 32799332 10.1002/hep.31524 PMC8847884

[38] K. Takeda and T. Takemasa , “Expression of Ammonia Transporters Rhbg and Rhcg in Mouse Skeletal Muscle and the Effect of 6‐Week Training on These Proteins,” Physiological Reports 3 (2015): e12596. 26471760 10.14814/phy2.12596 PMC4632962

[39] S. S. Singh , A. Kumar , N. Welch , et al., “Multiomics‐Identified Intervention to Restore Ethanol‐Induced Dysregulated Proteostasis and Secondary Sarcopenia in Alcoholic Liver Disease,” Cellular Physiology and Biochemistry 55 (2021): 91–116. 33543862 10.33594/000000327 PMC8195260

[40] A. Bellar , N. Welch , and S. Dasarathy , “Exercise and Physical Activity in Cirrhosis: Opportunities or Perils,” Journal of Applied Physiology 128 (2020): 1547–1567. 32240017 10.1152/japplphysiol.00798.2019 PMC7311690

