# Supplementary material for: Voluntary Activity Wheel Running Improves Hyperammonaemia‐Induced Skeletal Muscle Molecular and Metabolic Perturbations in Mice
Source: J Cachexia Sarcopenia Muscle. 2025 Aug 4;16(4):e70031. doi: 10.1002/jcsm.70031 (PMC12321975; doi:10.1002/jcsm.70031)
Supplement: Supplementary file 1 — Data S1: Supplementary Information. [file JCSM-16-e70031-s004.docx]

**Supplemental Methods**

**Chemicals**

All chemicals were obtained from Sigma Aldrich (St. Louis, MO, USA), and antibodies were purchased from Cell Signaling Technologies (Danvers, MA, USA) or Proteintech (Rosemont, IL, USA) unless otherwise stated.

**Animal studies**.

Mouse housing and handling.

Mice were housed individually in the Biological Research Unit (BRU) with a 12h light/12h dark cycle and allowed free access to food and water for the duration of the study. After acclimatization for 5 days, baseline non-invasive physiological measurements, including body weight and grip strength. body composition by Echo magnetic resonance imaging (EchoMRI®), whole-body metabolic measures, and food intake were quantified. Mice were randomized to treatment with either sterile phosphate-buffered saline (PBS) or hyperammonemia (2.5 mmol/kg/day of ammonium acetate in PBS) for **42 days**. Mice were allowed to recover for 14 days after pump placement (see Study procedures). Body composition by EchoMRI®, whole-body metabolic measures, and food intake were quantified and then placed in a cage with a vertical wheel for a 2-day acclimatization period with the wheels locked/immobile. Mice (both PBS **and** AmAc) were then randomized to the intervention, defined as usual activity (UA) in which the wheel remained locked or Voluntary Wheel Running VWR for 28 days, for which the number of wheel rotations/minute was measured. Other activities in the cage were not restricted for either group of mice. Body composition, EchoMRI®, whole-body metabolic measures, and food intake were quantified at the end of the study**,** as at baseline. On the study day, mice were euthanized after **obtaining non-invasive physiological measures** (**S.Fig.1A**).

*Animal procedures*. Induction of hyperammonemia. An Alzet mini-osmotic pump Model 2006 (Alzet, Cupertino, CA) was surgically implanted in all mice as described by us[1]. In brief, an Alzet mini-osmotic pump Model 2006 (Alzet, Cupertino, CA) was placed subcutaneously using sterile techniques under isoflurane anesthesia to deliver 2.5 mmol/kg/day of AmAc or PBS for 6 weeks. Wound clips were removed after 10-14 days. **Our model replicates a consistent metabolic perturbation, hyperammonemia, in human chronic disease without the confounding effects of the hepatic necroinflammatory responses and/or endotoxemia during cirrhosis and other chronic diseases**[2-4]**.**

*Noninvasive measurements*

Body weight and grip strength were measured in each mouse prior to the placement of the osmotic pump, before the UA/VWR intervention, and then weekly for 4 weeks during the UA/VWR intervention. Grip strength was measured using a computerized mouse grip strength meter (Columbus Instruments, Columbus, OH, USA) as previously described[5]. Three days after the removal of wound clips, mice were housed individually in cages with a vertical wheel (locked in UA and unlocked for VWR), and cages were placed inside a Circadian Chamber (ActiMetrics, Wilmette, IL). The wheel remained locked for all mice for two days to allow the mice to acclimatize to the cages and wheels. Wheel contact and rotation data were acquired by ClockLab Data Collection and Chamber Control software (Version 4 ActiMetrics, N. Lafayette, IN) and analyzed using Clock Lab Analysis software (Version 3 ActiMetrics, N. Lafayette, IN). Though the mice ran for 28 days, circadian patterns were evaluated for 11 days **after** the mice **had time** to acclimate to the chamber and capture the data during **the plateau of** wheel running activity.

*Evaluation of circadian patterns during exercise*

Hyperammonemia causes circadian perturbations and results in both neuronal activation and inhibition in a dose-dependent manner[6, 7]. The number of contacts the UA mice made with the locked wheel and the number of wheel rotations that occurred in the VWR mice were quantified hourly and plotted over time during the light cycle and dark cycle and evaluated for circadian patterns that occurred. Similar analyses were performed on the CLAMS® data, including total X-activity, ambulatory X-activity, total-Z activity, VO_2_, VCO_2,_ RER, and EE.

**Study day**

After an overnight fast, animals were euthanized between 9 A.M. and 11:30 A.M. to avoid circadian variability of tissue responses. Blood was collected by terminal blood draw from the inferior vena cava into EDTA-coated vials, centrifuged immediately at 2000g for 15 minutes at 4°C, and plasma stored at -80°C for further assays. Gastrocnemius, tibialis anterior, and triceps muscles were harvested, blotted dry of blood, weighed, and a sample was rapidly frozen for metabolic and signaling protein expression studies. A portion of fresh tissue was collected for mitochondrial function studies. Functional and molecular studies were done on the gastrocnemius muscle unless specified. Liver, heart, epididymal fat, kidneys, and testes were collected, blotted dry of blood, weighed, and rapidly frozen and stored at -80°C.

*Echo magnetic resonance imaging (MRI)*

Body composition was measured with an EchoMRI® (EchoMRI-700, EchoMRI, Houston, TX) to determine lean and fat mass, and whole-body water weight per manufacturer instructions. The instrument was calibrated using canola oil, mice were introduced into a ventilated clear plastic tube, and movement was gently restricted. Mice were then placed into the EchoMRI® machine for 2-3 minutes, during which the whole-body hydration ratio was measured as the ratio of (total water–free water)/ (lean body weight). A valid measurement was taken for each mouse**,** and if invalid (based on instrument specifications)**,** the scan was repeated. Additionally, we calculated the change in fat mass, lean mass, and total body weight **by** subtracting post-intervention body weight from pre-intervention body weight.

*Indirect calorimetry*

**The w**hole**-**body metabolic profile was determined **using** an open-flow indirect calorimeter (Comprehensive Laboratory Animal Monitoring System; CLAMS®, Columbus Instruments, Columbus, OH, USA) per manufacturer protocol. In brief, the CLAMS® system was started 2 hours prior to metabolic studies to allow for stabilization and maintenance of temperature. Water bottles and food hoppers were filled to ensure unrestricted food for 2 days. The desiccant was evaluated to ensure accurate CO_2_ measurements. Gas calibrations of CO_2_ and O_2_ were repeated until stable and within the acceptable range per manufacturer protocol. Mice were housed individually in the CLAMS® system and allowed to acclimatize for 24 hours (indicated by stable body weights, food intake, and diurnal metabolic patterns), followed by 24 hours of continuous data acquisition. Metabolic cages were inspected at least once daily to ensure adequate food and water for the mice, the desiccant state, gas parameters, and temperature were within predefined ranges for the experiment, and monitor data acquisition. Rates of oxygen consumption (VO_2_; ml.kg^-1^.h^-1^) and carbon dioxide production (VCO_2_; ml.kg^-1^.h^-1^) were measured every 18 min throughout the study. The respiratory exchange ratio (RER=VCO_2_/CO_2_) was calculated by the CLAMS software (v4.02) to estimate relative substrate oxidation. Energy expenditure (EE) was calculated as EE= VO2x[3.815+(1.232xRER)].

Total activity, which included non-ambulatory activities (*e.g.*, grooming or small movements), and ambulatory activity, were measured separately in a CLAMS® chamber by the number of times the mouse broke a beam of light. **Non-**ambulatory and ambulatory activity were measured separately in the X-direction (cage length) **and** in the Z-direction (cage height)**.** **Total movements detected in the Z-direction are due to beam breaks lengthwise down the cage and higher up and are believed to show rearing or jumping-type activity**[8]**. Measurements were done** every 18 minutes for 48 hours prior to and at the completion of the 4-week intervention period. Additional statistical analyses for Z-activity between the VWR mice were completed by identifying areas on the graph where there were apparent differences in the Z-activity of the mice (minutes 324 to 620 and 1440 to 1800). This was done as the graph identified a clear separation in the activity of the groups, including temporal patterns when activity patterns were divergent between the groups. Total/Ambulatory X-activity, Z-activity, and wheel rotations were correlated to identify if different activity patterns followed similar trends. Specifically, average activity (total/ambulatory X-activity or Z-activity) was plotted against another measure of activity (total/ambulatory X-activity, Z-activity, or wheel running)**.** Pearson’s correlation coefficients correlation plots were generated using Graph Pad Prism. All of the measurements performed in the CLAMS cages were recorded for 48 hours**; however,** to allow for time for the mice to acclimatize to the cages and environment**,** only 30 hours of data was used.

*Food intake*

Food intake was measured in the CLAMS® cages. The food hopper within the cage was filled with food at the start of the study. The CLAMS® cages were equipped with water bottles filled at the start of the study and checked regularly. Mice were allowed free access to food and water. When the mouse obtained food from the hopper, the CLAMS® system measured the amount (grams) and time the mice took food. The number of times, total, lowest, highest, and average amounts of food intake over 48 hours were documented and 30 hours of the data was used as with the other data.

*Evaluation of circadian patterns during exercise*

Analysis of circadian rhythmicity was done at hourly resolution using 3 different pipelines: meta-analysis of 2-d**i**mensional time series data (meta2d)[9], Lomb-Scargle (LS)[10], and Johnker-Halberg-Tukey (JTK)[11] in MetaCycle R package[9]. Phase and amplitude are shown in **Figs. 2, 3, 4, Sup Fig. ,18,** and complete parameters are shown in **Table 1**.

For the activity data measured in CLAMS® cages (total/ambulatory x-activity, z-activity), the metabolic parameters (VO_2_, VCO_2_, RER, and EE), as well as the wheel running data, we divided the data over time into dark and light cycle**s**. For each of the parameters (CLAMS activity, metabolic data, wheel running data), the average of the dark cycle and light cycle for each mouse within one group (PBS UA, AmAc UA, PBS VWR, AmAc VWR) was calculated and then plotted to compare that parameter in the light and dark cycle. Additionally, the average of all mice in each group was compared to the average in the other groups in the dark and light cycles to identify if there were differences in these parameters in **the** light or dark cycle. Additionally for the wheel running data we identified areas on the graphs of Zeitgeber Time (ZT) where there were visually differences in the light and dark cycle (Day 3**-** 7, 10, 11) we compared hours within the dark and light cycle to see if activity was truly different at those specific time points as opposed to overall average off all the days.

*Biochemical assays*

The concentration of ammonia in blood and skeletal muscle in all groups at the end of the study was quantified as described previously[5, 12]. In brief, a fluorometric assay was used to measure ammonia levels by the addition of α-ketoglutarate and quantify oxidation of NADH to NAD^+^ (Sigma-Aldrich AA0100-1KT) **in** plasma and deproteinized gastrocnemius muscle sample**s**. These assays were performed to determine if blood and tissue concentrations of ammonia increased with treatment (AmAc) and VWR and **were** consistent with human tissue levels, as reported earlier[5, 13]. We also determined whether exercise increased hyperammonemia beyond levels induced beyond the infusion levels. Assays were done in all mice and at least 3 technical replicates.

Plasma glucose, blood urea nitrogen, aspartate, and alanine aminotransferase were measured on a Cobas CE6000 c501 module (Roche Diagnostics, Indianapolis, IN) calibrated with appropriate quality control materials. Each sample was loaded onto the analyzer's sample tray and analyzed with appropriate assay protocols.

Plasma insulin levels were measured using an ELISA assay (Mercodia AB, Uppsala, Sweden) according to the manufacturer protocol. In brief, microplate wells were coated with a monoclonal antibody specific for mouse insulin and incubated overnight at 4°C. After the wells were washed, plasma samples and standards were added and incubated for 2 hours at room temperature. After additional washes, polyclonal antibody for mouse insulin was added to the wells and incubated for 1 hour at room temperature. After incubation with streptavidin-horseradish peroxidase (HRP) conjugate, a final wash was performed, and tetramethylbenzidine (TMB) substrate was added for the colorimetric reaction. The reaction was stopped with 0.5M sulfuric acid, and absorbance was measured at 450 nm using a microplate reader (Spectra Max 190, Molecular Devices, San Jose, CA). All assays were done in 3 technical replicates.

***Histomorphometry of muscle tissue*. The area of muscle fiber cross-section and fiber diameter were quantified using the minimum Feret method, as previously described**[14]**.**

**Cryosections. In brief, fresh gastrocnemius muscle tissue was frozen in isopentane prechilled in liquid nitrogen and embedded in Tissue-Tek®optimal cutting temperature (OCT, Sakura Finetek USA Inc., Torrance, CA ) cryo-embedding medium as previously described**[14]**. The frozen blocks were stored at −80 °C until sectioning. Prior to sectioning, tissue blocks were equilibrated in the cryostat chamber for 15–20 minutes to allow temperature stabilization. Cryosections were obtained on a Microm HM 525 cryostat (Thermo Fisher Scientific, Waltham, MA) at −30 °C. Transverse sections of skeletal muscle at a thickness of 10 μm were used for the studies. Each glass microscope slide (Superfrost Plus, Thermo Fisher, Waltham, MA) was loaded with six evenly spaced sections to ensure consistent sample distribution for staining or imaging. Sections were carefully transferred using a fine brush or anti-roll plate to maintain integrity and orientation. Slides were air-dried for 15–30 minutes at room temperature, then either processed immediately for downstream applications or stored at −20 °C in a slide box with desiccant until use.**

**Cross Sectional Area (CSA) and minimum Feret diameter measurement. Sections were stained with hematoxylin and eosin (H&E) stain for assessment of histological architecture and quality control prior to further use, to identify pathological features, and to measure the cross-sectional area (CSA) and minimum Feret diameter of the individual muscle fibers. Digital photographs were taken of each muscle section using a fluorescence microscope (Keyence BZ-X710, Frisco, TX), and the CSA was measured using tools embedded in the Fiji-ImageJ software (NIH)**[15]**. This method allows for the measurement of all of the fibers in the cryostat section. Five different fields per section were analyzed for each muscle sample. Minimum Feret diameter analysis was used fiber diameter**[16]**.**

**Fiber typing and MYHC isoform staining. Fiber typing was determined by immunofluorescence staining for Type I, Type IIA, and Type IIB muscle fibers. After sections were mounted on slides (as above)n and frozen at -80°C. Cross-sections were allowed to reach room temperature and rehydrated with phosphate buffered saline, blocked using goat serum (10% in PBS), and incubated overnight at 4 ºC temperature with the following primary antibody cocktail: MYH7 (bs-24941R, Bioss Inc., Woburn, MA) that recognizes type 1 MHC isoform; MYH2 (SC-71, Developmental Studies Hybridoma Bank, Iowa City, IA) for type 2A MHC isoform; and MYH2 (JE53-30, ThermoFisher Scientific, Waltham, MA), for type 2B MHC isoform. Muscle cross-sections were then washed three times in PBS before being incubated for 1 hour at room temperature with the following secondary antibody cocktail: Alexa Fluor 350 IgG, Alexa Fluor 488 IgG, Alexa Fluor 594 IgG2b, respectively. Muscle cross-sections were washed thrice in PBS, and cover slips were placed using Prolong Gold (Invitrogen, P36930) as mounting medium. Digital photographs of each muscle section were acquired using a fluorescence microscope, as above, at 20x magnification.**

**Image analysis and fiber type quantification. Immunofluorescence-stained muscle cross-sections were imaged at 20X magnification to assess fiber type composition. Images were analyzed using ImageJ, and individual muscle fibers were manually outlined using the freehand selection tool to generate regions of interest (ROIs). Each ROI was added to the ROI Manager to enable consistent channel measurements. After splitting the image into individual fluorescence channels corresponding to specific myosin heavy chain (MyHC) isoforms—MyHC-IIA (green), MyHC-IIB (MyHC4, red), and MyHC-I (blue)—the mean pixel intensity within each ROI was measured in each channel. Fiber type was assigned based on relative intensity: fibers with minimal blue were classified as type IIA, while those with strong blue and low green and red were classified as type I, and those with strong red were not classified as type IIA or type I, but as type IIB. Fibers that were mixed red and green and did not have a strong intensity of any channel were considered presumptive type IIx. Data were exported to Graphpad Prism 10.0 (Graphpad Software Inc, San Diego, CA) for statistical analyses of fiber type distribution.**

*Immunoblots*

Immunoblots for protein expression in muscle tissue were performed as previously described[17]. In brief, muscle tissue was lysed in RIPA buffer, and protein concentrations were quantified using a bicinchoninic acid (BCA) assay (Thermo Scientific, Waltham, MA). Equal amounts of protein samples were separated on tris-glycine gels and electro-transferred to polyvinylidene fluoride (PVDF) membranes (Bio-Rad, Hercules, CA, USA). The primary antibodies used in these studies can be found in **S. Table 1**. Anti-rabbit or anti-mouse antibody conjugated to horseradish peroxidase (Santa Cruz Cat#: rabbit: 70754, mouse: 70767) was used as a secondary antibody (1:10000 dilution). Blots were developed by using an enhanced chemiluminescence reagent (Bio-Rad, Hercules, CA, USA). The protein expression levels were analyzed by measuring the intensity of the band from scanned films using Image-J software[18]. Expression of β-actin was used to normalize the loading of proteins unless otherwise stated.

*Protein synthesis ex-vivo*

*Ex vivo* skeletal muscle protein synthesis was measured by puromycin incorporation as described by us earlier[19]. In brief, approximately 5mg of fresh gastrocnemius muscle tissue was incubated for exactly 30 minutes in 10mL of DMEM with 1mM puromycin and oxygen bubbling into the medium at a constant rate and the tissue was frozen at -80°C. Protein was extracted, immunoblots with antipuromycin antibody (1:10000) were performed, developed using enhanced chemiluminescence (ECL) immunoassay (Bio-Rad, Hercules, CA, USA) and the bands quantified with ImageJ[18]. Blots were normalized using Ponceau-S stained lanes as a loading control.

*Mitochondrial oxidative function in permeabilized skeletal muscle*

Mitochondrial function in permeabilized skeletal muscle was determined by high-sensitivity respirofluorometry using substrate-uncoupler-inhibitor titration (SUIT) protocols as described previously [19, 20]. In brief, approximately 5 mg of fresh gastrocnemius muscle was collected in biopsy preservation solution (BIOPS), incubated in saponin, and washed in mitochondrial respiration medium (MiR05) buffer. Mitochondrial substrates malate, pyruvate, ADP, and glutamate were sequentially added to measure complex I function, succinate as a complex II substrate, and protonophore carbonyl cyanide p-(trifluoromethoxy) phenylhydrazone (FCCP) for uncoupled maximum respiration. Responses to rotenone (complex I inhibitor) and antimycin-A (complex III inhibitor) were measured. The uncoupled complex IV oxidation rate was determined using tetramethyl phenylene diamine (TMPD) and ascorbate. Data were analyzed with DatLab6 (Oroboros, Innsbruck, Austria). All experiments were performed in all the mice (n=8 in each of the 4 groups) and data were expressed as oxygen consumption in pmol.sec^-1^ normalized to tissue weight.

*Statistical methods*. **All data were analyzed for normality using the Kolmogorov-Smirnov test and the Brown-Forsythe test for heteroscedasticity (unequal variances between groups). For normally distributed with equal variances, q**uantitative data were analyzed using the Student’s two-tailed t-test for 2**-**independent samples or one-way analysis of variance (ANOVA), and the least significant difference (LSD) post-hoc tests were used for three or more groups **unless otherwise stated. For data that were skewed and/or had unequal variances across groups (did not satisfy tests of normality and heteroscedasticity even after data transformations), non-parametric tests (Kruskal-Wallis analysis) was performed with post-hoc analysis. The specific test performed for each panel is included within the figure legend.**  For **data comparisons** over time, a Student’s paired “t” test for 2 groups or a repeated measures ANOVA was used. **For activity data and indirect calorimetry data, Align Rank Transform (ART) two way ANOVA was performed, followed by Dunn’s post-hoc analysis.**  Statistical significance was set at p < 0.05. All statistical analyses were **performed using** GraphPad Prism statistical software **10.0** (San Diego, CA, USA) **except for ART two-way ANOVA where the R Statistical Software (V4.0.2) was used.**

1. Kant S, Davuluri G, Alchirazi KA, Welch N, Heit C, Kumar A, et al. Ethanol sensitizes skeletal muscle to ammonia-induced molecular perturbations. J Biol Chem. 2019;294:7231-44.

2. Dasarathy S, Muc S, Hisamuddin K, Edmison JM, Dodig M, McCullough AJ, et al. Altered expression of genes regulating skeletal muscle mass in the portacaval anastomosis rat. Am J Physiol Gastrointest Liver Physiol. 2007;292:G1105-13.

3. Dasarathy S, Dodig M, Muc SM, Kalhan SC, McCullough AJ. Skeletal muscle atrophy is associated with an increased expression of myostatin and impaired satellite cell function in the portacaval anastamosis rat. Am J Physiol Gastrointest Liver Physiol. 2004;287:G1124-30.

4. Dasarathy S, Hatzoglou M. Hyperammonemia and proteostasis in cirrhosis. Curr Opin Clin Nutr Metab Care. 2018;21:30-6.

5. Qiu J, Thapaliya S, Runkana A, Yang Y, Tsien C, Mohan ML, et al. Hyperammonemia in cirrhosis induces transcriptional regulation of myostatin by an NF-kappaB-mediated mechanism. Proc Natl Acad Sci U S A. 2013;110:18162-7.

6. Rangroo Thrane V, Thrane AS, Wang F, Cotrina ML, Smith NA, Chen M, et al. Ammonia triggers neuronal disinhibition and seizures by impairing astrocyte potassium buffering. Nat Med. 2013;19:1643-8.

7. Granados-Fuentes D, Cho K, Patti GJ, Costa R, Herzog ED, Montagnese S. Hyperammonaemia disrupts daily rhythms reversibly by elevating glutamate in the central circadian pacemaker. Liver Int. 2023;43:673-83.

8. Ono-Moore KD, Olfert IM, Rutkowsky JM, Chintapalli SV, Willis BJ, Blackburn ML, et al. Metabolic physiology and skeletal muscle phenotypes in male and female myoglobin knockout mice. Am J Physiol Endocrinol Metab. 2021;321:E63-E79.

9. Wu G, Anafi RC, Hughes ME, Kornacker K, Hogenesch JB. MetaCycle: an integrated R package to evaluate periodicity in large scale data. Bioinformatics. 2016;32:3351-3.

10. Glynn EF, Chen J, Mushegian AR. Detecting periodic patterns in unevenly spaced gene expression time series using Lomb-Scargle periodograms. Bioinformatics. 2006;22:310-6.

11. Hughes ME, Hogenesch JB, Kornacker K. JTK_CYCLE: an efficient nonparametric algorithm for detecting rhythmic components in genome-scale data sets. J Biol Rhythms. 2010;25:372-80.

12. Kumar A, Bellar A, Mishra S, Sekar J, Welch N, Dasarathy S. L-Isoleucine reverses hyperammonemia-induced myotube mitochondrial dysfunction and post-mitotic senescence. J Nutr Biochem. 2024;123:109498.

13. Mishra S, Welch N, Singh SS, Singh KD, Bellar A, Kumar A, et al. Ammonia transporter RhBG initiates downstream signaling and functional responses by activating NFkappaB. Proc Natl Acad Sci U S A. 2024;121:e2314760121.

14. Welch N, Mishra S, Bellar A, Kannan P, Gopan A, Goudarzi M, et al. Differential impact of sex on regulation of skeletal muscle mitochondrial function and protein homeostasis by hypoxia-inducible factor-1alpha in normoxia. J Physiol. 2024;602:2763-806.

15. Crowe AR, Yue W. Semi-quantitative Determination of Protein Expression using Immunohistochemistry Staining and Analysis: An Integrated Protocol. Bio Protoc. 2019;9:

16. Briguet A, Courdier-Fruh I, Foster M, Meier T, Magyar JP. Histological parameters for the quantitative assessment of muscular dystrophy in the mdx-mouse. Neuromuscul Disord. 2004;14:675-82.

17. Bellar A, Welch N, Dasarathy S. Exercise and physical activity in cirrhosis: opportunities or perils. J Appl Physiol (1985). 2020;128:1547-67.

18. Schneider CA, Rasband WS, Eliceiri KW. NIH Image to ImageJ: 25 years of image analysis. Nat Methods. 2012;9:671-5.

19. Singh SS, Kumar A, Welch N, Sekar J, Mishra S, Bellar A, et al. Multiomics-Identified Intervention to Restore Ethanol-Induced Dysregulated Proteostasis and Secondary Sarcopenia in Alcoholic Liver Disease. Cell Physiol Biochem. 2021;55:91-116.

20. Kumar A, Davuluri G, Welch N, Kim A, Gangadhariah M, Allawy A, et al. Oxidative stress mediates ethanol-induced skeletal muscle mitochondrial dysfunction and dysregulated protein synthesis and autophagy. Free Radic Biol Med. 2019;145:284-99.
