# Supplementary material for: Voluntary Activity Wheel Running Improves Hyperammonaemia‐Induced Skeletal Muscle Molecular and Metabolic Perturbations in Mice
Source: J Cachexia Sarcopenia Muscle. 2025 Aug 4;16(4):e70031. doi: 10.1002/jcsm.70031 (PMC12321975; doi:10.1002/jcsm.70031)
Supplement: Supplementary file 2 — Figure S1: Study design and organ weights. Studies were performed in male C57BL6/J mice aged 8–10 weeks treated with either sterile phosphate‐buffered saline (PBS, vehicle) or 2.5 mmol.kg−1.d−1 ammonia acetate (AmAc) for 42 days. 2 weeks post‐pump placement, mice were randomized to interventions (usual activity, UA, or voluntary wheel running, VWR) for 4 weeks. Grip strength and body weight were measured weekly. Metabolic and activity measurements were measured by the CLAMS metabolic cage system for 48 h, and body composition by EchoMRI prior to and post‐intervention. Mice were sacrificed at the end of the intervention, and tissues were harvested and weighed. Clinical lab parameters were measured. A. Schematic of study design. B. Lean body mass (top) and fat mass pre‐ and post‐intervention, and change in fat and lean mass and overall body weight. C. Average total, lean, and fat mass pre‐ to post‐intervention in different groups of mice. D. Percent lean and fat mass (measured by EchoMRI) as a proportion of total body weight. E. Plasma and skeletal muscle ammonia concentrations. F. Blood concentrations of alanine aminotransferase (ALT), aspartate aminotransferase (AST), blood urea nitrogen (BUN), glucose, and insulin. G. Representative photomicrographs and quantification of fibre typing by immunohistochemistry for Type I, IIA, IIB, and IIX fibres. Scale bar = 100um. H. Representative immunoblots and densitometry for Agrin protein. I. Organ weights. Average weights of organs in different groups. All data expressed as mean ± SD Statistical analysis : The one‐sample Kolmogorov–Smirnov test was performed to test if the data distribution was normal. Panels B and C. A paired t‐test was used for two‐group comparisons, while for multiple‐group comparisons, one‐way ANOVA followed by Tukey’s post hoc analysis was performed. Panel D. Blood ammonia concentrations, one‐way ANOVA followed by Tukey’s post hoc analysis was performed. For skeletal muscle ammonia concentrations, Kruska [file JCSM-16-e70031-s002.docx]

**Supplementary Figure Legends.**

**S. Fig. 1. Study design and organ weights.** Studies were performed in male C57BL6/J mice aged 8-10 weeks treated with either sterile phosphate-buffered saline (PBS, vehicle) or 2.5 mmol.kg^-1^.d^-1^ ammonia acetate (AmAc) for 42 days. 2 weeks post-pump placement, mice were randomized to interventions (usual activity, UA, or voluntary wheel running, VWR) for 4 weeks. Grip strength and body weight were measured weekly. Metabolic and activity measurements **were** measured by **the** CLAMS® metabolic cage system for 48 hours**,** **and** body composition by EchoMRI® prior to and post-intervention. Mice were sacrificed at the end of the intervention, and tissues were harvested and weighed. Clinical lab parameters were measured. **A.** Schematic of study design. **B. Lean body mass (top) and fat mass pre- and post-intervention, and change in fat and lean mass and overall body weight.** **C.** Average total, lean, and fat mass pre- to post-intervention in different groups of mice**.** **D.** Percent lean and fat mass (measured by EchoMRI®) as a proportion of total body weight. **E.** Plasma and skeletal muscle ammonia concentrations. **F.** Blood concentrations of alanine aminotransferase (ALT), aspartate aminotransferase (AST), blood urea nitrogen (BUN), glucose, and insulin**. G. Representative photomicrographs and quantification of fiber typing by immunohistochemistry for Type I, IIA, IIB, and IIX fibers. Scale bar = 100um. H. Representative immunoblots and densitometry for Agrin protein. I.** Organ weights. **Average weights of organs in different groups.** All data expressed as mean±SD**.**

***Statistical analysis***: **The one-sample Kolmogorov-Smirnov test was performed to test if the data distribution was normal. Panels B and C. A paired t-test was used for two-group comparisons, while for multiple-group comparisons, one-way ANOVA followed by Tukey’s post hoc analysis was performed. Panel D. Blood ammonia concentrations, one-way ANOVA followed by Tukey’s post hoc analysis was performed. For skeletal muscle ammonia concentrations, Kruskal-Wallis, followed by Dunn’s Multiple Comparison test, was performed. Panel E. Kruskal-Wallis followed by Dunn’s Multiple Comparison was performed for ALT, AST, and insulin; For BUN and glucose, one-way ANOVA followed by Tukey post-hoc test was used. Panels F-I, One-way ANOVA followed by Fisher uncorrected LSD was used for post hoc analysis.** * p<0.05; **p<0.01; *** p<0.001. n=8 in each group.

**S. Fig. 2. Average run distance and total activity in X-direction.** Studies were performed in male C57BL6/J mice aged 8-10 weeks treated with either sterile phosphate-buffered saline (PBS, vehicle) or 2.5 mmol.kg^-1^.d^-1^ ammonia acetate (AmAc) for 42 days. 2 weeks post-pump placement, mice were randomized to interventions (usual activity, UA, or voluntary wheel running, VWR) for 4 weeks. Total activity in the X**-** (length of the cage) **and Z- (height of the cage) directions** w**ere** measured in a CLAMS® metabolic cage by light beam breaks post-intervention (VWR or UA) **and total running distance in meters was measured during the 28 days of intervention**: **A. Violin plot of average run distance (meters) in the intervention groups of mice at week 1, week 2, week 3, and week 4. B.** **Average pre-intervention of total X-activity of all mice.** All data expressed as mean±SD**.**

***Statistical analysis***: **The Shapiro-Wilk test was performed to evaluate if the data distribution was normal. Since criteria for normality were not satisfied for these data, the Aligned Rank Transform (ART) for nonparametric two-way ANOVA analysis to determine the independent effect of: A. Treatment (PBS versus AmAc), time (weeks 1-4), and whether an interaction effect exists between treatment and time; B. Treatment and intervention (UA vs VWR), and whether an interaction effect exists between treatment and intervention. Post-hoc analysis was performed with Dunn’s test with Benjamini-Hochberg post-hoc correction.** * p<0.05; **p<0.01; *** p<0.001 unless otherwise stated. N=8 in each group.

**S. Fig. 3. Total activity in X-direction.** Studies were performed in male C57BL6/J mice aged 8-10 weeks treated with either sterile phosphate-buffered saline (PBS, vehicle) or 2.5 mmol.kg^-1^.d^-1^ ammonia acetate (AmAc) for 42 days. 2 weeks post-pump placement, mice were randomized to interventions (usual activity, UA, or voluntary wheel running, VWR) for 4 weeks. Total activity in the X**-**direction (length of the cage) was measured in a CLAMS® metabolic cage by light beam breaks pre- and post-intervention (VWR or UA). **A. Total X-activity post-intervention; B-E:** Average total X-activity pre- to post-intervention in: **B.** PBS-UA mice. **C.** AmAc-UA mice. **D.** PBS-VWR. **E**. AmAc-VWR mice. All data expressed as mean±SD**.**

***Statistical analysis*.** **For Panel A, the Shapiro-Wilk test was performed to evaluate if the data distribution was normal. Since criteria for normality were not satisfied for these data, the Aligned Rank Transform (ART) for nonparametric two-way ANOVA analysis to determine the independent effect of treatment (PBS versus AmAc), and intervention (UA vs VWR) and whether an interaction effect exists between treatment and intervention was used. Post-hoc analysis was performed using Dunn’s test with Benjamini-Hochberg post-hoc correction. Panels B-E. The one-sample Kolmogorov-Smirnov test was used to test if the data distribution was normal, followed by a paired t-test for pre-intervention to post-intervention data.** * p<0.05; **p<0.01; *** p<0.001 unless stated **otherwise**. N=8 in each group.

**S. Fig. 4. Ambulatory activity in the X-direction.** Studies were performed in male C57BL6/J mice aged 8-10 weeks treated with either sterile phosphate-buffered saline (PBS, vehicle) or 2.5 mmol.kg^-1^.d^-1^ ammonia acetate (AmAc) for 42 days. 2 weeks post-pump placement, mice were randomized to interventions (usual activity, UA, or voluntary wheel running, VWR) for 4 weeks. Ambulatory activity in the X-direction (length of the cage) was measured in a CLAMS® metabolic cage by light beam breaks pre- and post-intervention (VWR or UA). Average ambulatory X-activity pre-intervention of: **A.** All mice. **B-E:** Average ambulatory X-activity pre- to post-intervention in **B.** PBS-UA mice. **C.** AmAc-UA mice. **D.** PBS-VWR. **E**. AmAc-VWR mice. **F.** Bar graph showing ambulatory and non-ambulatory X-activity as a percent of total X-activity**.** All data expressed as mean±SD

***Statistical analysis*.** **For Panel A, the Shapiro-Wilk test was used to evaluate whether the data were normally distributed. Since the activity data were not normally distributed, the Aligned Rank Transform (ART) for nonparametric two-way ANOVA analysis to determine the independent effect of treatment (PBS versus AmAc), and intervention (UA vs VWR), and whether an interaction effect exists between treatment and intervention was used. Post-hoc analysis was performed using Dunn’s test with Benjamini-Hochberg post-hoc correction. Panels B-F. The one-sample Kolmogorov-Smirnov test was used to test if the data distribution was normal. Panels B-E, the paired t-test was performed for pre-intervention to post-intervention data. Panel F. One-way ANOVA followed by Tukey post-hoc test was used.** * p<0.05; **p<0.01; *** p<0.001 unless otherwise stated. N=8 in each group.

**S. Fig. 5. Total activity in the Z-direction.** Studies were performed in male C57BL6/J mice aged 8-10 weeks treated with either sterile phosphate-buffered saline (PBS, vehicle) or 2.5 mmol.kg^-1^.d^-1^ ammonia acetate (AmAc) for 42 days. 2 weeks post-pump placement, mice were randomized to interventions (usual activity, UA, or voluntary wheel running, VWR) for 4 weeks. Activity in the Z**-**direction (height of the cage) was measured in a CLAMS® metabolic cage by light beam breaks pre- and post-intervention (VWR or UA). Average **total** Z-activity of **A. P**re-intervention**;** **B. Post-intervention comparing PBS-UA/AmAc-UA; C-F:** Average Z-activity pre- to post-intervention in **C.** PBS-UA mice. **D.** AmAc-UA mice. **E.** PBS-VWR. **F**. AmAc-VWR mice**;** **G.** Line graph showing **zoomed views** **activity at** time 334 to 720 and 1440 to 1800 minutes to highlight observed differences in Z-activity, with bar graphs representing each of the 18 minute time points between the two groups or the average of all time points between 334 to 720 and 2800 **to** 1440 minutes. All data expressed as mean**±SD.** ***Statistical analysis*. For Panels A and B, the Shapiro-Wilk test was used to evaluate if the data were normally distributed. Since the activity data were not normally distributed, the Aligned Rank Transform (ART) for nonparametric two-way ANOVA analysis to determine the independent effect of treatment (PBS versus AmAc), and intervention (UA vs VWR), and whether an interaction effect exists between treatment and intervention was used. Post-hoc analysis was performed using Dunn’s test with Benjamini-Hochberg post-hoc correction. Panels C-G. The one-sample Kolmogorov-Smirnov test was used to determine if the data distribution was normal. For Panels C-F, pre-intervention to post-intervention data, the paired t-test was performed. Panel G. Unpaired t-test for independent groups. * p<0.05; **p<0.01; *** p<0.001 unless otherwise stated. N=8 in each group.**

**S. Fig. 6. Correlation of Activity Data.** Studies were performed in male C57BL6/J mice aged 8-10 weeks treated with either sterile phosphate-buffered saline (PBS, vehicle) or 2.5 mmol.kg^-1^.d^-1^ ammonia acetate (AmAc) for 42 days. 2 weeks post-pump placement, mice were randomized to interventions (usual activity, UA, or voluntary wheel running, VWR) for 4 weeks. Total activity in the X-direction (length of the cage), ambulatory activity in the X-direction (length of the cage), and activity in the Z-direction (height of the cage) were measured in a CLAMS® metabolic cage by light beam breaks post-intervention (VWR or UA). The number of wheel rotations measured total wheel rotations, and the zeitgeber time (ZT) of rotations was measured over the 28-day intervention period. **A-D** Correlation plot of total X-activity to ambulatory **X**-activity, total **X**-activity to **Z**-activity and ambulatory **X**-activity to **Z**-activity in: **A.** PBS-UA mice. **B.** AmAc-UA mice. **C.** PBS-VWR mice. **D**. AmAc-VWR mice. **E,F** Correlation plot of wheel running activity (in VWR mice only) to total x-activity, ambulatory **X**-activity and **Z**-activity in**. E**. PBS-VWR mice. **F.** AmAc-VWR mice.

***Statistical analyses*.** All data expressed as **a** correlation plot of XY data: Pearson’s correlation analysis with r value calculation.*p<0.05; **p<0.01; ***p<0.001 unless otherwise stated. N=8 in each group.

**S. Fig. 7. Wheel contact activity during hyperammonemia. Studies were performed in male C57BL6/J mice aged 8-10 weeks treated with either sterile phosphate-buffered saline (PBS, vehicle) or 2.5 mmol.kg^-1^.d^-1^ ammonia acetate (AmAc) for 42 days. 2 weeks post-pump placement, mice were randomized to interventions (usual activity, UA, or voluntary wheel running, VWR) for 4 weeks. The distance in meters and the zeitgeber time (ZT) of rotations were measured over the 28-day intervention period. Hourly meters run for VWR mice (n=6 in each group) for 10 of the 28 days of the intervention period (left) with double-plotted averages for 6 mice over 40 hours of running measurements (right).**

***Statistical analyses*. The one-sample Kolmogorov-Smirnov test was used to test for normality. Since the data were not normally distributed, the 2-sample Kolmogorov-Smirnov test was used for comparisons. * p<0.05; **p<0.01; *** p<0.001 unless stated within the figure. N=8 mice in each group.**

**S. Fig. 8. Circadian patterns of wheel rotations. Studies were performed in male C57BL6/J mice aged 8-10 weeks, treated with either sterile phosphate-buffered saline (PBS, vehicle) or 2.5 mmol.kg^-1^.d^-1^ ammonia acetate (AmAc) for 42 days. 2 weeks post-pump placement, mice were randomized to interventions (usual activity, UA, or voluntary wheel running, VWR) for 4 weeks. A. Hourly wheel rotations for VWR mice (n=6 in each group) for 10 of the 28 days of the intervention period B. Average running distance per hours 10-18 during the dark cycle of days 3-5 and 10-11 with peak running time noted on each day showing the difference of in running activity between the two groups. C. Average wheel rotations per hour 0-9 during the light cycle on days 5-7, showing the difference in running activity between the two groups. D. Average rotations during the dark and light cycle between the two groups of mice. Gray bars on the line graphs represent the dark cycle, and the white background represents the light cycle. All data expressed as mean±SD.**

***Statistical analysis*: The one-sample Kolmogorov-Smirnov test for normality was used. Panels B-D. Unpaired t-test with Welch’s correction was used. * p<0.05; **p<0.01; *** p<0.001 unless stated within the figure. N=8 mice in each group.**

**S. Fig. 9. Circadian patterns of distance run. Studies were performed in male C57BL6/J mice aged 8-10 weeks treated with either sterile phosphate-buffered saline (PBS, vehicle) or 2.5 mmol.kg^-1^.d^-1^ ammonia acetate (AmAc) for 42 days. 2 weeks post-pump placement, mice were randomized to interventions (usual activity, UA, or voluntary wheel running, VWR) for 4 weeks. A. Hourly meters run for VWR mice (n=6 in each group) for 10 of the 28 days of the intervention period B. Average meters run per hours 10-18 during the dark cycle of days 3-5 and 10-11 with peak running time noted on each day showing the difference of in running activity between the two groups. C. Average meters run per hour 0-9 during the light cycle on days 5-7, showing the difference in running activity between the two groups. D. Average meters run during the dark and light cycle between the two groups of mice. Gray bars on the line graphs represent the dark cycle and the white background represents light cycle. All data expressed as mean±SD.**

***Statistical analysis*. The one-sample Kolmogorov-Smirnov test for normality was used. Panels B-D. An unpaired t-test with Welch’s correction was used. * p<0.05; **p<0.01; *** p<0.001 unless stated within the figure. N=8 mice in each group.**

**S. Fig. 10. Circadian patterns of post-intervention X- and Z-activity.** Studies were performed in male C57BL6/J mice aged 8-10 weeks treated with either sterile phosphate-buffered saline (PBS, vehicle) or 2.5 mmol.kg^-1^.d^-1^ ammonia acetate (AmAc) for 42 days. 2 weeks post-pump placement, mice were randomized to interventions (usual activity, UA, voluntary wheel running, VWR) for 4 weeks. Total and ambulatory activity in the X direction (length of the cage) and Z-activity (height of the cage) w measured in a CLAMS® metabolic cage by light beam breaks post-intervention (VWR or UA): **A.** Total X-activity post-intervention as a line graph over time and bar graphs showing total **X**-activity in the light and dark cycle between each group and the average of light/dark cycle in all 4 groups of mice. **B.** Ambulatory X-activity post-intervention as a line graph over time and bar graphs showing ambulatory **X**-activity in the light and dark cycle between each group and the average of light/dark cycle in all 4 groups of mice. **C.** Z-activity post-intervention as a line graph over time and bar graphs showing **Z**-activity in the light and dark cycle between each group and the average of light/dark cycle in all 4 groups of mice. Gray bars on the line graphs represent dark cycle and white background represents light cycle. All data expressed as mean±SD**.**

***Statistical analysis*.** **The one-sample Kolmogorov-Smirnov test for normality was used. For Total and ambulatory X-activity, the** Student’s **t**-test for two groups**,** and **for four groups,** one-way ANOVA followed by **Fisher’s least significant difference was used. For total Z-activity for two groups, an unpaired t-test was performed, for four groups comparison, Kruskal-Wallis analysis followed by Dunn’s multiple comparison test was performed.** *p<0.05; **p<0.01; ***p<0.001 unless otherwise stated. N=8 in each group.

**S. Fig. 11. Effect of treatment/intervention on VO_2_.** Studies were performed in male C57BL6/J mice aged 8-10 weeks treated with either sterile phosphate buffered saline (PBS, vehicle) or 2.5 mmol.kg^-1^.d^-1^ ammonia acetate (AmAc) for 42 days. 2 weeks post-pump placement, mice were randomized to interventions (usual activity, UA, or voluntary wheel running, VWR) for 4 weeks. Volume of oxygen inhaled (VO_2_) was measured in a CLAMS® metabolic cage pre- and post-intervention (VWR or UA). **A.** Average VO_2_ pre-intervention in all mice. **B. VO_2_ post-intervention in all mice. C-F** Pre- to post- intervention VO_2_ in **C.** PBS-UA mice. **D.** AmAc-UA mice. **E.** PBS-VWR. **F**. AmAc-VWR mice. All data expressed as mean±SD**.**

***Statistical analysis*.** **Panels A,B: Based on the Shapiro-Wilk test, the metabolic data distribution was not normal. The Aligned Rank Transform (ART) for nonparametric two-way ANOVA analysis was used to determine the independent effect of treatment (PBS versus AmAc), and intervention (UA vs VWR), and whether an interaction effect exists between treatment and intervention. Post-hoc analysis was performed using Dunn’s test with Benjamini-Hochberg post-hoc correction. For pre- to post-intervention data, a one sample Kolmogorov-Smirnov test for normality was followed by a paired t-test (panels C,D, and F). For panel E, the Wilcoxon signed-rank test was used.** *p<0.05; **p<0.01; ***p<0.001 unless otherwise stated. N=8 in each group.

**S. Fig. 12. Effect of treatment/intervention on VCO_2_.** Studies were performed in male C57BL6/J mice aged 8-10 weeks treated with either sterile phosphate buffered saline (PBS, vehicle) or 2.5 mmol.kg^-1^.d^-1^ ammonia acetate (AmAc) for 42 days. 2 weeks post-pump placement, mice were randomized to interventions (usual activity, UA, or voluntary wheel running, VWR) for 4 weeks. Volume of carbon dioxide exhaled (VCO_2_) was measured in a CLAMS® metabolic cage pre- and post-intervention (VWR or UA). **A.** Pre-intervention VCO_2_ of all mice. **B. VCO_2_ post-intervention in all mice. C-F:** Pre- to post-intervention VCO_2_ in **C.** PBS-UA mice. **D.** AmAc-UA mice. **E.** PBS-VWR. **F**. AmAc-VWR mice. All data expressed as mean±SD**.**

***Statistical analysis*.** **Panels A,B: Based on the Shapiro-Wilk test, the metabolic data distribution was not normal. The Aligned Rank Transform (ART) for nonparametric two-way ANOVA analysis was used to determine the independent effect of treatment (PBS versus AmAc), and intervention (UA vs VWR), and whether an interaction effect exists between treatment and intervention. Post-hoc analysis was performed with Dunn’s test with Benjamini-Hochberg post-hoc correction. For pre- to post-intervention data, a Kolmogorov-Smirnov test for normality distribution was followed by a paired t-test (panels C-F). *p<0.05; **p<0.01; ***p<0.001 unless otherwise stated. N=8 in each group.**

**S. Fig. 13. Effect of treatment/intervention on respiratory exchange ratio (RER).** Studies were performed in male C57BL6/J mice aged 8-10 weeks treated with either sterile phosphate buffered saline (PBS, vehicle) or 2.5 mmol.kg^-1^.d^-1^ ammonia acetate (AmAc) for 42 days. 2 weeks post-pump placement, mice were randomized to interventions (usual activity, UA, or voluntary wheel running, VWR) for 4 weeks. Respiratory exchange ratio (RER) was measured in a CLAMS® metabolic cage pre- and post-intervention (VWR or UA). **A.** Average RER of all mice pre-intervention. **B. Average RER of all mice post-intervention. C-F** Pre- to post-intervention RER in **C.** PBS-UA mice. **D.** AmAc-UA mice. **E.** PBS-VWR. **F**. AmAc-VWR mice. All data expressed as mean±SD ***Statistical analysis*.** **Panels A,B: Based on the Shapiro-Wilk test, the metabolic data distribution was not normal. The Aligned Rank Transform (ART) for nonparametric two-way ANOVA analysis was used to determine the independent effect of treatment (PBS versus AmAc), and intervention (UA vs VWR), and whether an interaction effect exists between treatment and intervention. Post-hoc analysis was performed with Dunn’s test with Benjamini-Hochberg post-hoc correction. For pre- to post-intervention data, a Kolmogorov-Smirnov test for normality distribution was followed by a paired t-test (panels C-F). *p<0.05; **p<0.01; ***p<0.001 unless otherwise stated. N=8 in each group.**

**S. Fig. 14. Circadian patterns of post-intervention metabolic measures.** Studies were performed in male C57BL6/J mice aged 8-10 weeks treated with either sterile phosphate-buffered saline (PBS, vehicle) or 2.5 mmol.kg^-1^.d^-1^ ammonia acetate (AmAc) for 42 days. 2 weeks post-pump placement, mice were randomized to interventions (usual activity, UA, or voluntary wheel running, VWR) for 4 weeks. Volume of oxygen inhaled (VO_2_)_,_ volume of carbon dioxide exhaled (VCO_2_), and respiratory exchange ratio (RER) was measured in a CLAMS® metabolic cage post-intervention (VWR or UA): **A.** VO_2,_ post-intervention as a line graph over time and bar graphs showing total VO_2_ in the light and dark cycle between each group and the average of light/dark cycle in all 4 groups of mice. **B.** VCO_2_ post-intervention as a line graph over time and bar graphs showing VCO_2_ in the light and dark cycle between each group and the average of light/dark cycle in all 4 groups of mice. **C.** RER post-intervention as a line graph over time and bar graphs showing RER in the light and dark cycle between each group and the average of light/dark cycle in all 4 groups of mice. Gray bars on the line graphs represent the dark cycle, and the white background represents the light cycle. All data expressed as mean±SD**.**

***Statistical analysis*.** **The one-sample Kolmogorov-Smirnov test for normality was used. For two-group comparisons, the Student’s t-test, and for four-group comparisons, one-way ANOVA followed by Fisher’s least significant difference was used. For comparisons of light-dark cycle in the AmAc-UA group in Panel A and PBS-VWR group (Panel C), a Mann-Whitney test was used.*** p<0.05; **p<0.01; *** p<0.001 unless otherwise stated. N=8 in each group.

**S. Fig. 15. Pre-intervention energy expenditure (EE). Studies were performed in male C57BL6/J mice aged 8-10 weeks treated with either sterile phosphate-buffered saline (PBS, vehicle) or 2.5 mmol.kg^-1^.d^-1^ ammonia acetate (AmAc) for 42 days. 2 weeks post-pump placement, mice were later randomized to interventions (usual activity, UA, or voluntary wheel running, VWR) for 4 weeks. Energy Expenditure (EE) was measured in in a CLAMS® metabolic cage). Average EE of all mice pre-intervention is shown. All data expressed as mean±SD.**

***Statistical Analyses*. Based on the Shapiro-Wilk normality test, EE data were not normally distributed. Therefore, we used the Aligned Rank Transform (ART) for nonparametric two-way ANOVA analysis to determine the independent effect of treatment (PBS versus AmAc), and intervention (UA vs VWR), and whether an interaction effect exists between treatment and intervention. Post-hoc analysis was performed with Dunn’s test with Benjamini-Hochberg post-hoc correction. .* p<0.05; **p<0.01; *** p<0.001 unless otherwise stated. N=8 in each group.**

**S. Fig. 16. Post-intervention energy expenditure (EE).** Studies were performed in male C57BL6/J mice aged 8-10 weeks treated with either sterile phosphate buffered saline (PBS, vehicle) or 2.5 mmol.kg^-1^.d^-1^ ammonia acetate (AmAc) for 42 days. 2 weeks post-pump placement, mice were randomized to interventions (usual activity, UA, or voluntary wheel running, VWR) for 4 weeks. Energy Expenditure (EE) was measured in a CLAMS® metabolic cage **post-**intervention (VWR or UA). **A.** Average EE . **B. Total**  EE . All data expressed as mean±SD**.**

***Statistical analys*is.** **For Panels A,B, Shapiro-Wilk normality test showed that the data were not normally distributed. The Aligned Rank Transform (ART) for nonparametric two-way ANOVA analysis to determine the independent effect of treatment (PBS versus AmAc), and intervention (UA vs VWR), and whether an interaction effect exists between treatment and intervention. Post-hoc analysis was performed using Dunn’s test with Benjamini-Hochberg post-hoc correction.** * p<0.05; **p<0.01; *** p<0.001 unless otherwise stated. N=8 in each group.

**S. Fig. 17. Effect of treatment/intervention on energy expenditure.** Studies were performed in male C57BL6/J mice aged 8-10 weeks treated with either sterile phosphate-buffered saline (PBS, vehicle) or 2.5 mmol.kg^-1^.d^-1^ ammonia acetate (AmAc) for 42 days. 2 weeks post-pump placement, mice were randomized to interventions (usual activity, UA, or voluntary wheel running, VWR) for 4 weeks. Energy Expenditure (EE) was measured in a CLAMS® metabolic cage pre- and post-intervention (VWR or UA). **A-D** Pre- to post-intervention EE in **A.** PBS-UA mice. **B.** AmAc-UA mice. **C.** PBS-VWR. **D**. AmAc-VWR mice.

All data expressed as mean±SD**.**

***Statistical analysis*.** **The Kolmogorov-Smirnov test for normality distribution was followed by a paired t-test For pre- to post-intervention data,.** * p<0.05; **p<0.01; *** p<0.001 unless otherwise stated. N=8 in each group.

**S. Fig. 18. Circadian patterns of post-intervention energy expenditure.** All studies were conducted in **8–10-week-old** male C57BL6/J mice infused either with vehicle (phosphate-buffered saline; PBS) or 2.5 mmol/kg.d^-1^ ammonium acetate (AmAc) for 6 weeks. After the initial 2 weeks, mice were randomized to either usual activity (UA) or voluntary wheel running (VWR) for 28 days. EE and the zeitgeber time (ZT) were recorded over the 48 hours of data collection. Additionally, the amplitude and phase of the peaks were recorded **A.** EE of mice post-intervention and bar graphs showing the amplitude and Phase. **B.** EE post-intervention as **a** line graph over time and bar graphs showing EE in the light and dark cycle between each group and the average light/dark cycle in all 4 groups of mice. All data expressed as mean±SD

***Statistical analysis.*  The one-sample Kolmogorov-Smirnov test for normality was used to evaluate whether the data distribution was normal. For circadian data in Panel A, the phase of peaks of data was analyzed with one-way ANOVA, followed by Tukey’s multiple comparison test, and the line graph and amplitude data were analyzed using the Kruskal-Wallis test, followed by Dunn’s multiple comparison test. For panel B,** **an unpaired Student’s t-test was used for 2 groups, and for 4 groups, a one-way ANOVA followed by Fisher’s least significant difference post-hoc test was used.** * p<0.05; **p<0.01; *** p<0.001 unless otherwise stated within the figure. N=8 in each group.

**S. Fig. 19. Effect of treatment/intervention on food intake.** Studies were performed in male C57BL6/J mice aged 8-10 weeks treated with either sterile phosphate buffered saline (PBS, vehicle) or 2.5 mmol.kg^-1^.d^-1^ ammoni**um** acetate (AmAc) for 42 days. 2 weeks post-pump placement, mice were randomized to interventions (usual activity, UA, or voluntary wheel running, VWR) for 4 weeks. Food intake was measured in a CLAMS® metabolic cage pre- and post-intervention (VWR or UA). **A. Pre-intervention food measurements**. **B.** P**ost**-intervention food measurements. **C.** Pre**-** vs post-intervention total food intake. **D.** Pre**-** vs post-intervention lowest food intake. **E.** Pre**-** vs post-intervention highest food intake. **F.** Pre**-** vs post-intervention average food intake. **G. pre- vs.** post-intervention number of food grabs. All Data expressed as mean±SD. ***Statistical analyses*. The one-sample Kolmogorov-Smirnov test was performed to evaluate whether the data distribution was normal.** For Panel**s** A and B: **One**-way ANOVA followed by Tukey’s post hoc analysis **for normally distributed data (pre-intervention total and highest food intake, average food obtained, number of food removal activities)**. **All other data were not normally distributed. Hence, a Kruskal-Wallis test followed by Dunn’s multiple comparisons was used.** For Panel C-G: Student’s paired t-test for pr**e-** to post**-**analysis **(except for the data in Panel D, and in Panel F, PBS-UA where data were not normally distributed and a Wilcoxon’s signed-rank test was used)**. * p<0.05; **p<0.01; *** p<0.001. n=8 in each group.

**S.Fig. 20. Mitochondrial mass did not change with hyperammonemia or voluntary wheel running**. **Representative immunoblots and densitometry of citrate synthase (CS) and voltage-dependent anion channel (VDAC). β-Actin, used as a loading control, was run on the same membrane as CS and VDAC.**

***Statistical analyses*.** **The one-sample Kolmogorov-Smirnov test was performed to evaluate whether the data distribution was normal. One-way ANOVA followed by Tukey’s post hoc analysis. N=8 mice for each group. * p<0.05; **p<0.01; *** p<0.001.**

**S. Fig. 21. Correlation of Wheel Running Data with other outcomes. Studies were performed in male C57BL6/J mice aged 8-10 weeks treated with either sterile phosphate-buffered saline (PBS, vehicle) or 2.5 mmol.kg^-1^.d^-1^ ammonium acetate (AmAc) for 42 days. 2 weeks post-pump placement, mice were randomized to interventions (usual activity, UA, or voluntary wheel running, VWR) for 4 weeks. The number of wheel rotations, distance run in meters, and the zeitgeber time (ZT) of rotations were measured over the 28-day intervention period. In addition, gastrocnemius muscle weight, grip strength, protein expression of electron transport chain complex I and II, Puromycin incorporation, phosphorylated mTOR (pmTOR), phosphorylated S6 Kinase (pS6), phosphorylated eif2α (peif2α), plasma ammonia concentration, skeletal muscle ammonia concentration, expression of glutamine synthase (GS) and aspartate aminotransferase (AST) and mitochondrial function responses to adenosine diphosphate (ADP), glutamate, and succinate were measured at the end of the study. A,B Correlation plot of running distance, gastrocnemius muscle weight, grip strength, protein expression of electron transport chain complex I and II, Puromycin incorporation, pmTOR, pS6, peif2α, plasma ammonia concentration, skeletal muscle ammonia concentration, expression of GS and AST as well as mitochondrial functional responses to ADP, glutamate, and succinate A. PBS-VWR mice. B. AmAc-VWR mice. All data expressed as a correlation plot of XY data:**

***Statistical analyses*. Pearson’s correlation analysis with r-values provided. *p<0.05; **p<0.01; ***p<0.001 unless otherwise stated. N=8 in each group.**
