# Supplementary material for: Voluntary Activity Wheel Running Improves Hyperammonaemia‐Induced Skeletal Muscle Molecular and Metabolic Perturbations in Mice
Source: J Cachexia Sarcopenia Muscle. 2025 Aug 4;16(4):e70031. doi: 10.1002/jcsm.70031 (PMC12321975; doi:10.1002/jcsm.70031)
Supplement: Supplementary file 3 — Data S2: Supplementary Figures. [file JCSM-16-e70031-s001.pdf]

S. Figure 1

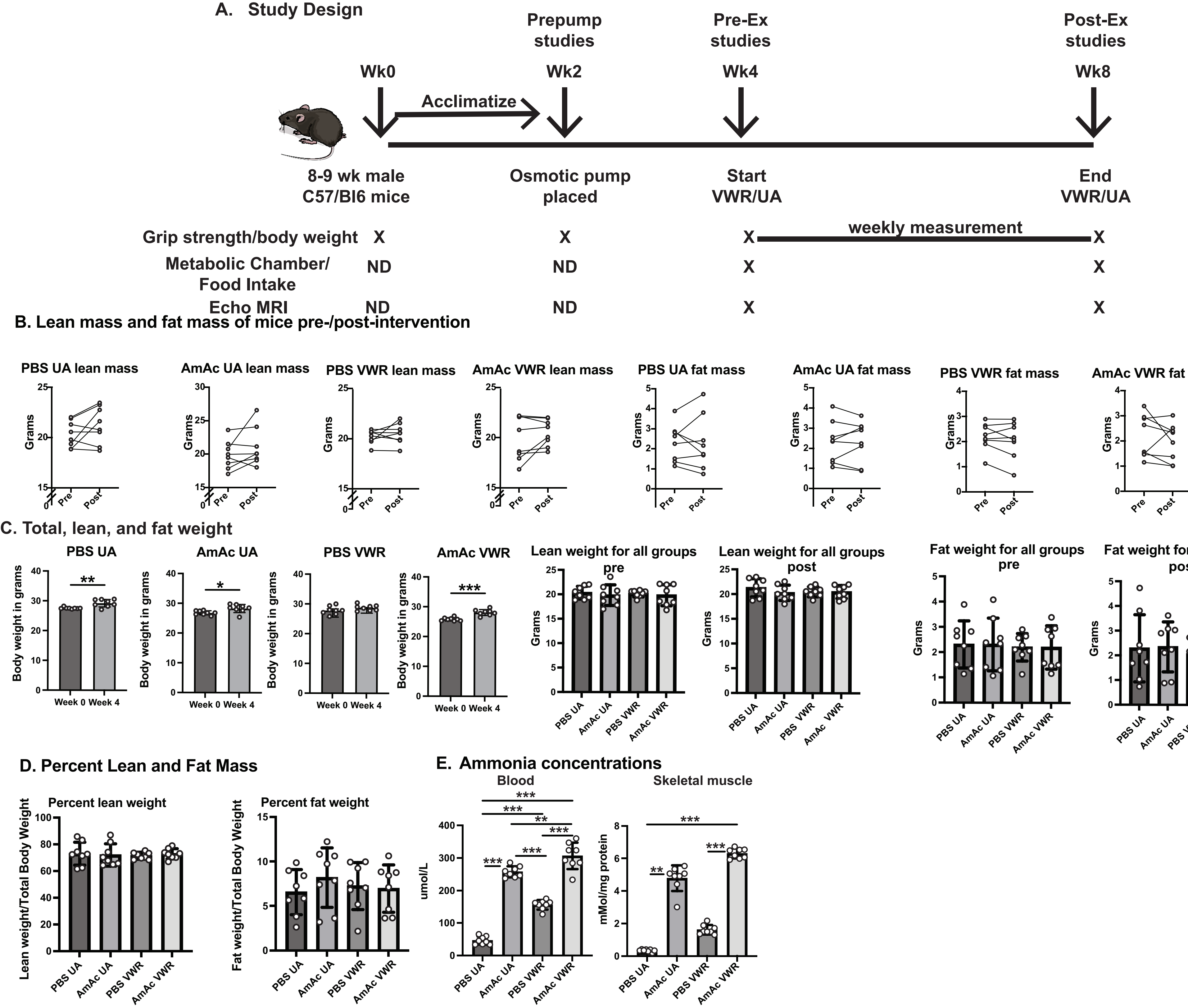
A. Study Design
Prepump studies
Pre-Ex studies
Post-Ex studies
Wk0
Wk2
Wk4
Wk8
Acclimatize
8-9 wk male C57/Bl6 mice
Osmotic pump placed
Start VWR/UA
End VWR/UA
Grip strength/body weight
X
X
X
weekly measurement
X
Metabolic Chamber/ Food Intake
ND
ND
X
X
Echo MRI
ND
ND
X
X
B. Lean mass and fat mass of mice pre-/post-intervention
PBS UA lean mass
AmAc UA lean mass
PBS VWR lean mass
AmAc VWR lean mass
PBS UA fat mass
AmAc UA fat mass
PBS VWR fat mass
AmAc VWR fat mass
Grams
Pre
Post
C. Total, lean, and fat weight
PBS UA
AmAc UA
PBS VWR
AmAc VWR
Body weight in grams
Week 0
Week 4
Lean weight for all groups pre
Lean weight for all groups post
Fat weight for all groups pre
Fat weight for all groups post
D. Percent Lean and Fat Mass
Percent lean weight
Lean weight/Total Body Weight
Percent fat weight
Fat weight/Total Body Weight
E. Ammonia concentrations
Blood
umol/L
Skeletal muscle
mMol/mg protein
PBS UA
AmAc UA
PBS VWR
AmAc VWR

S. Figure 1

F. Blood concentrations

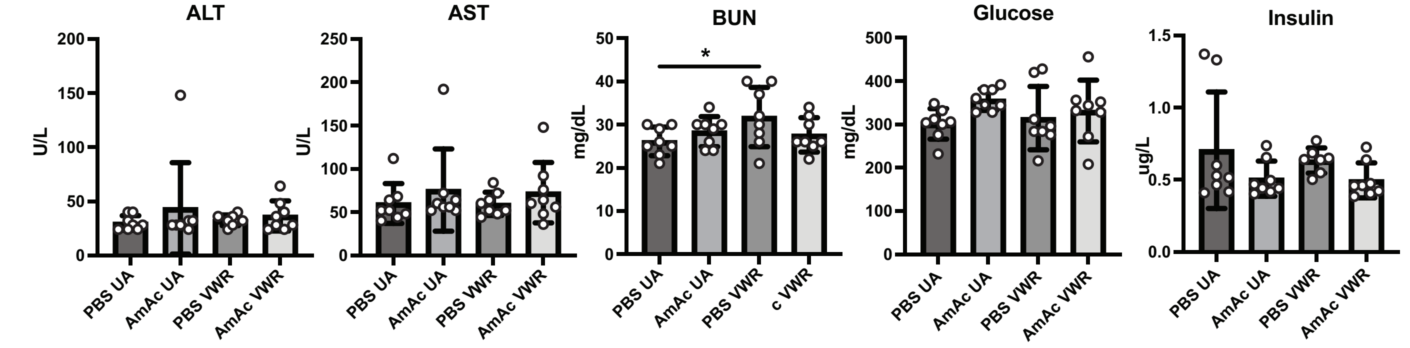

G. Fiber Type Data

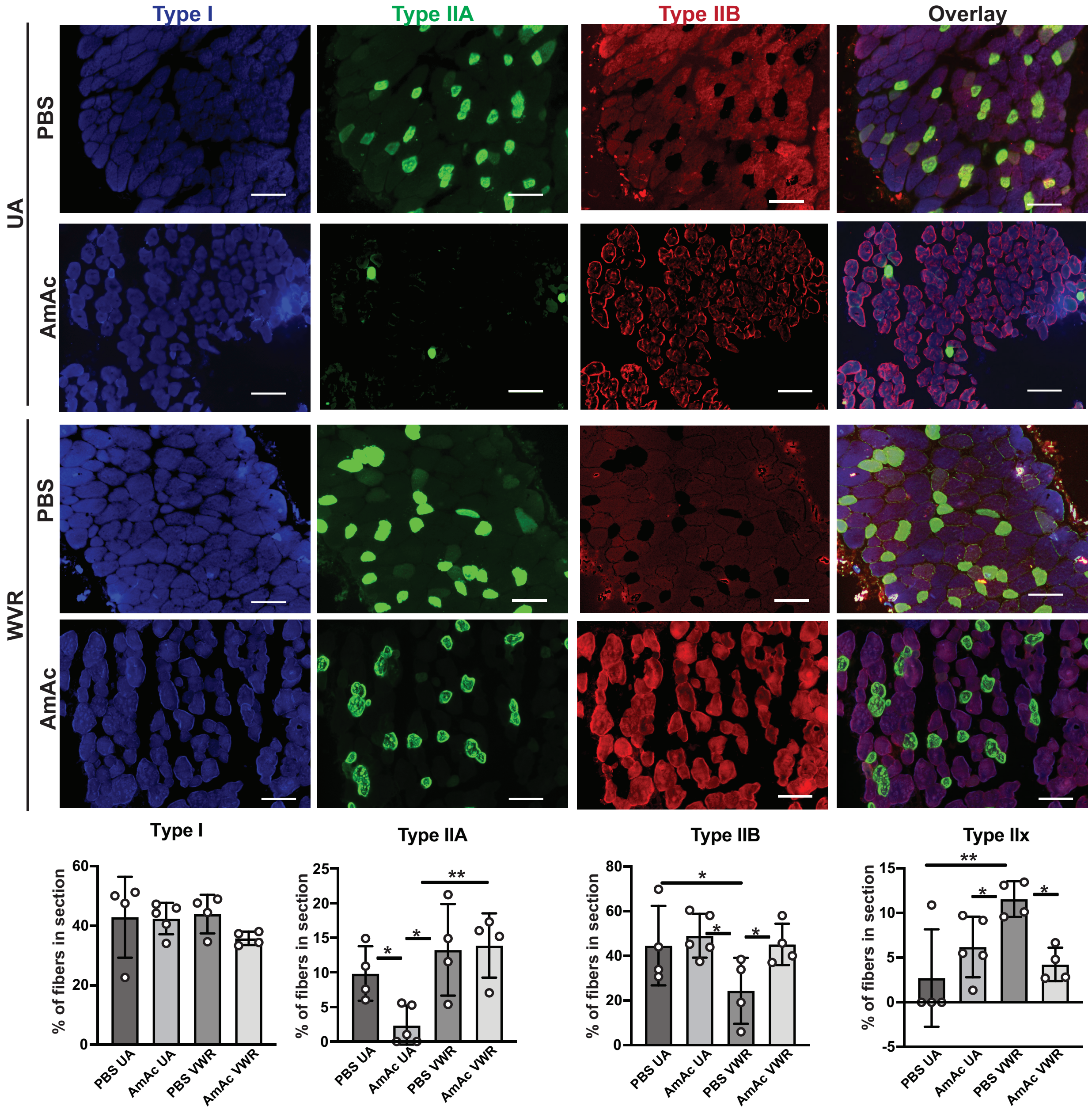

H. Agrin Expression

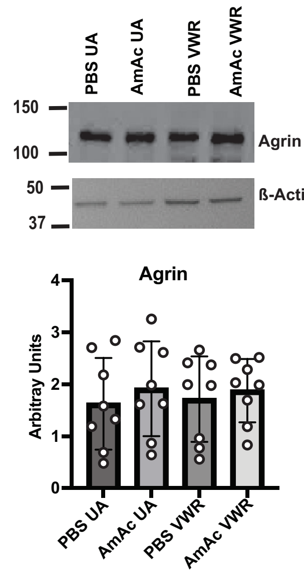

I. Organ weight

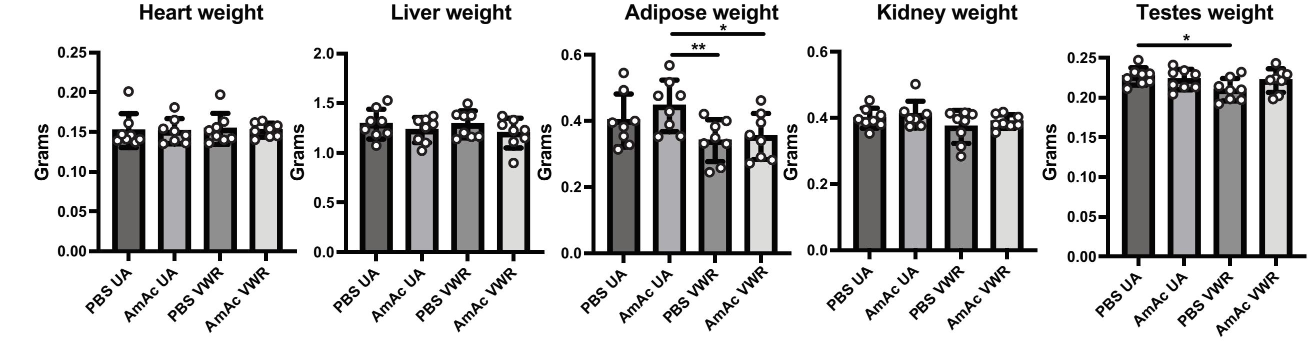

S. Figure 2

A. Average Run Distance

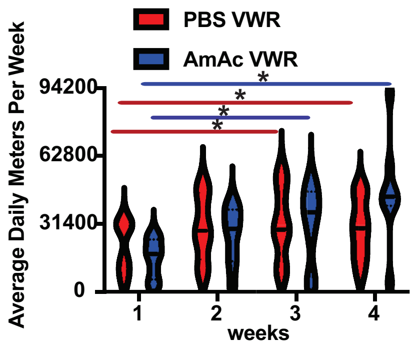

B. Total X-activity pre-intervention

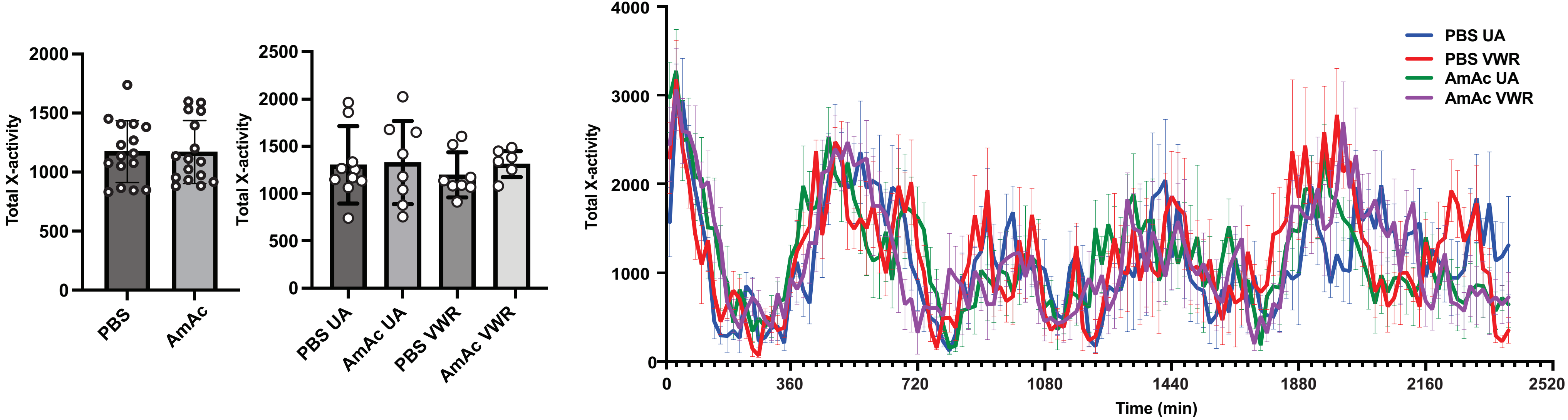

S. Figure 3

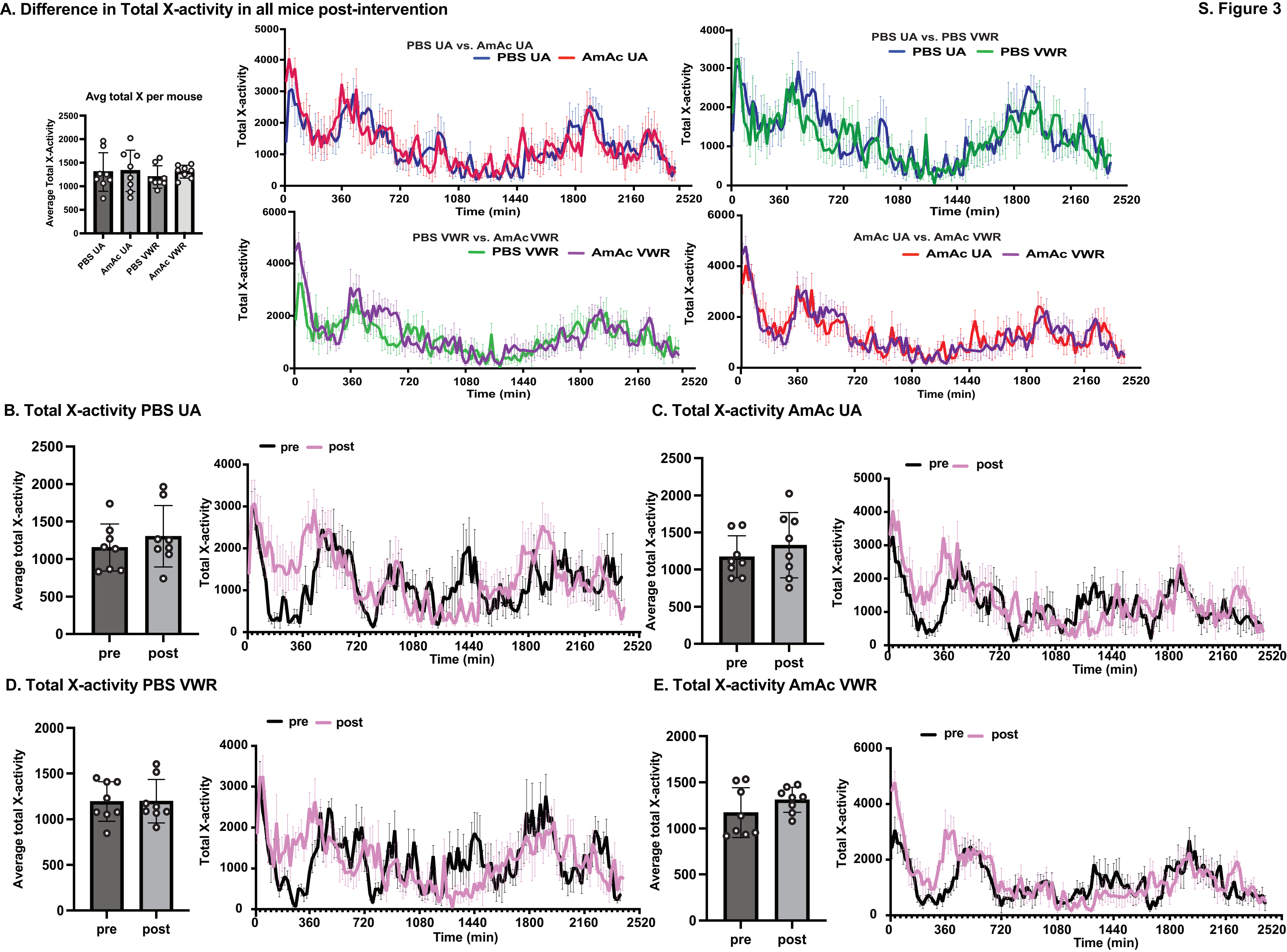
A. Difference in Total X-activity in all mice post-intervention
Avg total X per mouse
Average Total X-Activity
PBS UA
AmAc UA
PBS VWR
AmAc VWR
PBS UA vs. AmAc UA
PBS UA
AmAc UA
PBS UA vs. PBS VWR
PBS VWR
PBS VWR vs. AmAc VWR
AmAc VWR
AmAc UA vs. AmAc VWR
Total X-activity
Time (min)
B. Total X-activity PBS UA
C. Total X-activity AmAc UA
D. Total X-activity PBS VWR
E. Total X-activity AmAc VWR
Average total X-activity
pre
post

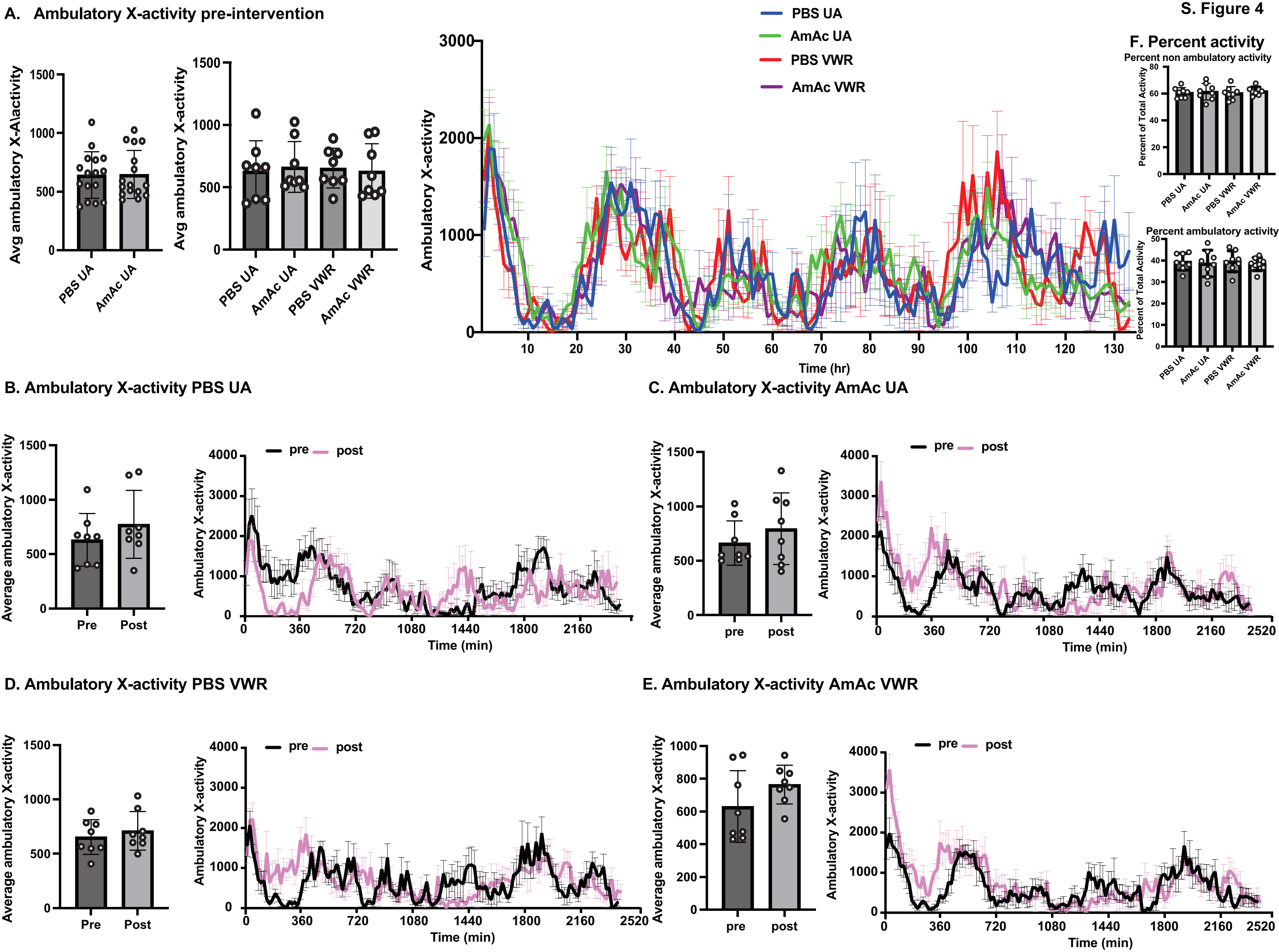
S. Figure 4
A. Ambulatory X-activity pre-intervention
Avg ambulatory X-Alactivity
Avg ambulatory X-activity
PBS UA
AmAc UA
PBS VWR
AmAc VWR
Ambulatory X-activity
Time (hr)
F. Percent activity
Percent non ambulatory activity
Percent ambulatory activity
Percent of Total Activity
B. Ambulatory X-activity PBS UA
C. Ambulatory X-activity AmAc UA
D. Ambulatory X-activity PBS VWR
E. Ambulatory X-activity AmAc VWR
Average ambulatory X-activity
Pre
Post
pre
post
Time (min)

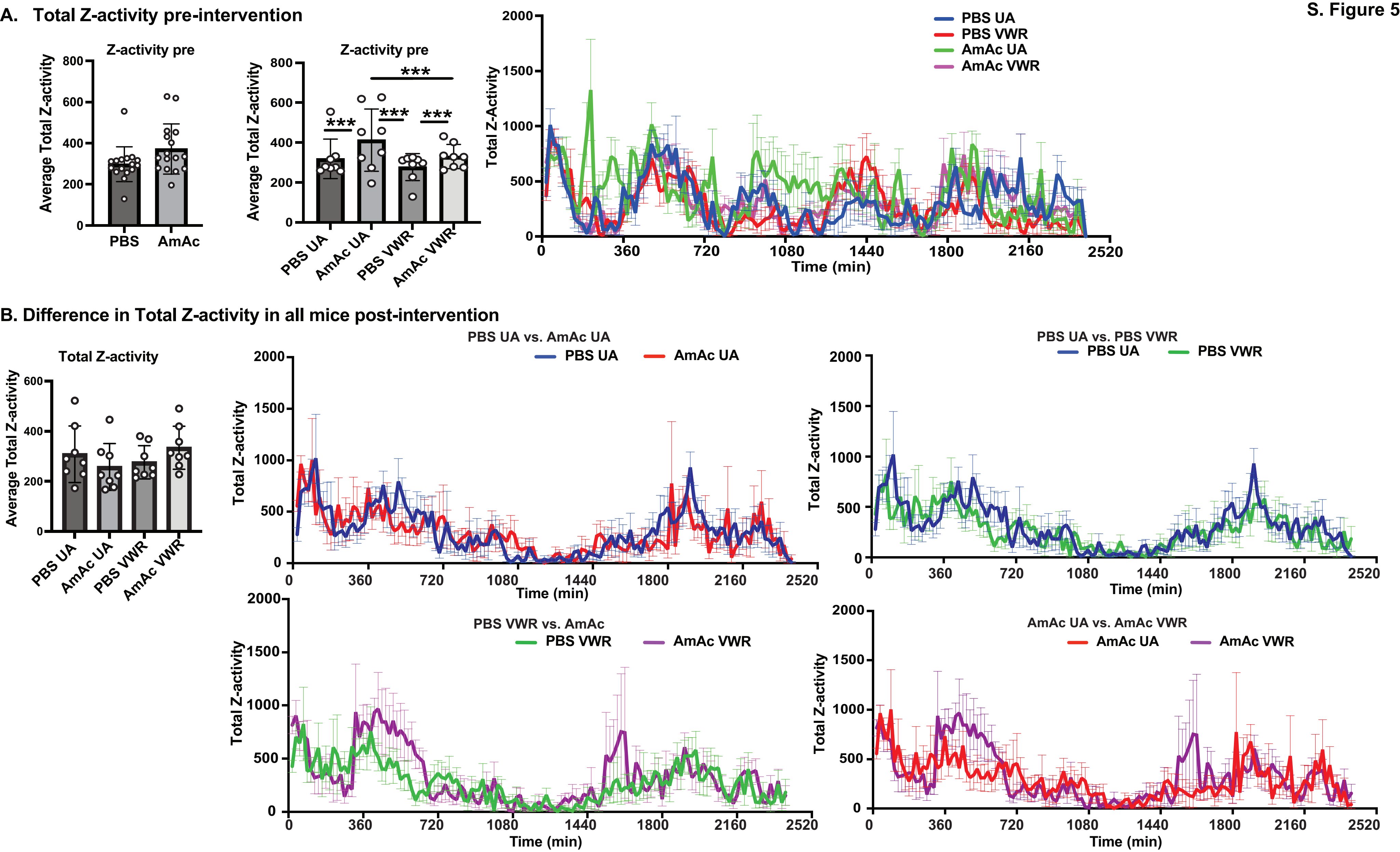
S. Figure 5
A. Total Z-activity pre-intervention
Z-activity pre
Average Total Z-activity
800
600
400
200
0
PBS
AmAc
Z-activity pre
***
***
***
***
PBS UA
AmAc UA
PBS VWR
AmAc VWR
2000
1500
1000
500
0
Total Z-Activity
0
360
720
1080
1440
1800
2160
2520
Time (min)
PBS UA
PBS VWR
AmAc UA
AmAc VWR
B. Difference in Total Z-activity in all mice post-intervention
Total Z-activity
Average Total Z-activity
600
400
200
0
PBS UA
AmAc UA
PBS VWR
AmAc VWR
PBS UA vs. AmAc UA
PBS UA
AmAc UA
Total Z-activity
Time (min)
PBS UA vs. PBS VWR
PBS UA
PBS VWR
Total Z-activity
Time (min)
PBS VWR vs. AmAc
PBS VWR
AmAc VWR
Total Z-activity
Time (min)
AmAc UA vs. AmAc VWR
AmAc UA
AmAc VWR
Total Z-activity
Time (min)

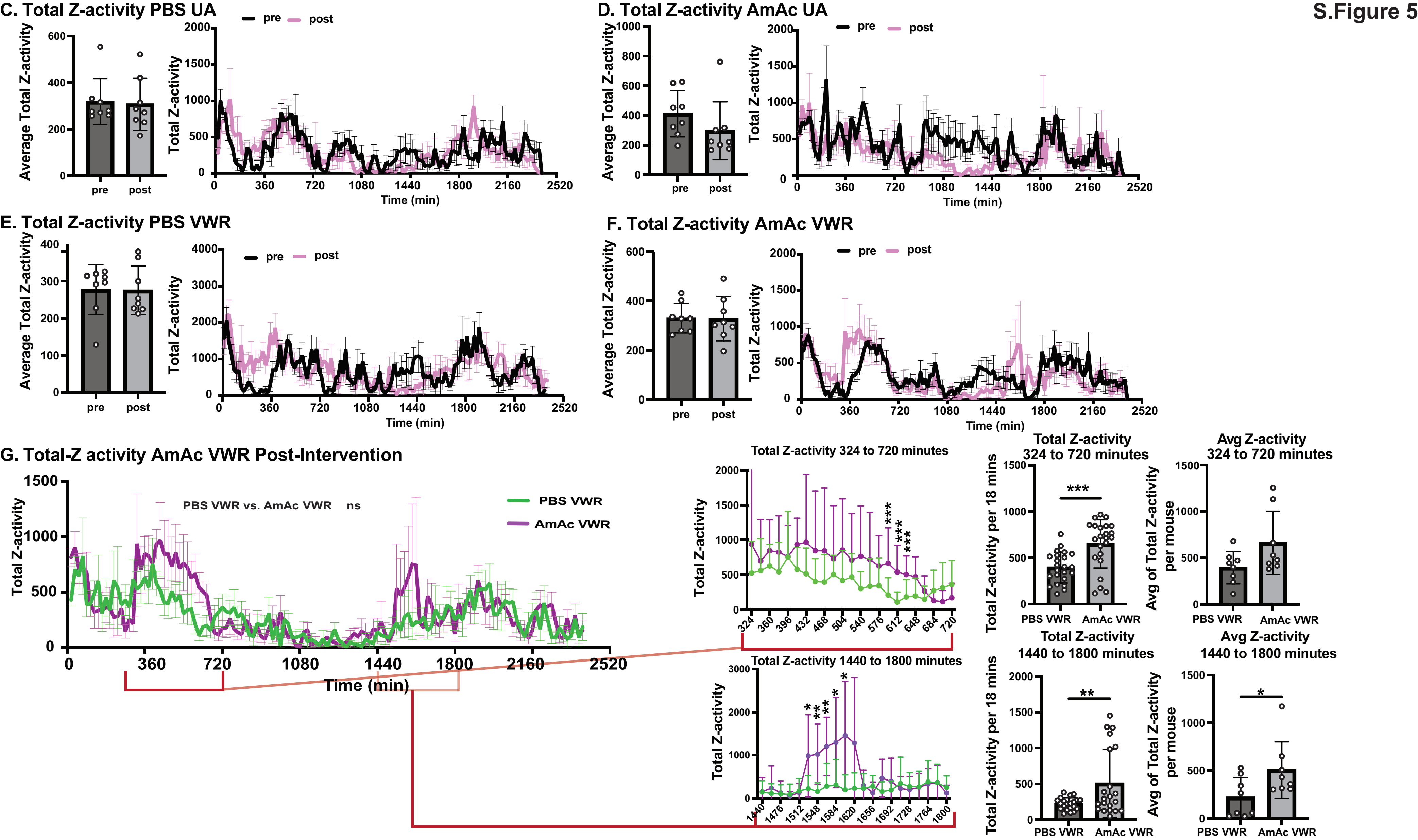
S.Figure 5
C. Total Z-activity PBS UA
D. Total Z-activity AmAc UA
E. Total Z-activity PBS VWR
F. Total Z-activity AmAc VWR
G. Total-Z activity AmAc VWR Post-Intervention
Average Total Z-activity
Total Z-activity
pre
post
Time (min)
PBS VWR vs. AmAc VWR ns
PBS VWR
AmAc VWR
Total Z-activity 324 to 720 minutes
Total Z-activity 1440 to 1800 minutes
Total Z-activity per 18 mins
Avg of Total Z-activity per mouse
Avg Z-activity 324 to 720 minutes
Avg Z-activity 1440 to 1800 minutes

S. Figure 6

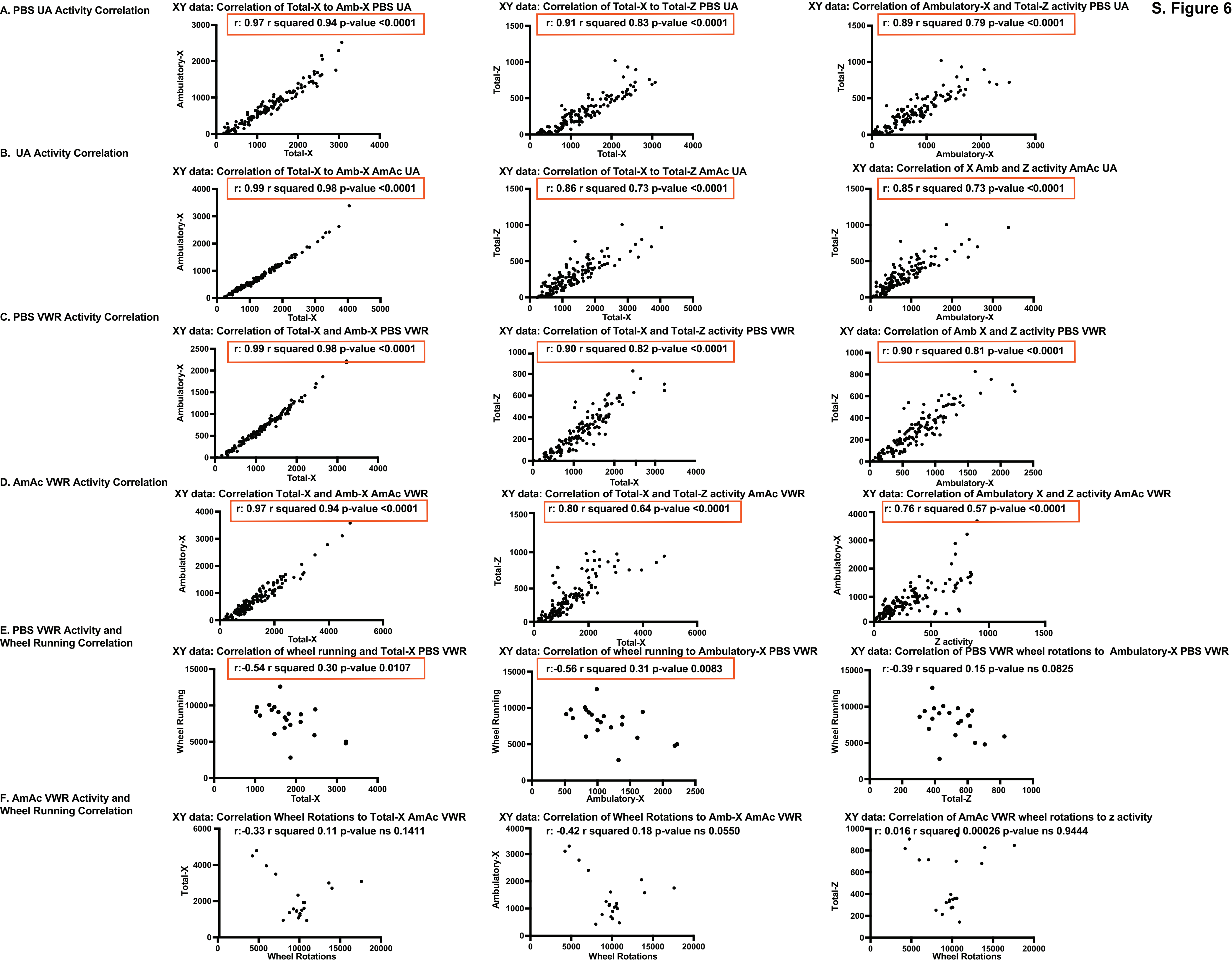
A. PBS UA Activity Correlation
XY data: Correlation of Total-X to Amb-X PBS UA
r: 0.97 r squared 0.94 p-value <0.0001
Ambulatory-X
Total-X
XY data: Correlation of Total-X to Total-Z PBS UA
r: 0.91 r squared 0.83 p-value <0.0001
Total-Z
Total-X
XY data: Correlation of Ambulatory-X and Total-Z activity PBS UA
r: 0.89 r squared 0.79 p-value <0.0001
Total-Z
Ambulatory-X
B. UA Activity Correlation
XY data: Correlation of Total-X to Amb-X AmAc UA
r: 0.99 r squared 0.98 p-value <0.0001
Ambulatory-X
Total-X
XY data: Correlation of Total-X to Total-Z AmAc UA
r: 0.86 r squared 0.73 p-value <0.0001
Total-Z
Total-X
XY data: Correlation of X Amb and Z activity AmAc UA
r: 0.85 r squared 0.73 p-value <0.0001
Total-Z
Ambulatory-X
C. PBS VWR Activity Correlation
XY data: Correlation of Total-X and Amb-X PBS VWR
r: 0.99 r squared 0.98 p-value <0.0001
Ambulatory-X
Total-X
XY data: Correlation of Total-X and Total-Z activity PBS VWR
r: 0.90 r squared 0.82 p-value <0.0001
Total-Z
Total-X
XY data: Correlation of Amb X and Z activity PBS VWR
r: 0.90 r squared 0.81 p-value <0.0001
Total-Z
Ambulatory-X
D. AmAc VWR Activity Correlation
XY data: Correlation Total-X and Amb-X AmAc VWR
r: 0.97 r squared 0.94 p-value <0.0001
Ambulatory-X
Total-X
XY data: Correlation of Total-X and Total-Z activity AmAc VWR
r: 0.80 r squared 0.64 p-value <0.0001
Total-Z
Total-X
XY data: Correlation of Ambulatory X and Z activity AmAc VWR
r: 0.76 r squared 0.57 p-value <0.0001
Ambulatory-X
Z activity
E. PBS VWR Activity and Wheel Running Correlation
XY data: Correlation of wheel running and Total-X PBS VWR
r:-0.54 r squared 0.30 p-value 0.0107
Wheel Running
Total-X
XY data: Correlation of wheel running to Ambulatory-X PBS VWR
r:-0.56 r squared 0.31 p-value 0.0083
Wheel Running
Ambulatory-X
XY data: Correlation of PBS VWR wheel rotations to Ambulatory-X PBS VWR
r:-0.39 r squared 0.15 p-value ns 0.0825
Wheel Running
Total-Z
F. AmAc VWR Activity and Wheel Running Correlation
XY data: Correlation Wheel Rotations to Total-X AmAc VWR
r:-0.33 r squared 0.11 p-value ns 0.1411
Total-X
Wheel Rotations
XY data: Correlation of Wheel Rotations to Amb-X AmAc VWR
r: -0.42 r squared 0.18 p-value ns 0.0550
Ambulatory-X
Wheel Rotations
XY data: Correlation of AmAc VWR wheel rotations to z activity
r: 0.016 r squared 0.00026 p-value ns 0.9444
Total-Z
Wheel Rotations

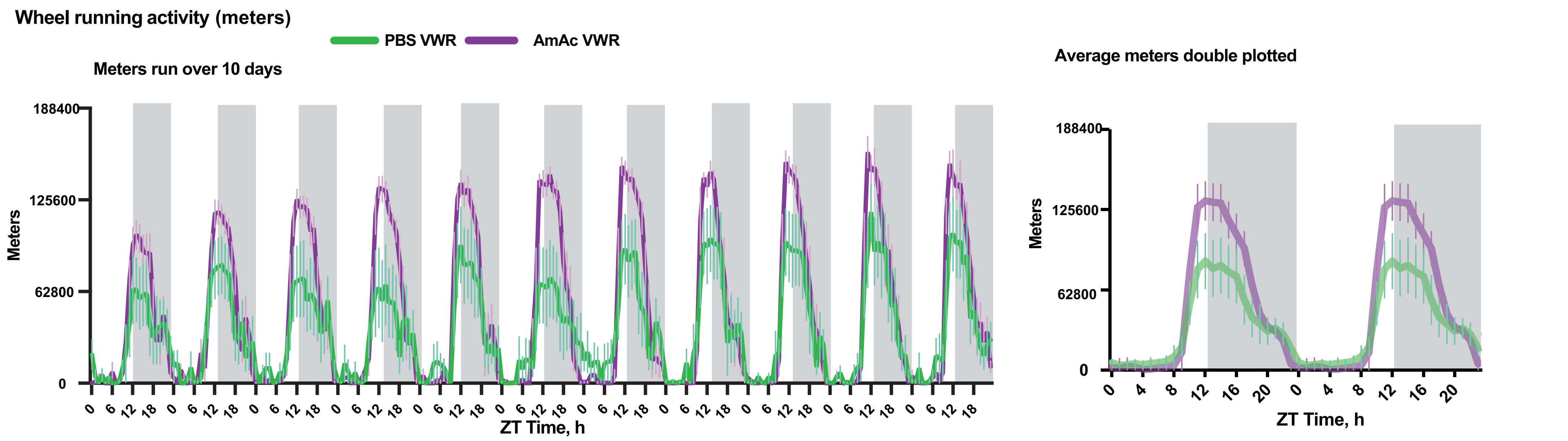
Wheel running activity (meters)
PBS VWR
AmAc VWR
Meters run over 10 days
188400
125600
62800
0
Meters
ZT Time, h
Average meters double plotted
188400
125600
62800
0
Meters
0
4
8
12
16
20
ZT Time, h

S. Figure 8

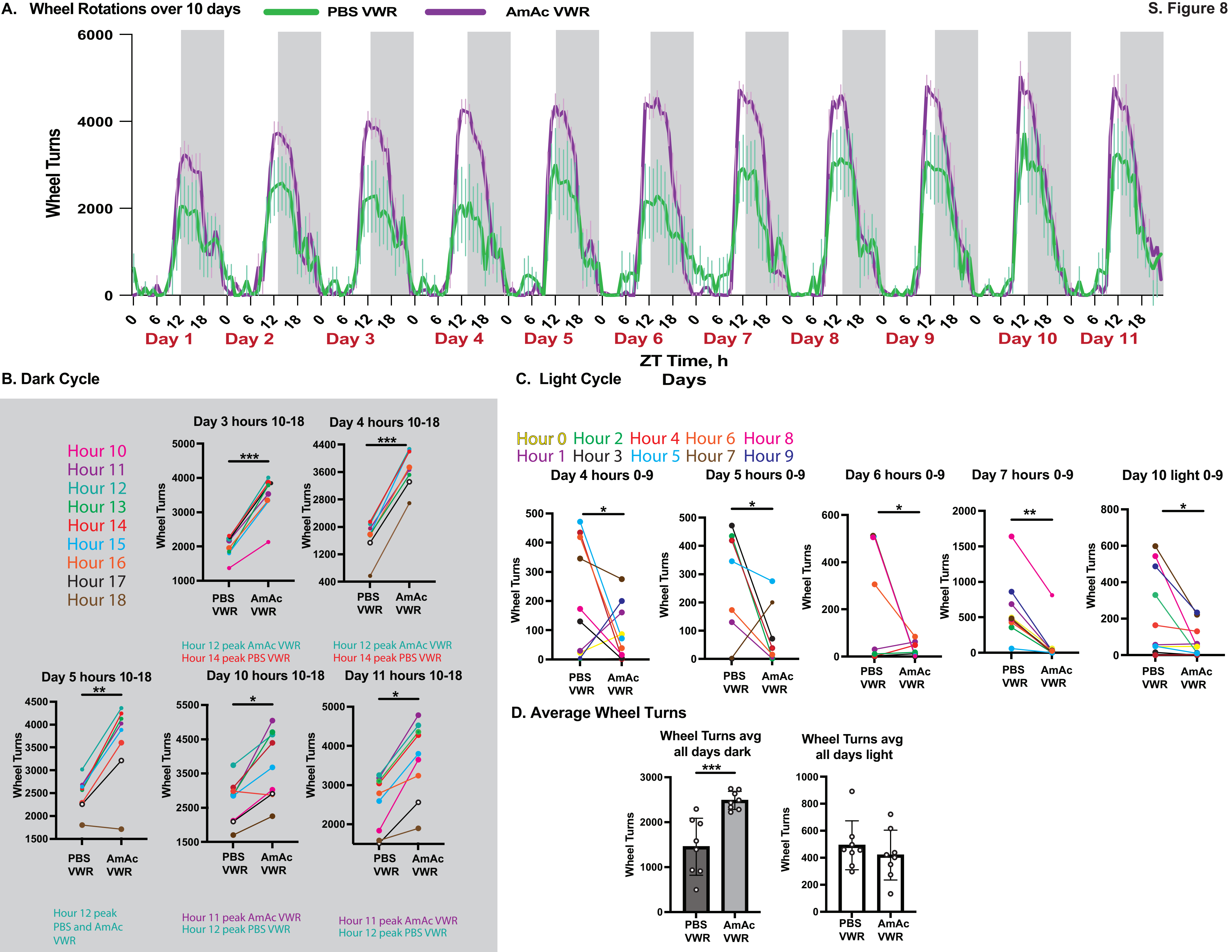

A. Wheel Rotations over 10 days
PBS VWR
AmAc VWR
Wheel Turns
6000
4000
2000
0
0 6 12 18
Day 1
Day 2
Day 3
Day 4
Day 5
Day 6
Day 7
Day 8
Day 9
Day 10
Day 11
ZT Time, h
Days
B. Dark Cycle
Hour 10
Hour 11
Hour 12
Hour 13
Hour 14
Hour 15
Hour 16
Hour 17
Hour 18
Day 3 hours 10-18
Day 4 hours 10-18
Day 5 hours 10-18
Day 10 hours 10-18
Day 11 hours 10-18
PBS VWR
AmAc VWR
Hour 12 peak AmAc VWR
Hour 14 peak PBS VWR
Hour 12 peak AmAc VWR
Hour 14 peak PBS VWR
Hour 12 peak PBS and AmAc VWR
Hour 11 peak AmAc VWR
Hour 12 peak PBS VWR
Hour 11 peak AmAc VWR
Hour 12 peak PBS VWR
C. Light Cycle
Hour 0 Hour 2 Hour 4 Hour 6 Hour 8
Hour 1 Hour 3 Hour 5 Hour 7 Hour 9
Day 4 hours 0-9
Day 5 hours 0-9
Day 6 hours 0-9
Day 7 hours 0-9
Day 10 light 0-9
D. Average Wheel Turns
Wheel Turns avg all days dark
Wheel Turns avg all days light

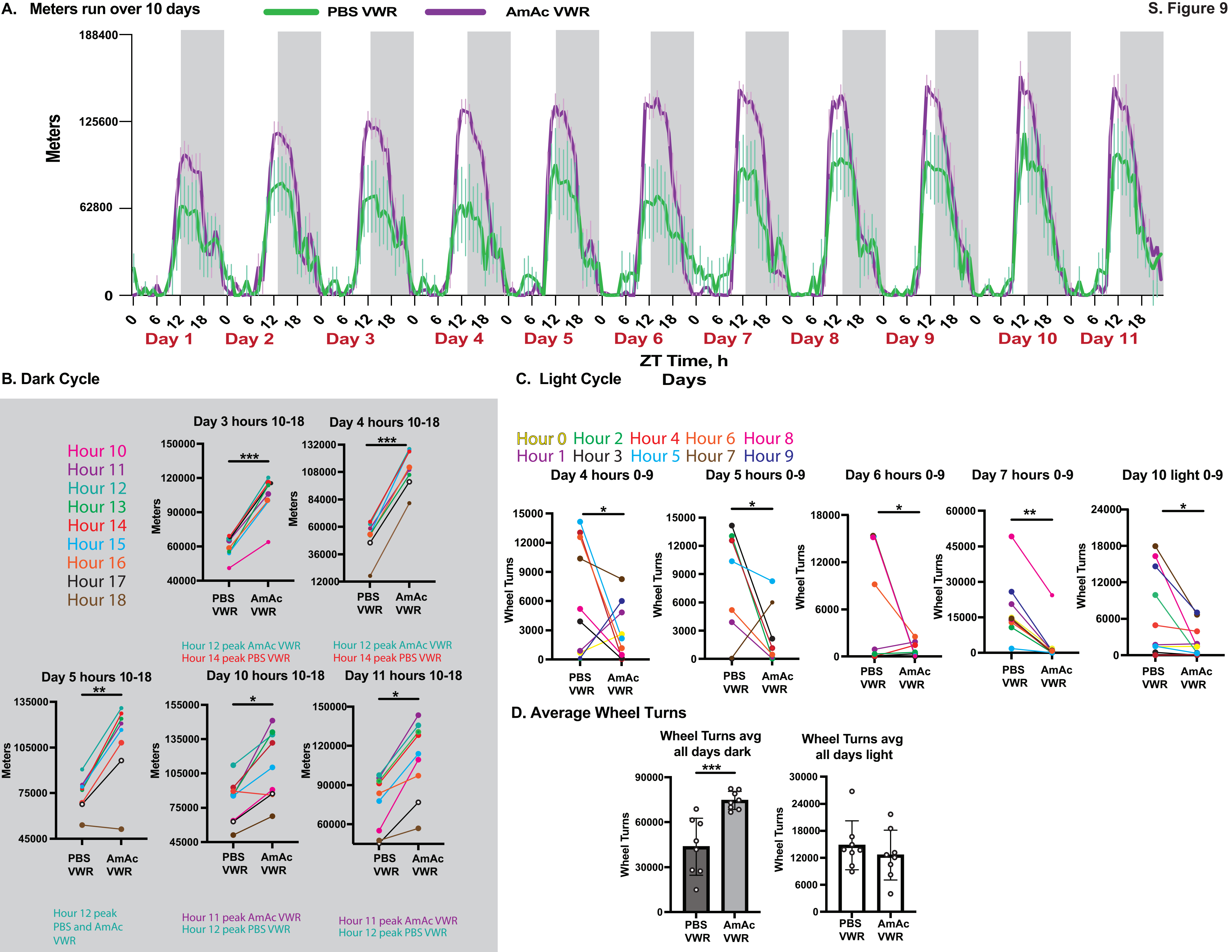

S. Figure 9
A. Meters run over 10 days
PBS VWR
AmAc VWR
Meters
188400
125600
62800
0
Day 1 Day 2 Day 3 Day 4 Day 5 Day 6 Day 7 Day 8 Day 9 Day 10 Day 11
ZT Time, h
Days
B. Dark Cycle
Hour 10
Hour 11
Hour 12
Hour 13
Hour 14
Hour 15
Hour 16
Hour 17
Hour 18
Day 3 hours 10-18
Day 4 hours 10-18
Hour 12 peak AmAc VWR
Hour 14 peak PBS VWR
Day 5 hours 10-18
Day 10 hours 10-18
Day 11 hours 10-18
Hour 12 peak PBS and AmAc VWR
Hour 11 peak AmAc VWR
Hour 12 peak PBS VWR
PBS VWR
AmAc VWR
C. Light Cycle
Hour 0 Hour 2 Hour 4 Hour 6 Hour 8
Hour 1 Hour 3 Hour 5 Hour 7 Hour 9
Day 4 hours 0-9
Day 5 hours 0-9
Day 6 hours 0-9
Day 7 hours 0-9
Day 10 light 0-9
Wheel Turns
D. Average Wheel Turns
Wheel Turns avg all days dark
Wheel Turns avg all days light

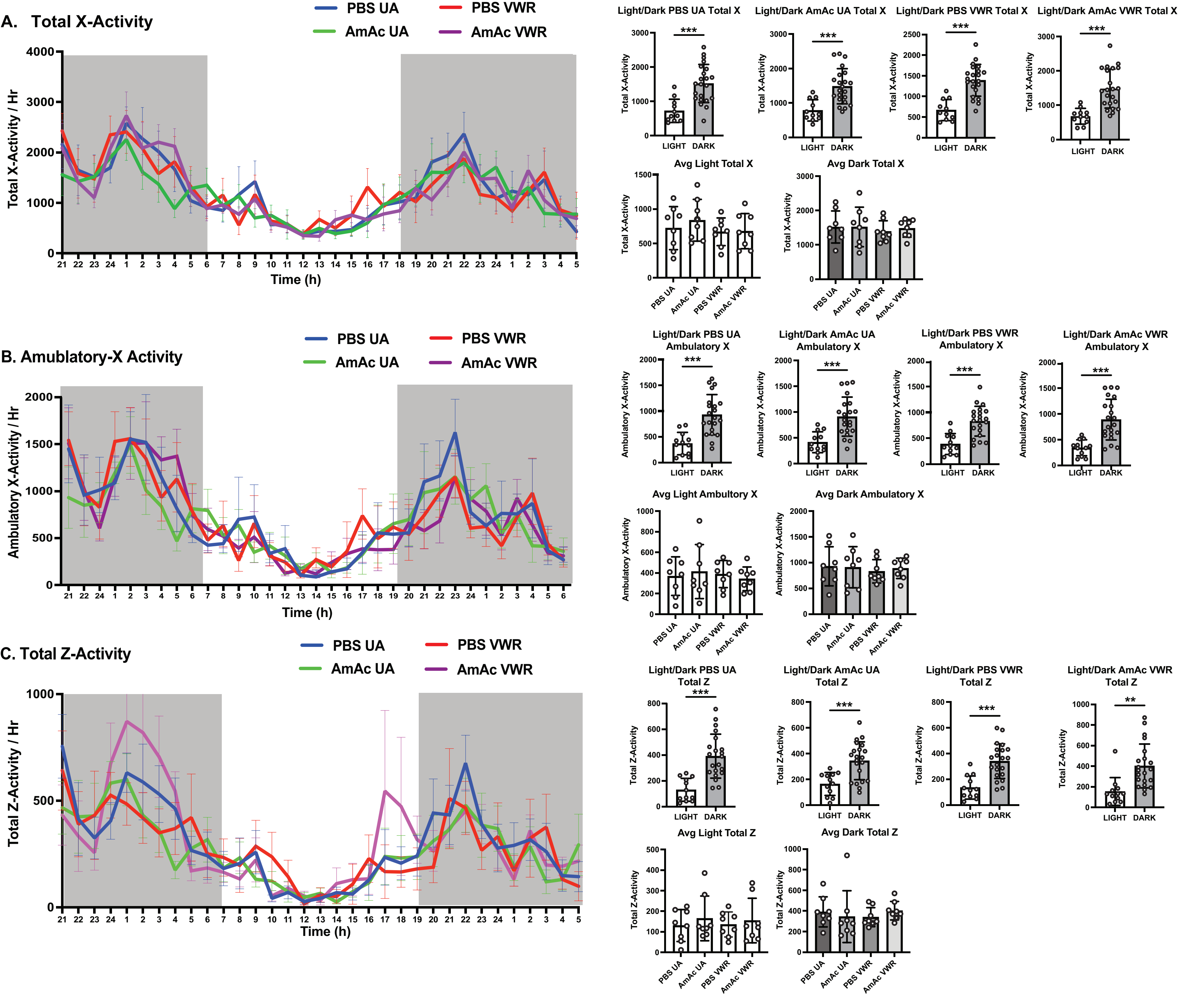
A. Total X-Activity
PBS UA
PBS VWR
AmAc UA
AmAc VWR
Total X-Activity / Hr
Time (h)
Light/Dark PBS UA Total X
Light/Dark AmAc UA Total X
Light/Dark PBS VWR Total X
Light/Dark AmAc VWR Total X
Total X-Activity
LIGHT
DARK
***
Avg Light Total X
Avg Dark Total X
B. Amublatory-X Activity
Ambulatory X-Activity / Hr
Light/Dark PBS UA Ambulatory X
Light/Dark AmAc UA Ambulatory X
Light/Dark PBS VWR Ambulatory X
Light/Dark AmAc VWR Ambulatory X
Ambulatory X-Activity
Avg Light Ambultory X
Avg Dark Ambulatory X
C. Total Z-Activity
Total Z-Activity / Hr
Light/Dark PBS UA Total Z
Light/Dark AmAc UA Total Z
Light/Dark PBS VWR Total Z
Light/Dark AmAc VWR Total Z
Total Z-Activity
**
Avg Light Total Z
Avg Dark Total Z
S. Figure 10

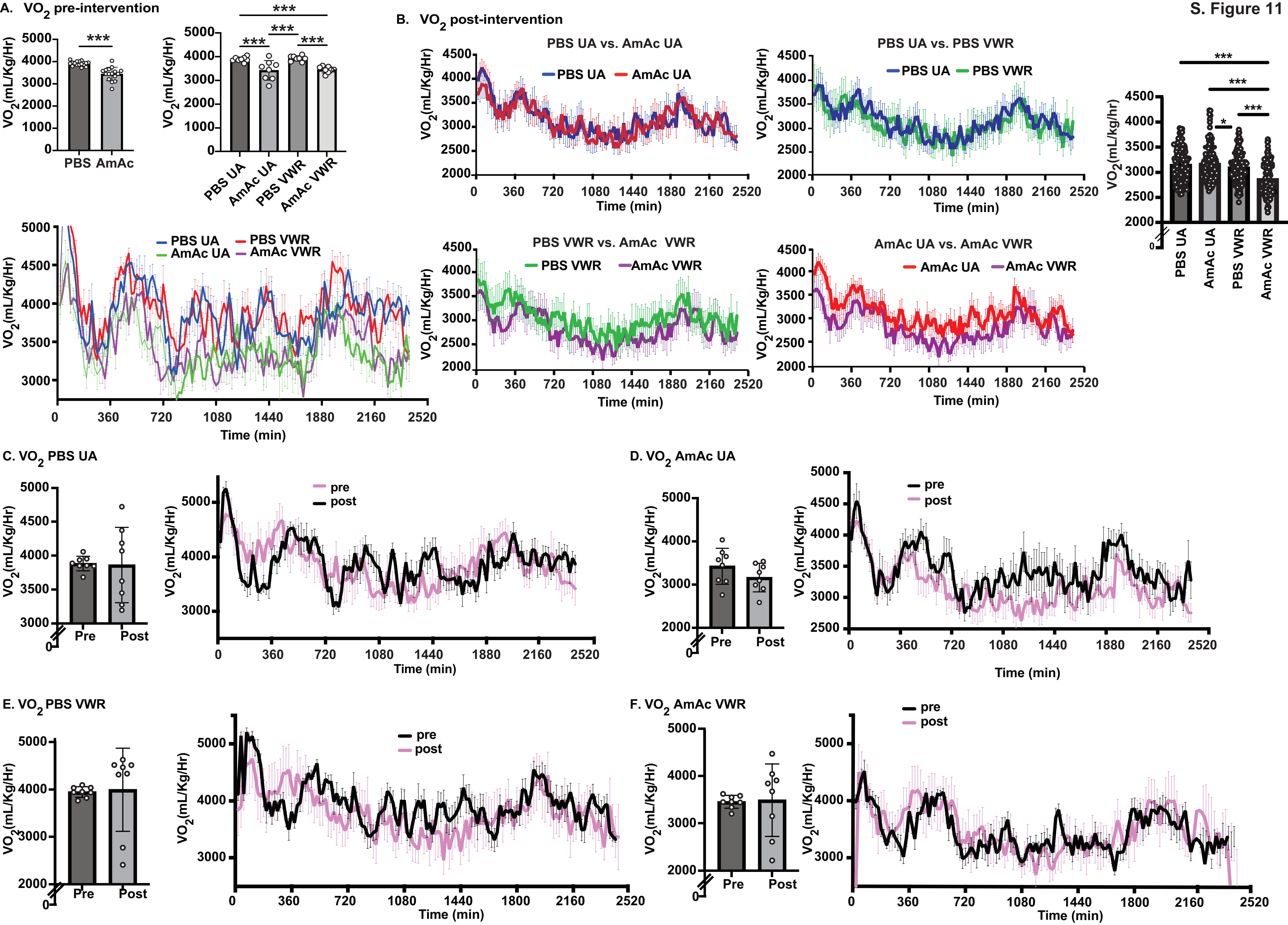
S. Figure 11
A. VO2 pre-intervention
B. VO2 post-intervention
PBS UA vs. AmAc UA
PBS UA vs. PBS VWR
PBS VWR vs. AmAc VWR
AmAc UA vs. AmAc VWR
C. VO2 PBS UA
D. VO2 AmAc UA
E. VO2 PBS VWR
F. VO2 AmAc VWR
PBS
AmAc
PBS UA
AmAc UA
PBS VWR
AmAc VWR
Pre
Post
pre
post
VO2(mL/Kg/Hr)
VO2(mL/kg/hr)
Time (min)

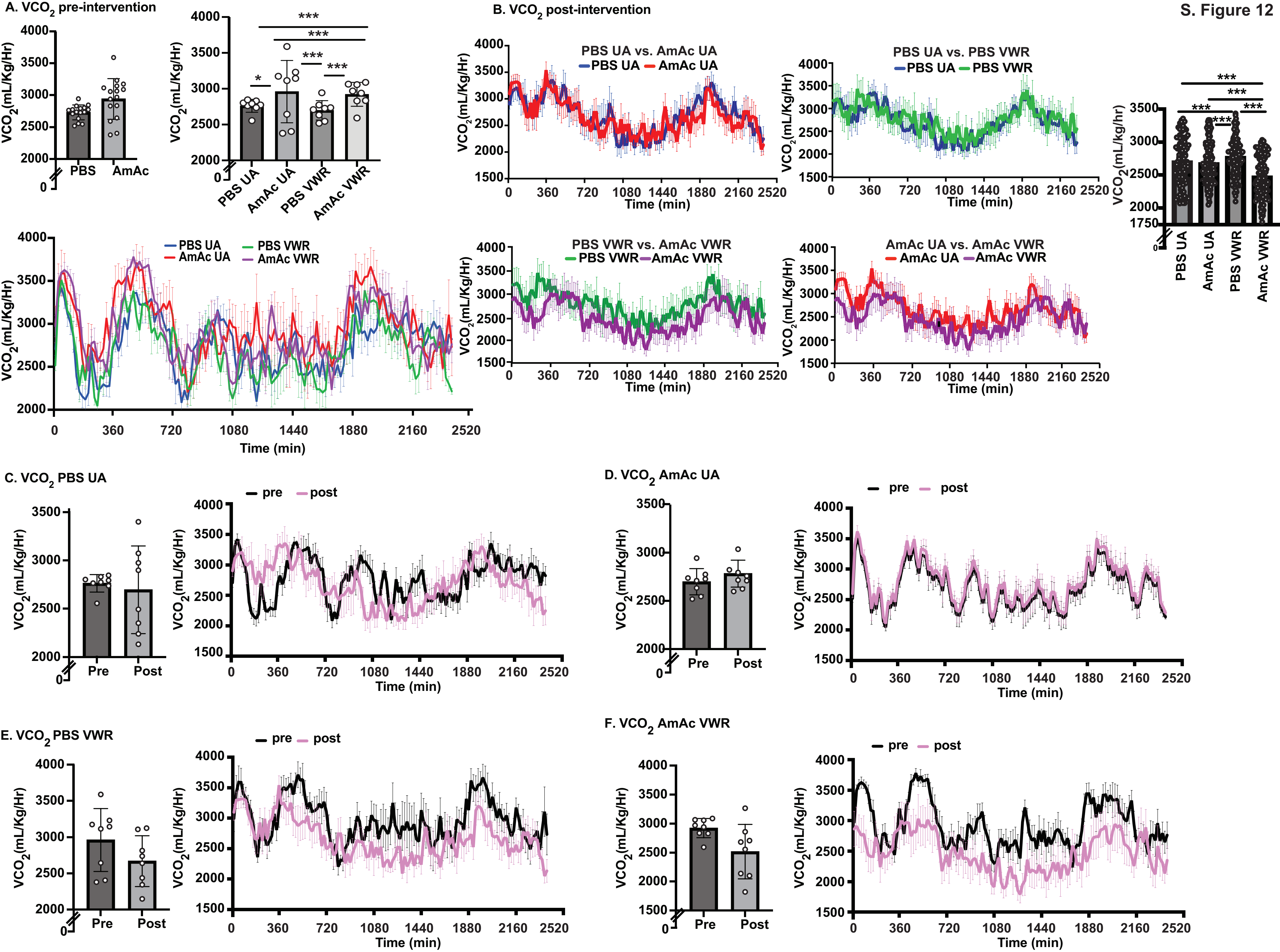
S. Figure 12
A. VCO2 pre-intervention
B. VCO2 post-intervention
PBS UA vs. AmAc UA
PBS UA vs. PBS VWR
PBS VWR vs. AmAc VWR
AmAc UA vs. AmAc VWR
PBS UA
AmAc UA
PBS VWR
AmAc VWR
PBS
AmAc
VCO2(mL/Kg/Hr)
Time (min)
C. VCO2 PBS UA
D. VCO2 AmAc UA
E. VCO2 PBS VWR
F. VCO2 AmAc VWR
pre
post
Pre
Post

S. Figure 13

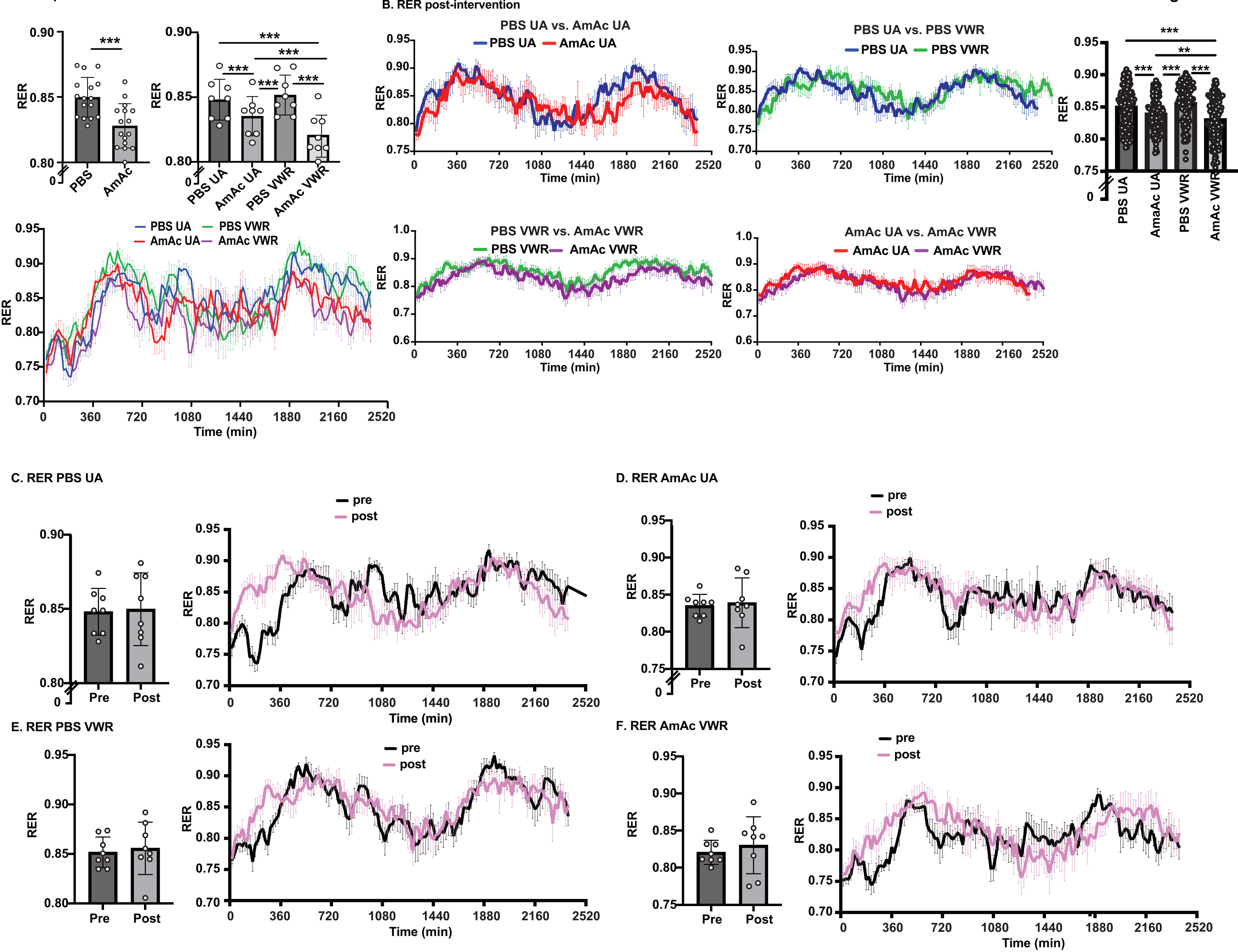

A. RER pre-intervention
B. RER post-intervention
PBS UA vs. AmAc UA
PBS UA vs. PBS VWR
PBS VWR vs. AmAc VWR
AmAc UA vs. AmAc VWR
PBS UA
AmAc UA
PBS VWR
AmAc VWR
PBS
AmAc
RER
Time (min)
C. RER PBS UA
D. RER AmAc UA
E. RER PBS VWR
F. RER AmAc VWR
pre
post
Pre
Post

S. Figure 14

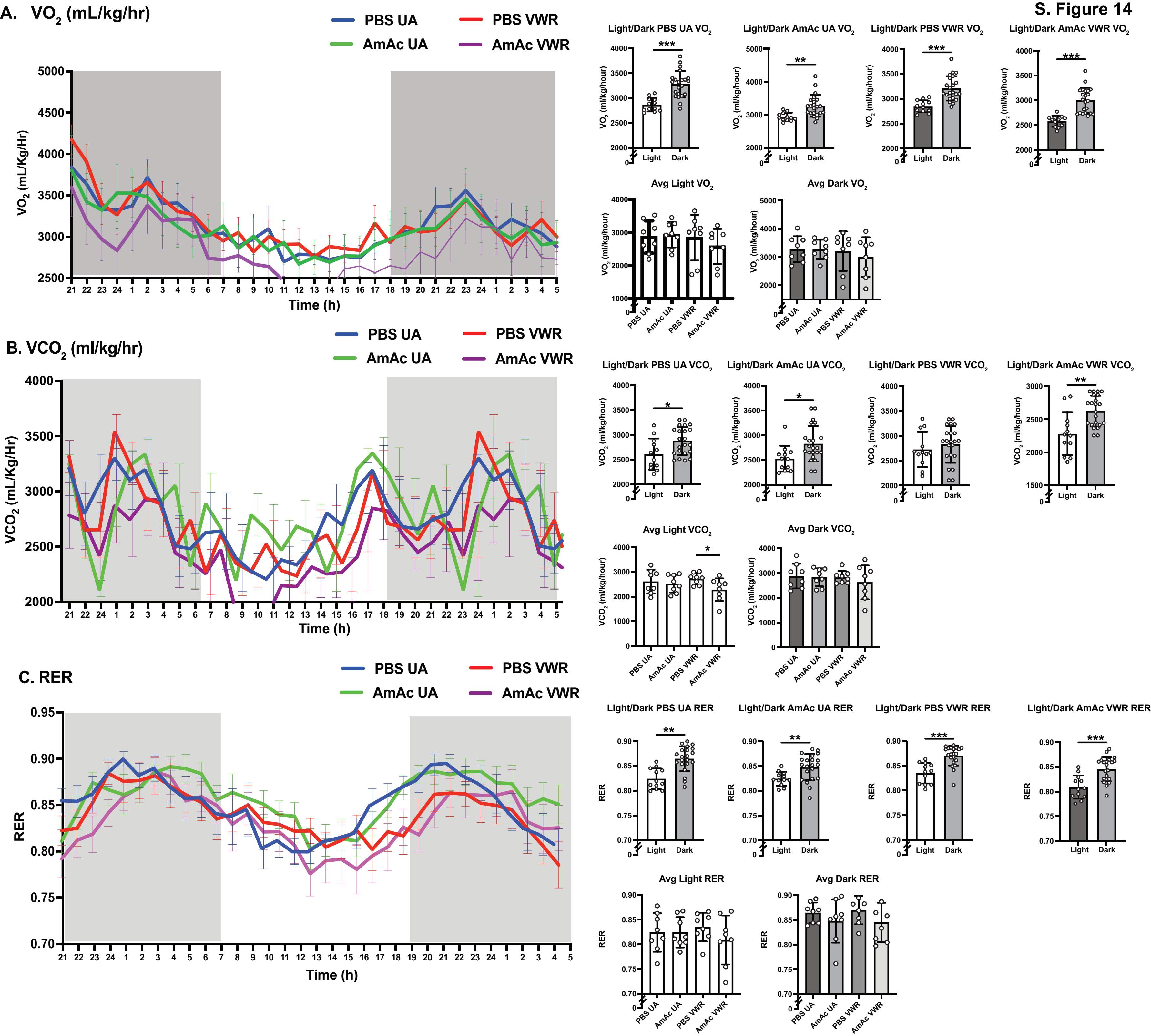

A. VO2 (mL/kg/hr)
PBS UA
PBS VWR
AmAc UA
AmAc VWR
VO2 (mL/Kg/Hr)
Time (h)
Light/Dark PBS UA VO2
Light/Dark AmAc UA VO2
Light/Dark PBS VWR VO2
Light/Dark AmAc VWR VO2
VO2 (ml/kg/hour)
Light
Dark
Avg Light VO2
Avg Dark VO2
B. VCO2 (ml/kg/hr)
VCO2 (mL/Kg/Hr)
Light/Dark PBS UA VCO2
Light/Dark AmAc UA VCO2
Light/Dark PBS VWR VCO2
Light/Dark AmAc VWR VCO2
VCO2 (ml/kg/hour)
Avg Light VCO2
Avg Dark VCO2
C. RER
RER
Light/Dark PBS UA RER
Light/Dark AmAc UA RER
Light/Dark PBS VWR RER
Light/Dark AmAc VWR RER
Avg Light RER
Avg Dark RER

Energy Expenditure (EE) Pre-Intervention

S. Figure 15

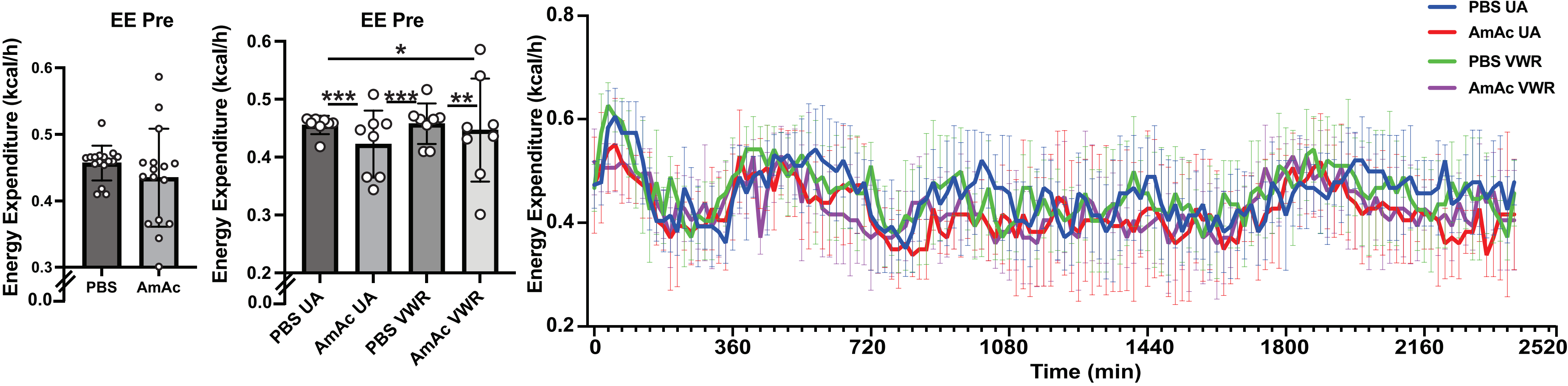

A. Energy expenditure in all mice post-intervention

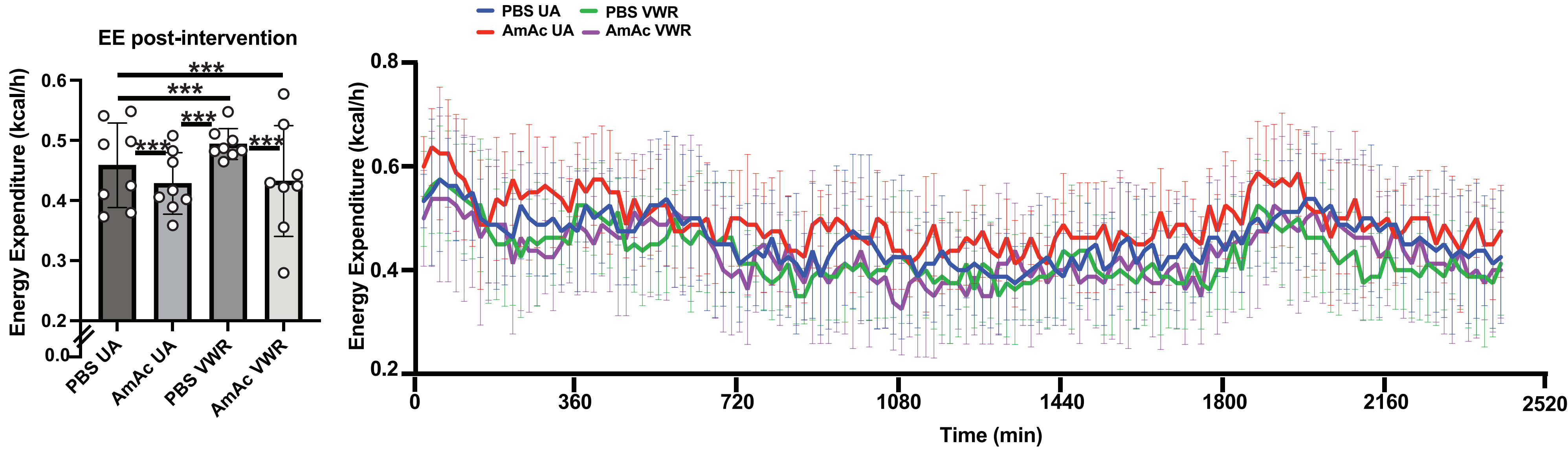

B. Energy expenditure in all mice post-intervention

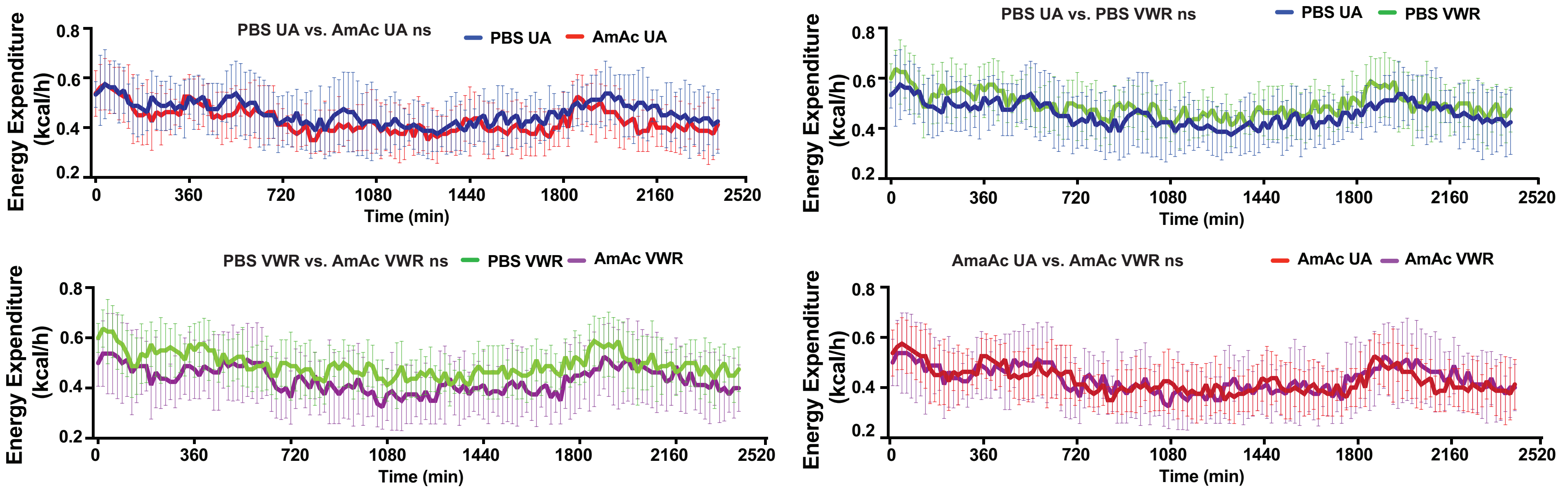

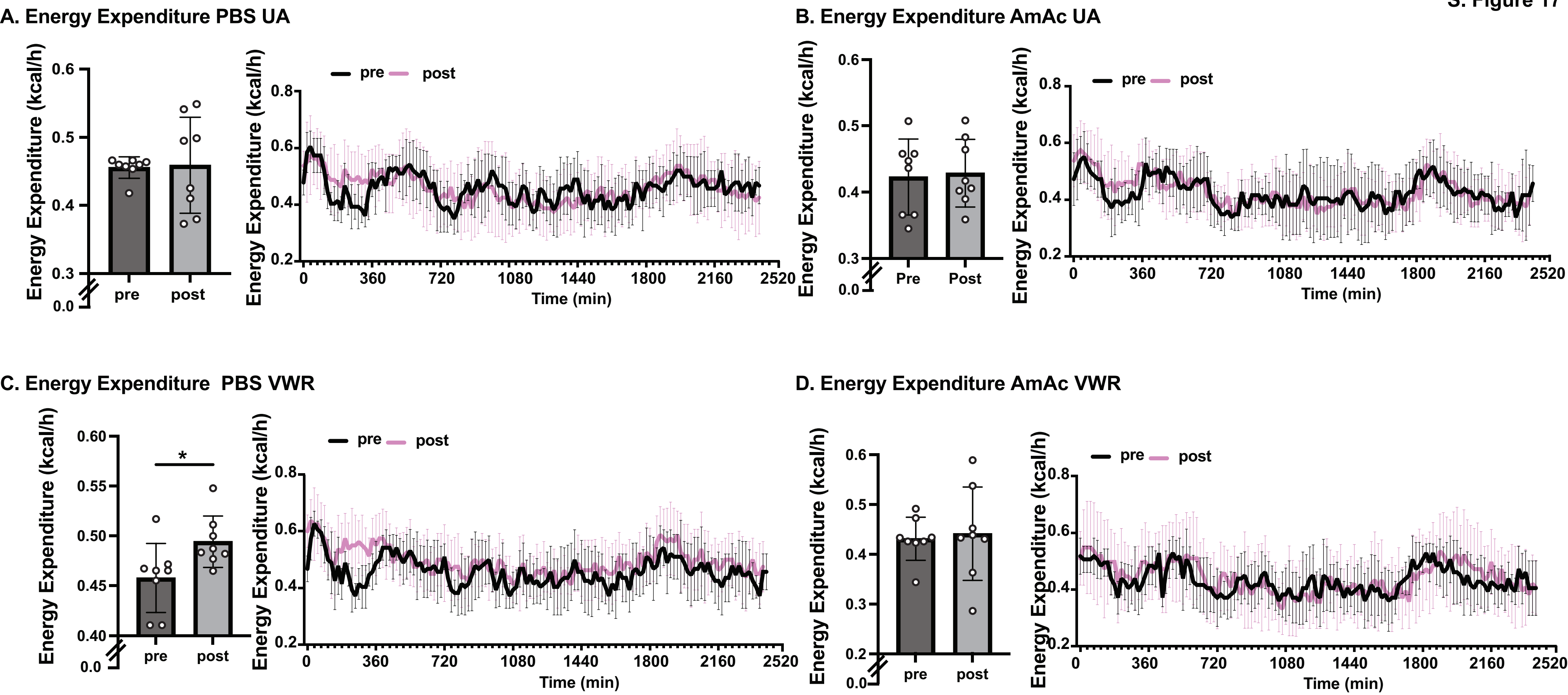
A. Energy Expenditure PBS UA
B. Energy Expenditure AmAc UA
C. Energy Expenditure PBS VWR
D. Energy Expenditure AmAc VWR
Energy Expenditure (kcal/h)
pre
post
Pre
Post
*
Time (min)
0
360
720
1080
1440
1800
2160
2520

S. Fig. 18

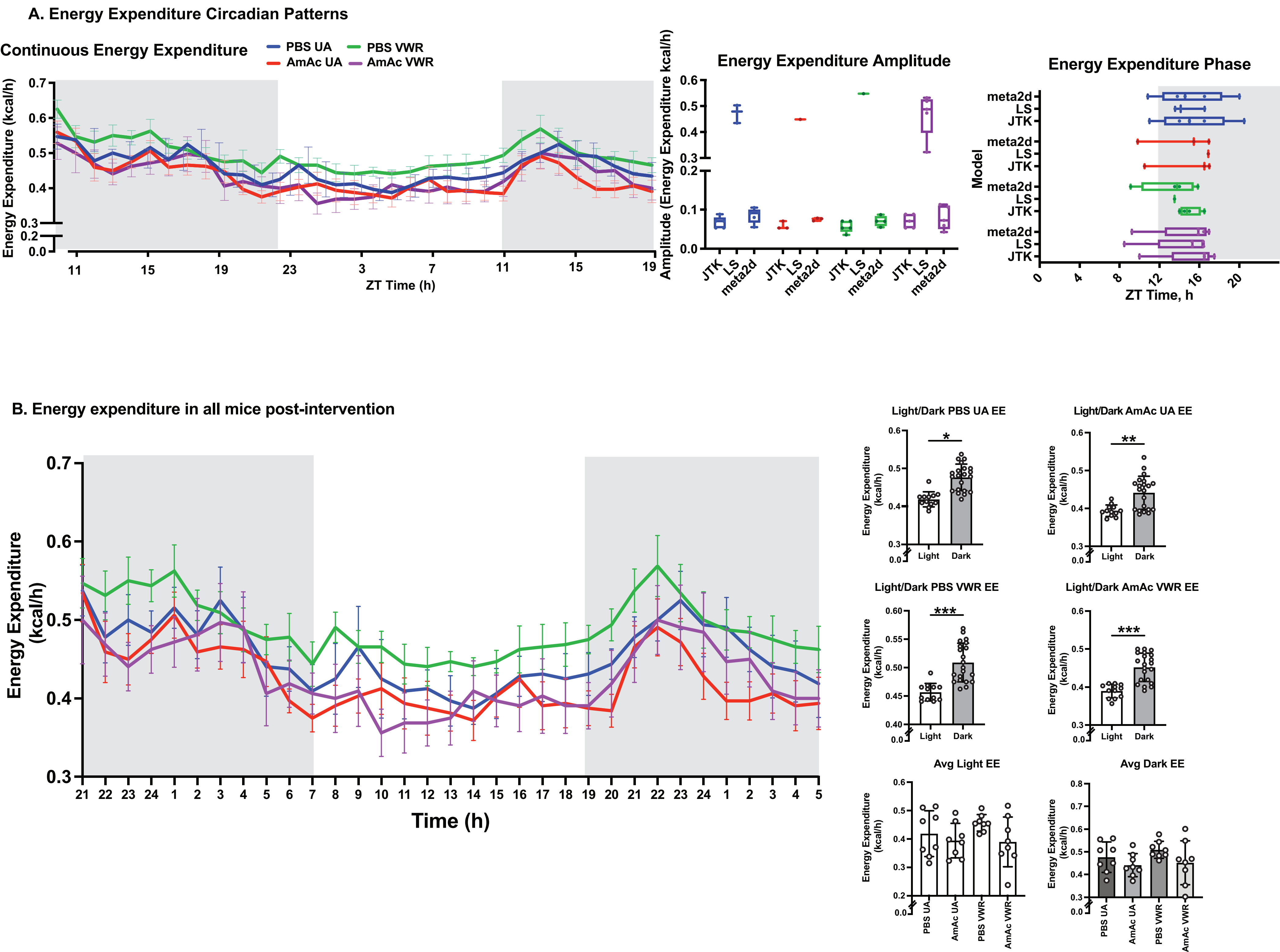
A. Energy Expenditure Circadian Patterns
Continuous Energy Expenditure
PBS UA
AmAc UA
PBS VWR
AmAc VWR
Energy Expenditure (kcal/h)
ZT Time (h)
Energy Expenditure Amplitude
Amplitude (Energy Expenditure kcal/h)
JTK
LS
meta2d
Energy Expenditure Phase
Model
ZT Time, h
B. Energy expenditure in all mice post-intervention
Energy Expenditure (kcal/h)
Time (h)
Light/Dark PBS UA EE
Light/Dark AmAc UA EE
Light/Dark PBS VWR EE
Light/Dark AmAc VWR EE
Energy Expenditure (kcal/h)
Light
Dark
Avg Light EE
Avg Dark EE
PBS UA
AmAc UA
PBS VWR
AmAc VWR

S. Figure 19

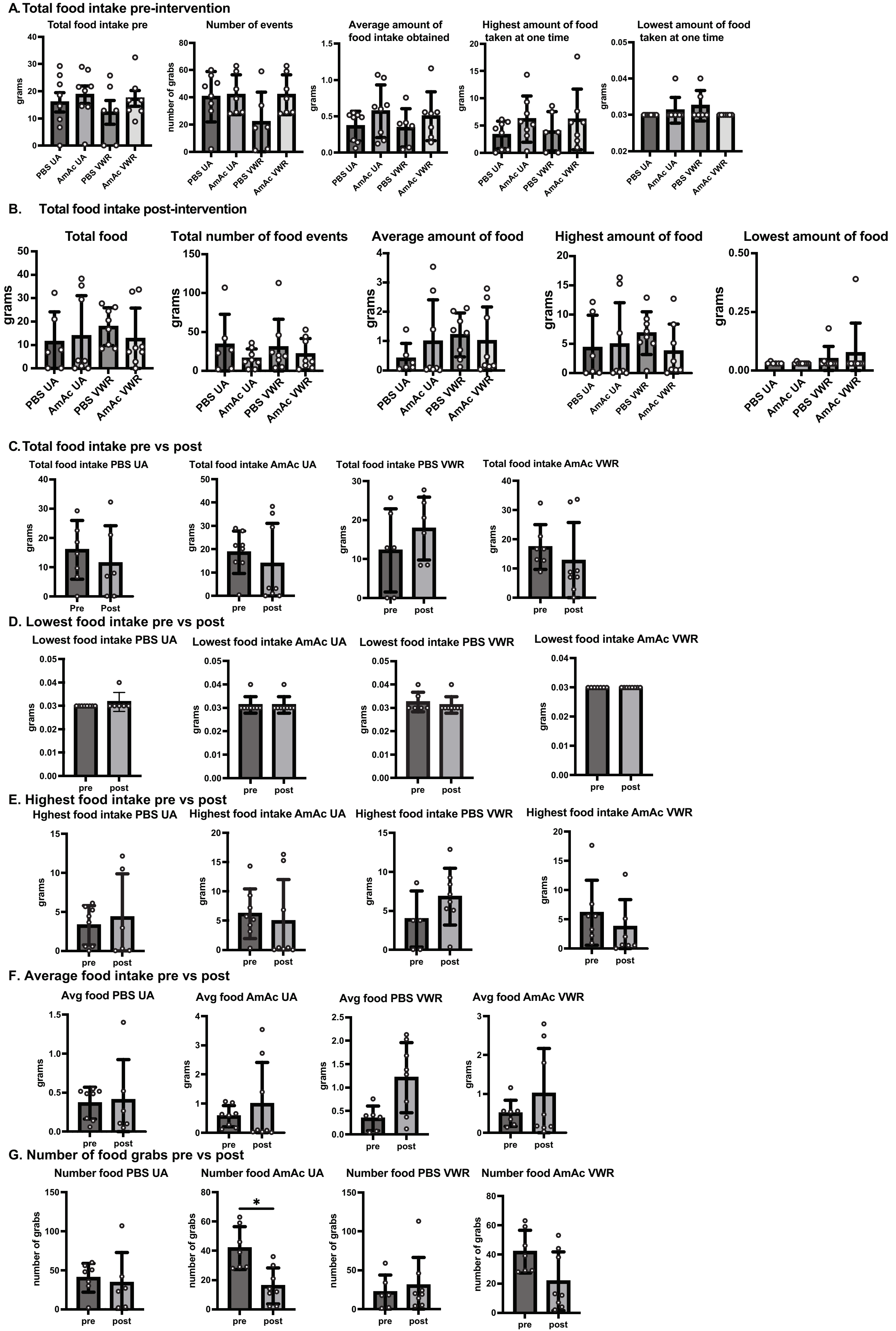
A. Total food intake pre-intervention
Total food intake pre
Number of events
Average amount of food intake obtained
Highest amount of food taken at one time
Lowest amount of food taken at one time
grams
number of grabs
PBS UA
AmAc UA
PBS VWR
AmAc VWR
B. Total food intake post-intervention
Total food
Total number of food events
Average amount of food
Highest amount of food
Lowest amount of food
C. Total food intake pre vs post
Total food intake PBS UA
Total food intake AmAc UA
Total food intake PBS VWR
Total food intake AmAc VWR
Pre
Post
pre
post
D. Lowest food intake pre vs post
Lowest food intake PBS UA
Lowest food intake AmAc UA
Lowest food intake PBS VWR
Lowest food intake AmAc VWR
E. Highest food intake pre vs post
Hghest food intake PBS UA
Highest food intake AmAc UA
Highest food intake PBS VWR
Highest food intake AmAc VWR
F. Average food intake pre vs post
Avg food PBS UA
Avg food AmAc UA
Avg food PBS VWR
Avg food AmAc VWR
G. Number of food grabs pre vs post
Number food PBS UA
Number food AmAc UA
Number food PBS VWR
Number food AmAc VWR
*

Mitochondrial Mass by VDAC and CS

S. Figure 20

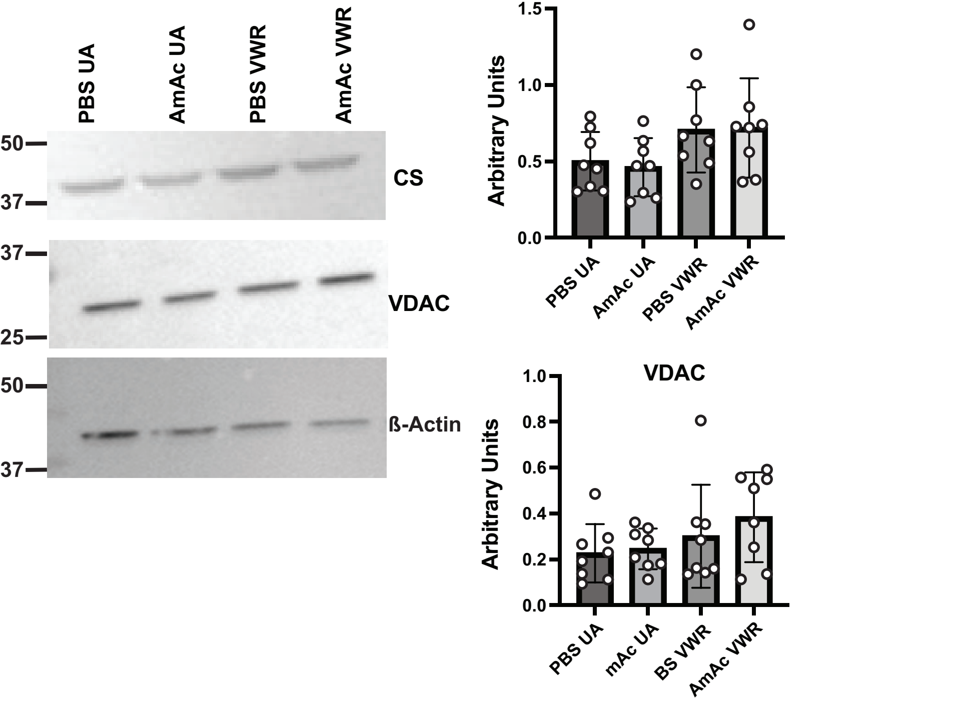

S. Figure 21

A. PBS VWR Correlation Plot

	Running distance	Gastroc weight	Grip strength	Complex I expression	Complex II expression	Puromycin expression	pmTOR expression	pS6 expression	peif2alpha expression	Plasma ammonia	Skeletal muscle ammona	GS expression	AST expression	ADP response	Glutamate response	Succinate response
Running distance	1.00	0.45	0.27	0.27	0.28	0.53	0.47	0.32	0.31		-0.27	-0.59	0.03	-0.24	-0.20	-0.19
Gastroc weight	0.45	1.00	-0.42	0.28	-0.25	0.65	0.38	0.26	0.54	0.47	-0.20	0.21	0.25	0.45	0.56	0.29
Grip strength	0.27	-0.42	1.00	0.37	0.47	0.04	-0.13		-0.22	-0.21	0.40	-0.68	-0.27	-0.17	-0.14	0.14
Complex I expression	0.27	0.28	0.37	1.00	0.32	0.11	-0.29	0.22	-0.37	0.53	0.41	0.06	0.25	-0.12	0.05	-0.03
Complex II expression	0.28	-0.25	0.47	0.32	1.00	0.04	0.43	-0.60	-0.37	-0.25	0.14	-0.12	-0.72	-0.38	-0.30	-0.20
Puromycin expression	0.53	0.65	0.04	0.11	0.04	1.00	0.65	-0.07	0.27	0.41	-0.43	-0.16	-0.02	0.58	0.59	0.56
pmTOR expression	0.47	0.38	-0.13	-0.29	0.43	0.65	1.00	-0.48	0.37	-0.17	-0.50	-0.07	-0.61	0.25	0.24	0.17
pS6 expression	0.32	0.26		0.22	-0.60	-0.07	-0.48	1.00	0.22	0.35	-0.14	-0.21	0.77	-0.05	-0.10	-0.30
peif2alpha expression	0.31	0.54	-0.22	-0.37	-0.37	0.27	0.37	0.22	1.00	-0.35	-0.02	-0.26	-0.12	0.33	0.35	0.30
Plasma ammonia		0.47	-0.21	0.53	-0.25	0.41	-0.17	0.35	-0.35	1.00	-0.33	0.49	0.59	0.45	0.45	0.11
Skeletal muscle ammona	-0.27	-0.20	0.40	0.41	0.14	-0.43	-0.50	-0.14	-0.02	-0.33	1.00	-0.14	-0.13	-0.23	-0.05	0.24
GS expression	-0.59	0.21	-0.68	0.06	-0.12	-0.16	-0.07	-0.21	-0.26	0.49	-0.14	1.00	0.08	0.32	0.34	-0.07
AST expression	0.03	0.25	-0.27	0.25	-0.72	-0.02	-0.61	0.77	-0.12	0.59	-0.13	0.08	1.00	-0.01	-0.04	-0.13
ADP response	-0.24	0.45	-0.17	-0.12	-0.38	0.58	0.25	-0.05	0.33	0.45	-0.23	0.32	-0.01	1.00	0.97	0.75
Glutamate response	-0.20	0.56	-0.14	0.05	-0.30	0.59	0.24	-0.10	0.35	0.45	-0.05	0.34	-0.04	0.97	1.00	0.81
Succinate response	-0.19	0.29	0.14	-0.03	-0.20	0.56	0.17	-0.30	0.30	0.11	0.24	-0.07	-0.13	0.75	0.81	1.00

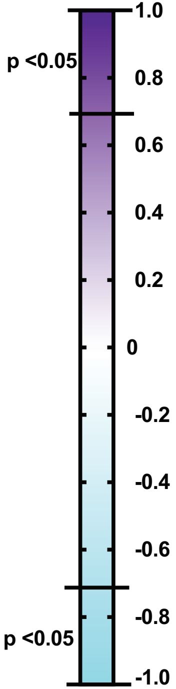

B. AmAc VWR Correlation Plot

	Running distance	Gastroc weight	Grip strength	Complex I expression	Complex II expression	Puromycin expression	pmTOR expression	pS6 expression	peif2alpha expression	Plasma ammonia	Skeletal muscle ammona	GS expression	AST expression	ADP response	Glutamate response	Succinate response
Running distance	1.00	0.76	0.71	0.94	-0.33	0.88	0.87	0.54	0.18	0.08	-0.47	-0.02	-0.68	0.24	0.16	0.08
Gastroc weight	0.76	1.00	0.76	0.69	-0.33	0.54	0.49	0.27	-0.12	0.22	-0.41	0.23	-0.55	0.51	0.46	0.43
Grip strength	0.71	0.76	1.00	0.58	-0.42	0.39	0.51	0.31	-0.29	0.19	0.02	-0.27	-0.10	-0.04	0.12	0.11
Complex I expression	0.94	0.69	0.58	1.00	-0.20	0.87	0.92	0.65	0.19	-0.01	-0.65	0.19	-0.71	0.20	-0.01	-0.10
Complex II expression	-0.33	-0.33	-0.42	-0.20	1.00	0.02	-0.11	-0.10	-0.51	-0.76	0.28	0.19	0.14	-0.23	-0.36	-0.35
Puromycin expression	0.88	0.54	0.39	0.87	0.02	1.00	0.78	0.35	0.29	-0.21	-0.45	-0.04	-0.78	0.13	-0.02	-0.12
pmTOR expression	0.87	0.49	0.51	0.92	-0.11	0.78	1.00	0.84	0.06	-0.08	-0.50	0.17	-0.54	0.11	-0.08	-0.17
pS6 expression	0.54	0.27	0.31	0.65	-0.10	0.35	0.84	1.00	-0.11	0.06	-0.46	0.45	-0.28	0.18	-0.05	-0.10
peif2alpha expression	0.18	-0.12	-0.29	0.19	-0.51	0.29	0.06	-0.11	1.00	0.39	-0.52	-0.11	-0.54	0.15	0.04	-0.03
Plasma ammonia	0.08	0.22	0.19	-0.01	-0.76	-0.21	-0.08	0.06	0.39	1.00	-0.17	-0.03	-0.21	0.28	0.24	0.28
Skeletal muscle ammona	-0.47	-0.41	0.02	-0.65	0.28	-0.45	-0.50	-0.46	-0.52	-0.17	1.00	-0.65	0.71	-0.51	-0.15	-0.07
GS expression	-0.02	0.23	-0.27	0.19	0.19	-0.04	0.17	0.45	-0.11	-0.03	-0.65	1.00	-0.31	0.65	0.30	0.29
AST expression	-0.68	-0.55	-0.10	-0.71	0.14	-0.78	-0.54	-0.28	-0.54	-0.21	0.71	-0.31	1.00	-0.54	-0.23	-0.15
ADP response	0.24	0.51	-0.04	0.20	-0.23	0.13	0.11	0.18	0.15	0.28	-0.51	0.65	-0.54	1.00	0.87	0.85
Glutamate response	0.16	0.46	0.12	-0.01	-0.36	-0.02	-0.08	-0.05	0.04	0.24	-0.15	0.30	-0.23	0.87	1.00	0.99
Succinate response	0.08	0.43	0.11	-0.10	-0.35	-0.12	-0.17	-0.10	-0.03	0.28	-0.07	0.29	-0.15	0.85	0.99	1.00
