# Supplementary material for: Voluntary Activity Wheel Running Improves Hyperammonaemia‐Induced Skeletal Muscle Molecular and Metabolic Perturbations in Mice
Source: J Cachexia Sarcopenia Muscle. 2025 Aug 4;16(4):e70031. doi: 10.1002/jcsm.70031 (PMC12321975; doi:10.1002/jcsm.70031)
Supplement: Supplementary file 4 — Table S1: Key Reagents. Table S2: ART ANOVA p values. Table S3: Serum biochemical data. [file JCSM-16-e70031-s005.docx]

Supplementary Table 1. Key Reagents

| **REAGENT or RESOURCE** | **SOURCE** | **IDENTIFIER** |
| --- | --- | --- |
| **Antibodies** | | |
| Mouse monoclonal anti-β-Actin (C4) | Santa Cruz Biotechnology, Dallas, Texas | Cat# sc-47778 |
| Mouse polyclonal anti-citrate synthase ( PBS UA, AmAc UA, PBS VWR, or AmAc VWR mouse gastrocnemius tissue densitometry 1:2000) | ProteinTech, Rosemont, IL | Cat# 16131 |
| Rabbit polyclonal anti-VDAC (PBS UA, AmAc UA, PBS VWR, or AmAc VWR mouse gastrocnemius tissue densitometry 1:2000) | Cell Signaling Technology, Danvers, MA | Cat# 4866 |
| Total OXHOS Rodent WB Antibody Cocktail | Abcam, Waltham, MA. | Cat# ab110413 |
| Mouse monoclonal anti-puromycin (clone 12D10) (PBS UA, AmAc UA, PBS VWR, or AmAc VWR mouse gastrocnemius tissue densitometry, 1:2000) | EMD Millipore  Corp, Billerica,  MA | Cat# MABE343 |
| Rabbit monoclonal anti-p-mTOR D9C2 XR | Cell Signaling Technology, Danvers, MA | Cat# 5536S |
| Rabbit monoclonal anti-mTOR 7C10 | Cell Signaling Technology, Danvers, MA | Cat# 2983S |
| Rabbit Monoclonal Phospho-S6 Ribosomal Protein (Ser240/244) (D68F8) XP® | Cell Signaling Technology, Danvers, MA | Cat# 5364 |
| Rabbit Monoclonal S6 Ribosomal Protein (5G10) | Cell Signaling Technology, Danvers, MA | Cat# 2217 |
| Rabbit monoclonal p4EBP(236B4) | Cell Signaling Technology, Danvers, MA | Cat# 2855S |
| Rabbit monoclonal 4EBP (53H11) | Cell Signaling Technology, Danvers, MA | Cat# 9644S |
| Rabbit Monoclonal Phospho-AMPKα (Thr172) (D79.5E) | Cell Signaling Technology, Danvers, MA | Cat# 4188 |
| AMPKα (D63G4) Rabbit | Cell Signaling Technology, Danvers, MA | Cat# 5832 |
| Rabbit Monoclonal Phospho-eIF2α (Ser51) (D9G8) XP® Polyclonal glutamate dehydrogenase 1 | Cell Signaling Technology, Danvers, MA | Cat#3398 |
| Rabbit Polyclonal LC3 | NOVUS Centennial, CO | Vat# NB 100-2220 |
| Rabbit monoclonal P62(D1Q5S) | Cell Signaling Technology, Danvers, MA | Cat# 39749S |
| Rabbit Monoclonal Beclin (D40C5) | Cell Signaling Technology, Danvers, MA | Cat# 3495S |
| Rabbit Polyclonal Myostatin | Abcam Waltham, MA. | Cat# AB203076 |
| Rabbit Polyclonal RhBG | Gifted from David Weiner Lab |  |
| Polyclonal glutamate dehydrogenase 1 | ProteinTech, Rosemont, IL | Cat# 14299-1-AP |
| Mouse Monoclonal Glutamic-Oxaloacetic Transaminase 1 or aspartate aminotransferase | GeneTex, Irvine, Ca | Cat# GTX632033 |
| Rabbit Polyclonal Glutamine Synthetase | Abcam, Waltham, MA. | Cat# ab176562 |
| Rabbit Polyclonal GLS-2 | NOVUS, Centennial, CO | Cat# NBP1-54773 |
| Rabbit Polyclonal Pyrroline-5-carboxylate synthase | ProteinTech, Rosemont, IL | Cat# 17719-1-AP |
| Mouse Monoclonal Agrin (D-2) | Santa Cruz Biotechnology, Dallas, Texas | Cat# sc-374117 |
| Anti-Rabbit IgG HRP linked antibody | Cell Signaling Technology, Danvers, MA | Cat# 7076S |
| Anti-Rabbit IgG HRP linked antibody | Cell Signaling Technology, Danvers, MA | Cat# 7074S |
| **Chemicals, peptides, and recombinant proteins** |  |  |
|  | | |
| Ammonium acetate | Sigma-Aldrich, St. Louis, MO | Cat# A7330 |
| Ponceau S solution | Sigma-Aldrich, St. Louis, MO | Cat# SLCQ5486 |
| ECL Western Blotting Detection Reagent | Cytiva, Marlborough, Massachusetts | Cat # RPN2106 |
| Immubilon Western Chemiluminescent HRP Substrate | Milipore Sigma, St. Louis, MO | Cat# WBKLS0500 |
| Precision Plus Protein Kaleidoscope | BioRad ,Richmond, CA | Cat# 1610375 |
| (L)-Malic Acid (Malate) monosodium salt | Sigma-Aldrich, St. Louis, MO | Cat# M11125 |
| Pyruvic Acid sodium Slat (Pyruvate) | Sigma-Aldrich, St. Louis, MO | Cat# P2256 |
| Adenosine diphosphate (ADP) | Sigma-Aldrich, St. Louis, MO | Cat# A2754 |
| Glutamate (L-Glutamic acid, Na Salt) | Sigma-Aldrich, St. Louis, MO | Cat# G5889 |
| Succinate disodium salt, hexahydrate | Sigma-Aldrich, St. Louis, MO | Cat# S2378 |
| Carbonyl cyanide p-trifluoro-methoxyphenyl hydrazone (FCCP) | Sigma-Aldrich, St. Louis, MO | Cat# C2920 |
| Rotenone | Sigma-Aldrich, St. Louis, MO | Cat# R8875 |
| Antimycin a | Sigma-Aldrich, St. Louis, MO | Cat# A8674 |
| (+)-Sodium L-ascorbate (Ascorbate) | Sigma-Aldrich, St. Louis, MO | Cat# A7631 |
| N,N,N,N’-Tetramethyl-p-phenylenediamine dihydrochloride (TMPD) | Sigma-Aldrich, St. Louis, MO | Cat# T3134 |
| Sodium azide | Sigma-Aldrich, St. Louis, MO | Cat# S2002 |
| β-Mercaptoethanol | Sigma-Aldrich, St. Louis, MO | Cat# M3148 |
| **Critical Commercial Assays** |  |  |
| MiR05-kit | O2k-Network Lab, Innsbruck, Austria | Cat# MiPNet22.10 MiR05-Kit |
| Ammonia Assay Kit | Sigma-Aldrich, St. Louis, MO | Cat# AA0100-1KT |
| Mouse Insulin ELISA | Mercodia, Uppsala, Sweden | Cat# 10-1247-01 |
| **Experimental models: organisms/strains** | | |
| C57BL/6J mice | The Jackson Laboratory, Bar Harbor, ME | Cat# 000664 |
| **Products** |  |  |
| Alzet Mini-Osmotic Pump | DURECT corporation, Cupertino, CA | Model 2006 |

| **Software and algorithms** | | |
| --- | --- | --- |
| Adobe Illustrator 2021 | Adobe, San Jose, CA | https://www.adobe.com/products/illustrator.html?sdid=KKQML&mv=search&ef_id=EAIaIQobChMIxbejkY7X8wIVy3xvBB1b7whaEAAYASAAEgKRYfD_BwE:G:s&s_kwcid=AL!3085!3!442365417815!e!!g!!adobe%20illustrator!1711729586!70905759510&gclid=EAIaIQobChMIxbejkY7X8wIVy3xvBB1b7whaEAAYASAAEgKRYfD_BwE |
| DatLab 6 | Oroboros, Innsbruck, Austria | Cat# 27142-01 |
| ImageJ | NIH, Bethesda, MD | https://imagej.nih.gov/ij/ |

**Supplementary Table 2 ART ANOVA p-values**

| ART ANOVA table | Degrees of freedom | F value | P values |
| --- | --- | --- | --- |
| Total X-activity pre factor 1 | 1 | 1.3272968 | 0.24935 |
| (UA vs VWR) |  |  |  |
| Total X-activity pre factor 2 | 1 | 0.0013009 | 0.97123 |
| (PBS vs AmAc) |  |  |  |
| Total X-activity pre interaction of factor 1 and factor 2 | 1 | 0.1088008 | 0.74153 |
| Total X-activity post factor 1 | 1 | 0.72084 | 0.39592 |
| (UA vs VWR) |  |  |  |
| Total X-activity post factor 2  PBS vs AmAc | 1 | 1.66133 | 0.19749 |
| Total X-activity post interaction of factor 1 and factor 2 | 1 | 1.40864 | 0.23535 |
| Ambulatory X-activity pre factor 1 | 1 | 0.029461 | 0.86373 |
| (UA vs VWR) |  |  |  |
| Ambulatory X-activity pre factor 2 | 1 | 0.155212 | 0.69362 |
| (PBS vs AmAc) |  |  |  |
| Ambulatory X-activity pre interaction of factor 1 and factor 2 | 1 | 0.257094 | 0.61215 |
| Ambulatory X-activity post factor 1 | 1 | 0.046799 | 0.82874 |
| (UA vs VWR) |  |  |  |
| Ambulatory X-activity post factor 2 | 1 | 0.85974 | 0.35386 |
| (PBS vs AmAc) |  |  |  |
| Ambulatory X-activity post interaction of factor 1 and factor 2 | 1 | 0.318098 | 0.57278 |
| Z-activity pre factor 1 (UA vs VWR) | 1 | 16.1863 | **5.84E-05** |
|  |  |  |  |
| Z-activity pre factor 2 (PBS vs AmAc) | 1 | 35.962 | **2.18E-09** |
|  |  |  |  |
| Z-activity pre interaction of factor 1 and factor 2 | 1 | 2.5419 | 0.11094 |
|  |  |  |  |
| Z-activity post factor 1 (UA vs VWR) | 1 | 0.43573 | 0.509226 |
|  |  |  |  |
| Z-activity post factor 2 (PBS vs AmAc) | 1 | 0.20028 | 0.654521 |
|  |  |  |  |
| Z-activity post interaction of factor 1 and factor 2 | 1 | 6.06125 | **0.013858** |
| Running Distance factor 1 (PBS-VWR vs AmAc-VWR) | 1 | 0.01029 | 0.91956 |
| Running Distance factor 2 (weeks 1-4) | 3 | 3.23547 | **0.0289** |
| Running Distance interaction of factor 1 and factor 2 | 3 | 0.6758 | 0.57051 |
| VO_2_ pre factor 1  (UA vs VWR) | 1 | 3.98823 | **1.44E-13** |
| VO_2_ pre factor 2  (PBS vs AmAc) | 1 | 569.40655 | **<2.22E-16** |
| VO_2_ pre interaction of factor 1 and factor 2 | 1 | 0.15023 | 0.698338 |
| VO_2_ post factor 1  (PBS vs AmAc) | 1 | 59.322 | **1.65E-14** |
| VO_2_ post factor 1  (UA vs VWR) | 1 | 23.969 | **1.023E-14** |
| VO_2_ post interaction factor 1 and factor 2 | 1 | 32.436 | **1.31E-8** |
| VCO_2_ pre factor 1  (UA vs VWR) | 1 | 0.21793 | 0.64064496 |
| VCO_2_ pre factor 2  (PBS vs AmAc) | 1 | 52.20513 | **5.90E-13** |
| VCO_2_ pre interaction factor 1 and factor 2 | 1 | 12.85473 | **0.00034041** |
| VCO_2_ post factor 1  (UA vs VWR) | 1 | 7.2691 | **0.0070426** |
| VCO_2_ post factor 2  (UA vs VWR) | 1 | 69.3762 | **<2.22E-16** |
| VCO_2_ post interaction factor 1 and factor 2 | 1 | 55.0144 | 0.86383 |
| RER pre factor 1 | 1 | 8.5504 | **0.0034727** |
| (UA vs VWR) |  |  |  |
| RER pre factor 2 | 1 | 146.3866 | **< 2.22E-16** |
| (PBS vs AmAc) |  |  |  |
| RER pre interaction of factor 1 and factor 2 | 1 | 23.173 | **1.53E-06** |
| RER post factor 1  (UA vs VWR) | 1 | 0.61512 | 0.43291154 |
| RER post factor 2  (PBS vs AmAc) | 1 | 84.66421 | **<2.22E-16** |
| RER post interaction of factor 1 and factor 2 | 1 | 12.14655 | **0.00049676** |

**Supplementary Table 3 Serum biochemical data**

|  | **PBS UA** | **AmAc UA** | **PBS VWR** | **AmAc VWR** |
| --- | --- | --- | --- | --- |
| ALT (U/L) | 30 + 7 | 43 + 42 | 32 + 5 | 36 + 14 |
| AST (U/L) | 60 + 23 | 75 + 47 | 59 + 13 | 72 + 35 |
| BUN (mg/dL) | 26 + 3 | 28 + 3 | 32 + 7^a^ | 28 + 4 |
| Glucose (mg/dL) | 301 + 35 | 357 + 25 | 314 + 73 | 331 + 71 |
| Insulin (μg/L) | 0.70 + 0.40 | 0.51 + 0.12 | 0.63 + 0.09 | 0.50 + 0.12 |

Abbreviations: ALT: alanine aminotransferase, AmAc: Ammonia acetate, AST: aspartate aminotransferase; BUN: Blood Urea Nitrogen, dL: deciliter, mg: milligram, L: liter, PBS: phosphate buffered saline, U: units, UA: Usual Activity, μg: microgram, VWR: voluntary wheel running. Statistical analysis was performed using one way ANOVA followed by Fischer’s LSD, a indicates significance of p <0.05 between PBS UA and PBS VWR.
