# Supplementary material for: Voluntary Activity Wheel Running Improves Hyperammonaemia‐Induced Skeletal Muscle Molecular and Metabolic Perturbations in Mice
Source: J Cachexia Sarcopenia Muscle. 2025 Aug 4;16(4):e70031. doi: 10.1002/jcsm.70031 (PMC12321975; doi:10.1002/jcsm.70031)
Supplement: Supplementary file 6 — Data S3: Supplementary References. [file JCSM-16-e70031-s006.docx]

**Supplementary References**

S1. Adeva MM, Souto G, Blanco N, Donapetry C. Ammonium metabolism in humans. Metabolism. 2012;61:1495-511. doi:10.1016/j.metabol.2012.07.007

S2. Eriksson LS, Broberg S, Bjorkman O, Wahren J. Ammonia metabolism during exercise in man. Clin Physiol. 1985;5:325-36. doi:10.1111/j.1475-097x.1985.tb00753.x

S3. Welch N, Singh SS, Musich R, Mansuri MS, Bellar A, Mishra S, et al. Shared and unique phosphoproteomics responses in skeletal muscle from exercise models and in hyperammonemic myotubes. iScience. 2022;25:105325. doi:10.1016/j.isci.2022.105325

S4. Tezuka T, Inoue A, Hoshi T, Weatherbee SD, Burgess RW, Ueta R, Yamanashi Y. The MuSK activator agrin has a separate role essential for postnatal maintenance of neuromuscular synapses. Proc Natl Acad Sci U S A. 2014;111:16556-61. doi:10.1073/pnas.1408409111

S5. Tschop MH, Speakman JR, Arch JR, Auwerx J, Bruning JC, Chan L, et al. A guide to analysis of mouse energy metabolism. Nat Methods. 2011;9:57-63. doi:10.1038/nmeth.1806

S6. von Manteuffel SR, Gingras AC, Ming XF, Sonenberg N, Thomas G. 4E-BP1 phosphorylation is mediated by the FRAP-p70s6k pathway and is independent of mitogen-activated protein kinase. Proc Natl Acad Sci U S A. 1996;93:4076-80. doi:10.1073/pnas.93.9.4076

S7. Goh J, Ladiges WC. A novel long term short interval physical activity regime improves body composition in mice. BMC Res Notes. 2013;6:66. doi:10.1186/1756-0500-6-66

S8. Venezia AC, Hyer MM, Glasper ER, Roth SM, Quinlan EM. Acute forced exercise increases Bdnf IV mRNA and reduces exploratory behavior in C57BL/6J mice. Genes Brain Behav. 2020;19:e12617. doi:10.1111/gbb.12617

S9. Buch BT, Halling JF, Ringholm S, Gudiksen A, Kjobsted R, Olsen MA, et al. Colchicine treatment impairs skeletal muscle mitochondrial function and insulin sensitivity in an age-specific manner. FASEB J. 2020;34:8653-70. doi:10.1096/fj.201903113RR

S10. Kim HJ, Kim YJ, Seong JK. AMP-activated protein kinase activation in skeletal muscle modulates exercise-induced uncoupled protein 1 expression in brown adipocyte in mouse model. J Physiol. 2022;600:2359-76. doi:10.1113/JP282999

S11. Knuiman P, Hopman MTE, Verbruggen C, Mensink M. Protein and the Adaptive Response With Endurance Training: Wishful Thinking or a Competitive Edge? Front Physiol. 2018;9:598. doi:10.3389/fphys.2018.00598
